# Genetic Polymorphisms and Antioxidant Reactions in Prostate Cancer

**DOI:** 10.3390/ijms27083569

**Published:** 2026-04-16

**Authors:** Piotr Kamiński, Joanna Dróżdż-Afelt, Edward Jacek Gorzelańczyk, Jędrzej Baszyński, Halina Tkaczenko, Martin Hromada, Jarosław Nuszkiewicz, Alina Woźniak, Natalia Kurhaluk

**Affiliations:** 1 Division of Ecology and Environmental Protection, Department of Medical Biology and Biochemistry, Faculty of Medicine, Collegium Medicum in Bydgoszcz, Nicolaus Copernicus University in Toruń, M. Skłodowska-Curie St. 9, PL 85-094 Bydgoszcz, Poland; jedrzej.baszynski@cm.umk.pl; 2 Department of Biotechnology, Institute of Biological Sciences, University of Zielona Góra, Prof. Z. Szafran St. 1, PL 65-516 Zielona Góra, Poland; 3 Department of Biotechnology, Kazimierz Wielki University in Bydgoszcz, Księcia Józefa Poniatowskiego St. 12, PL 85-671 Bydgoszcz, Poland; joanna.drozdz@ukw.edu.pl; 4 Institute of Philosophy, Kazimierz Wielki University in Bydgoszcz, 85-064 Bydgoszcz, Poland; medsystem@medsystem.com.pl; 5 Faculty of Mathematics and Computer Science, Adam Mickiewicz University in Poznań, 61-614 Poznań, Poland; 6 Medically Assisted Recovery Association “MAR”, 85-067 Bydgoszcz, Poland; 7 Oskar Bielawski Greater Poland Neuropsychiatric Center in Kościan, 64-000 Kościan, Poland; 8 Institute of Biology, Pomeranian University in Słupsk, Arciszewski St. 22 B, PL 76-200 Słupsk, Poland; halina.tkaczenko@upsl.edu.pl (H.T.); natalia.kurhaluk@upsl.edu.pl (N.K.); 9 Laboratory and Museum of Evolutionary Ecology, Department of Ecology, Faculty of Humanities and Natural Sciences, University of Prešov, 17. Novembra 1, SK-081 16 Prešov, Slovakia; martin.hromada@unipo.sk; 10 Department of Nature Protection and Biodiversity, Institute of Biological Sciences, University of Zielona Góra, Prof. Z. Szafran St. 1, PL 65-516 Zielona Góra, Poland; 11 Division of Medical Biology, Department of Medical Biology and Biochemistry, Faculty of Medicine, Collegium Medicum in Bydgoszcz, Nicolaus Copernicus University in Toruń, M. Karłowicz St. 24, PL 85-092 Bydgoszcz, Poland; al1103@cm.umk.pl

**Keywords:** prostate cancer, male reproductive system, genetic polymorphisms, glutathione S-transferase, antioxidant enzymes, trace elements, MDA, oxidative stress, biodegradation, environmental impact

## Abstract

This review aggregates the latest reports on the role of environmental factors in the male reproductive system and cancer development. We analyzed environmental pollution-related studies and disorders of mechanisms responsible for defense against the impact of xenobiotics on prostate cancer. We focused on polymorphisms that, when exposed to environmental stressors, might exacerbate an organism’s defense mechanisms against the effects of xenobiotics. It is well known that environmental factors, such as toxic heavy metal pollution, xenobiotic exposure, and undue and differentiated stressors, affect the human reproductive system. There were many studies suggesting an association between these factors and prostate cancer development, but there are still no unambiguous or conclusive results. Investigations of specific marker changes that occur in response to varied environmental stressors are also critical to mutual relations. They focus on the influence of chemical element destabilization and heavy metal pollution on organisms and the environment. Simultaneously, antioxidant enzymatic mechanisms in conditions of anthropogenic impact and the influence of polymorphisms in genes involved in genetic material damage under stress conditions were also studied. This review aims to provide essential data suggesting the role of environmental factors in the initiation and development of carcinogenic processes in the male reproductive system based on prostate cancer cases. It further clarifies this field’s current needs and research directions. It is possible to conclude that there is a relationship between the studied polymorphisms and antioxidant mechanisms, lipoperoxidation, and trace element concentrations in the blood of men with prostate cancer. The results indicate the need to consider environmental factors as necessary in assessing the risks resulting from exposure to oxidative stress in prostate cancer patients. Available data suggest the existence of interactions between exposure to environmental stressors and increased susceptibility to cancers, including male reproductive system cancers. Differentiated chemical elements introduced into the body may play a significant role. Individuals with cancer have a disturbed antioxidant enzyme status, which could be a basis for decreased defense against carcinogenic factors or the effect of disturbed body balance caused by the carcinogenic process. In turn, studies of repair gene polymorphism may indicate disorders of proteins needed for the organism’s defense against xenobiotics. The analysis presented provides data for conclusive population-based studies of the impact of environmental factors on the carcinogenic process in the male reproductive system. This review provides a basis for constructing current needs and the research direction in the discussed field of knowledge. This will allow for a precise study of the explanation of possible multilateral interactions between exposure to varied environmental stressors and the increased incidence of male reproductive system cancer at present.

## 1. Introduction

Due to the increasing number of disorders affecting the human reproductive system, numerous studies are currently underway to explain the underlying causes of this social problem. Much research has been devoted to analyzing the hypothesis that environmental factors [1,2,3,4,5,6,7,8,9] influence human reproductive organs. The direct impact of xenobiotics and the genetic background in which these factors exert their effects are considered. Aitken et al. (2006) [10] report a link between environmental xenobiotics and increased male infertility in Australia. Myrup et al.’s (2008) [11] study, analyzing descendants of Danish immigrants, also confirms an increase in cancer incidence in subsequent generations living in this industrialized country. Recently, there has also been an increase in research examining possible environmental factors involved in the pathogenesis of prostate cancer. Goyer et al. (2004) [12] emphasize the complexity of prostate cancer etiology, which includes factors such as age, ethnicity, occupation, lifestyle, and residential environment, and considers the interaction between genetic background and the environment. Prostate cancer research focuses, among other things, on the environmental impact of various elements. Environmental stress, broadly defined, is also studied by determining the activity of antioxidant mechanisms [12,13]. Recent studies also focus on gene polymorphisms that may be responsible for men’s increased susceptibility to prostate cancer [14,15,16,17].

Prostate cancer is considered the second most common solid-organ malignant tumor worldwide, with a global age-standardized incidence rate of approximately 30.7 cases per 100,000 males, and the age-standardized mortality rate worldwide is 7.7 cases per 100,000 people [18]. Early-stage prostate cancer has no apparent symptoms. However, the late stage may cause disabilities such as difficulty urinating and bone pain. The primary type of prostate cancer is adenocarcinoma (95%), followed by small cell carcinoma, which has a high metastatic rate and intertumoral heterogeneity. While high-frequency gene fusions caused by multi-genomic variations may represent one of the pathogenesis mechanisms of prostate cancer, chronic inflammation can also mediate alterations in inflammatory response regulation and gene expression profiles via diverse signaling pathways [18]. High expression of inflammatory factors such as tumor necrosis factor, interleukin IL-6, IL-18, or nuclear factor Kappa B may correlate with prostate carcinogenesis. Finally, the traditional treatment techniques for prostate cancer encompass radical surgery and endocrine therapy (burdened with possible side effects) [18].

Additionally, according to Global Cancer Statistics 2022 (GLOBOCAN 2022) estimates, prostate carcinoma ranked fourth in global incidence, with an estimated 1,467,854 new cases indicated in 2022, accounting for 7.34% of all new cancer cases that year [19]. Obviously, prostate cancer incidence differs in various populations since race and ethnicity constitute important risk factors of the disease. For instance, in the United States, the American Cancer Society reported a 4.5% annual increase in regional and distant-stage prostate cancer between 2011 and 2019. However, among Black males (the population with the highest prostate cancer incidence and mortality rates), there was 5% per year increment, on average, in the incidence of distant-stage disease between 2012 and 2018 [20]. Xue et al. (2025) [21] note that the current intensification of global aging is increasing the health and economic burdens of urological cancers. In China, from 1990 to 2021, the incidence and prevalence of these cancers increased, resulting in 266,887 new cases and 159,506,067 cases in 2021. Interestingly, regionally and provincially, provinces with the highest gross domestic product per capita have the highest burden of prostate cancer.

Furthermore, prostate cancer also constitutes the most burdensome subcategory among the population aged 55+ [21]. According to Zhang et al. (2023) [22], of all prostate cancer deaths and disability-adjusted life-years (DALYs) globally in 2019, 6% and 6.6% were attributable to smoking. Subsequently, this factor contributed to 29,298 deaths and 571,590 DALYs in the mentioned year. What is more, the number of smoking-related deaths and DALYs presented an upward trend, increasing by half from 1990 to 2019. For geographical regions, Western Europe and East Asia constituted the high-risk areas of prostate cancer deaths and DALYs attributable to smoking, among which China and the United States were the countries with the heaviest burden [22]. Finally, male reproductive system cancers may be considered an alarming problem also among adolescent and young adult males (AYAMs, aged 15–49 years). In 2021, a total of 94,229 new cases of male reproductive system cancers were reported globally in the mentioned group. Additionally, in recent years, the incidence of prostate cancer in this population has risen [23].

Immunotherapy appears to be highly effective, although specific immunosuppressive agents may also exhibit ototoxicity [18]. So far, the associations of cryptorchidism, physical trauma, testicular atrophy, and exposure to sexually transmitted diseases in the etiology of prostate cancer have been confirmed. Still, the role of environmental and genetic factors is also considered [24]. Due to the increasing number of male reproductive system disorders, such as infertility, cancers, or other diseases, many researchers try to define the causes of this medical and social problem. Many studies analyze the hypotheses of the significant environmental factors’ impact upon male genitals [10,11]. The aspects of direct xenobiotic interactions and the genetic background in which these factors interact are often the main focus of research. The validity of those studies is confirmed by reports of unresolved, concrete impacts of chemical elements introduced into the body in various bioactive forms, both in polluted air and water, and through the food chain in natural environments, as well as during dietary supplementation [12,25,26,27]. Information on the unsettled antioxidant enzyme status in patients with cancer also does not provide a definitive answer. Similarly, in the case of repair gene polymorphisms, which are responsible for impaired synthesis and metabolism of proteins essential for defending the body against xenobiotics, we have a vague message [13,15,16].

Environmental factors (excessive heavy metal pollution, xenohormones, and excessive stress) are believed to affect the human reproductive system. Aitken et al. (2006) [10] indicate an association between environmental xenobiotics and increased infertility among men in Australia. They also emphasize the increased incidence of testicular cancer in this country. Likewise, Myrup’s (2008) [11] study of Danish immigrant descendants confirms an increase in cancer incidence in subsequent generations living in industrial regions. Recently, research has examined the rise in studies estimating potential environmental factors involved in prostate cancer pathogenesis [12,15,16,27]. Simultaneously, Goyer et al. (2004) [12] pay attention to the complexity of prostate cancer, including age, ethnicity, occupation, lifestyle, and living environment.

Furthermore, they have stated that the interaction between genetic background and environment cannot be ignored. Studies of prostate cancer focused on the interactions of various chemical elements and environmental influences. Therefore, extensive analyses of the roles of cadmium, zinc, and other elements have been conducted [12,25,26,27].

Understanding the impact of environmental stressors focuses on determining the activity of enzymatic and nonenzymatic antioxidant mechanisms. The last reports also concentrate on the gene polymorphisms, which could be responsible for increased male susceptibility to prostate cancer. These polymorphisms are located in genes involved in the repair of damage induced by differentiated environmental stressors [13,15,16]. Similar research is conducted in groups among patients with testicular cancer, whose origin is also the aim of many studies [24,28,29,30]. The studies so far confirmed an association with cryptorchidism, physical injuries, testicular atrophy, and exposure to sexually transmitted diseases in prostate cancer etiology; however, scientists also consider the role of environmental and genetic factors [24].

The search strategy of this review was based on the importance of the topics discussed, the significance of the relationships between individual causal factors, and the innovativeness of the interdependencies presented. To ensure transparency and scientific accuracy, we searched the PubMed, NCBI, Springer, and Elsevier databases for information on various aspects of prostate cancer (multiaspectual analysis). We considered the publication time range based on the importance of the materials. Therefore, both recent papers and those relevant to the aspects discussed previously were considered important. We focused primarily on reports from the last 25 years as the inclusion criterion. We also included some older articles that we found particularly valuable for the topic. In our search, we obviously focused on humans, but some reports concerning animal models also appeared valuable for our analysis. We excluded only articles that were evidently unrelated to our main topic or contained inconsistent, repetitive, or outdated data. The inclusion/exclusion criteria for the analyzed publications were based on whether they met the above conditions, which were important to us. Our review is primarily narrative; therefore, when selecting the literature, we were guided by the importance of the discussed aspects, their innovativeness, and the range of factors considered. In the paragraphs on the role of gene polymorphisms in prostate cancer, we naturally began with well-established genes (*BRCA1*, *GSTP1*). Still, we did not miss any additional controversial polymorphisms (such as those in the *p53* gene) or those discussed in the most recent articles (in the *XRCC1* gene). This approach broadens and deepens the overall analysis.

## 2. The Importance of the Problem

This review aggregates the latest reports of the role of environmental factors in the male reproductive system and cancer development. This review examines environmental pollution studies and disorders of mechanisms responsible for defense against the impact of xenobiotics on prostate cancer occurrence. Moreover, our review focused on polymorphisms that, when exposed to environmental stressors, might exacerbate an organism’s defense mechanisms against the effects of xenobiotics. 

It is well known that environmental factors, such as toxic heavy metal pollution, xenobiotic exposure, and undue and differentiated stressors, affect the human reproductive system. There were many studies suggesting an association between these factors and prostate cancer development, but there are still no unambiguous conclusive results. Investigations of specific marker changes that occur in response to varied environmental stressors are also critical to mutual relations. They focus on the influence of chemical element destabilization and heavy metal pollution on organisms and the environment. Simultaneously, antioxidant enzymatic mechanisms under conditions of anthropogenic impact and the influence of polymorphisms in genes involved in the repair of genetic material damage under stress were also studied.

The novelty of this review is to provide essential data suggesting the role of environmental factors in the initiation and development of carcinogenic processes in the male reproductive system, based on prostate cancer. It further clarifies this field’s current needs and research directions. It is possible to conclude that there is a relationship between the studied polymorphisms and antioxidant mechanisms, lipoperoxidation, and trace element concentrations in the blood of men with prostate cancer. The results indicate the need to consider environmental factors as necessary in assessing the risks resulting from exposure to oxidative stress in prostate cancer patients. This review provides a basis for constructing current needs and the research direction in the discussed field of knowledge. This will allow for a precise study of the explanation of possible multilateral interactions between exposure to varied environmental stressors and the increased incidence of male reproductive system cancers at present.

This review analyzes polymorphisms as indicators of genetic susceptibility to environmental stressors; antioxidant enzyme activity to determine the role of detoxification mechanisms; concentrations of chemical elements relevant to assessing the level of environmental toxins (resulting from tobacco smoking and occupational exposure) in the bodies of prostate cancer patients; and malondialdehyde concentration, which allows for the assessment of lipoperoxidation in prostate cancer patients. Our review established the associations of specific markers, examining the relationships between polymorphisms, antioxidant enzyme activity, trace element concentrations, and the intensity of lipoperoxidation. This paper analyzed the frequencies of polymorphic variants, including glutathione S-transferases (GSTs) *GSTM1*, *GSTT1*, and *GSTP1*, antioxidant defense activity, and trace element concentrations in patients with prostate cancer. This review examined differences in exposure to environmental factors (lifestyle, smoking, alcohol consumption, diet, occupational exposure) and systemic factors (disease, genetic predisposition, family history of cancer) in the population of men with prostate cancer and in the control group. This review article considered whether detoxification mechanisms might be associated with the occurrence of the disease in individuals exposed to environmental stress, and whether the concentrations of chemical elements might differ in prostate cancer patients compared to healthy controls. This paper analyzed the associations of the studied markers, the relationships between the levels of chemical elements, polymorphisms, antioxidant enzyme activity, and the intensity of lipoperoxidation.

Our review assesses whether the concentration of chemical elements depends on *GST* polymorphisms and the body’s detoxification capacity. Correlations between antioxidant enzymes and malondialdehyde will enable the determination of men’s exposure to oxidative stress under conditions of limited or normal xenobiotic biotransformation capacity, as determined by molecular testing. This analysis provides medically relevant data that determines whether the concentration of chemical elements may differ in prostate cancer patients compared to healthy controls, whether complex detoxification mechanisms may be associated with the occurrence of the disease in individuals exposed to environmental carcinogens, and whether trace element levels may be related to polymorphisms of detoxification genes and the functioning of defense mechanisms. In summary, this analysis and correlation results suggest an association among polymorphisms, antioxidant mechanisms, lipid peroxidation, and the concentrations of certain chemical elements in blood collected from men with prostate cancer. This review highlights the need to consider these markers as key factors in further assessing the risk of exposure to oxidative stress.

## 3. Prostate Cancer as an Urgent Problem

Over the last three decades, the number of prostate cancer cases has increased dramatically. In 2020, 90,000 cases were recorded in Polish men, with an incidence rate of 376 × 10^−5^. Cancer is the second leading cause of death. Its incidence rate is characterized by a rapid increase in morbidity, reaching 1.96% annually in Polish men [8,9]. The most common cancer diagnosed in men in Poland is lung cancer, followed by prostate cancer (13%). Cancer incidence is closely related to age, with the highest increase occurring after the age of 60. The incidence of cancer in men in Poland is approximately 20% lower than the average for European Union countries, but mortality data are bleak. In 2020, 55,000 men died from cancer in Poland [8,9].

Prostate cancer is caused by both increasing toxic metal concentrations and certain genetic predispositions [31,32,33]. Lifestyle factors such as smoking, alcohol consumption, unhealthy diets, and exposure to highly polluted environments can contribute to increased cancer incidence. Residents of large cities, industrial workers, and people exposed to contaminated water and food are exposed to chemical compounds, some of which are toxic and can induce cancer [31,32,33]. Research into cancer etiology also considers the organism’s genetic susceptibility and environmental factors. Individual differences in genetic profiles are analyzed with respect to the effective detoxification of carcinogenic substances. A person with unfavorable changes in the DNA strand encoding defense mechanisms in their genome, exposed to increased exposure to toxic substances, may more easily acquire further genetic changes that can cause cancer in the future [34,35,36]. Understanding the etiology and defining the pathways of cancer development is crucial. The diversity of cancers, their locations, and multiple risk factors necessitate focusing research on a single disease entity. Such analyses can help elucidate disease pathways and enable the introduction of new therapeutic methods and preventive measures.

Prostate cancer affects the prostate gland in men, located just in front of the rectum, between the bladder and the penis. The prostate plays a crucial role in sexual function and micturition, being responsible for the secretion of semen and the initiation of urine excretion [37,38]. Prostate cancer is currently one of the most common cancers, and its mortality rate is one of the highest in men, after lung cancer. Until the 1980s, the disease’s incidence increased dramatically. After the introduction of prostate-specific antigen (PSA) screening tests, the incidence of prostate cancer did not increase, remaining stable [39,40]. The process of cancer development in prostate cancer patients begins with a single, asymptomatic tumor. This tumor can grow, infiltrating the prostate, and progress to locally advanced cancer. At this stage of the disease, symptoms associated with prostate enlargement appear. Over time, cancer cells can metastasize to other parts of the body via the blood and lymphatic vessels. These migrating cancer cells often cause death in prostate cancer patients [41]. Early detection of cancer determines the possibility of effective treatment, as well as the diagnosis of a less malignant tumor. To determine the tumor stage, a biopsy is performed and, based on microscopic evaluation, the tumor is classified into one of the Gleason grades; higher scores indicate a more aggressive tumor and a poorer prognosis [40,42]. The choice of the most appropriate therapy is based on determining the cancer stage at diagnosis [42]. Treatment methods include radical prostatectomy, teleradiotherapy, or brachytherapy. The patient’s quality of life depends on the therapy used, and with the increasing choice of treatment options, concerns about recovery are decreasing [43,44]. Despite significant progress in understanding the underlying causes of the disease, such as the discovery of the role of testosterone, scientists are still striving to identify both environmental and genetic factors that increase susceptibility to the disease [15,16].

Multifaceted interactions can be expected between exposure to environmental stressors and an increased incidence of reproductive system cancers. Chemical elements introduced into the body as chemical group compounds through polluted air, water, food, and dietary supplements play a significant role in the environmental determinants of cancers of the human reproductive system. Men with prostate cancer have a disturbed antioxidant enzyme status (pro-antioxidant balance), which may be the primary cause of their reduced defense against carcinogenic factors or may be a consequence of disturbed body homeostasis resulting from the neoplastic process. Analysis of repair gene polymorphisms indicates disturbances in the synthesis and metabolism of proteins essential for the body’s defense against xenobiotics. Further studies should provide medically relevant data to determine how environmental factors influence the initiation and development of carcinogenic processes in the human reproductive system [45].

There are various possible therapies for prostate cancer. Radical prostatectomy involves removing the entire prostate and surrounding tissues. Non-surgical options encompass radiation therapy, hormone therapy, chemotherapy, and immunotherapy. Hormone therapy involves androgen deprivation therapy and anti-androgens like enzalutamide to limit testosterone, which stimulates cancer growth. Radiation therapy options, such as conformal radiation therapy, intensity-modulated radiation therapy, and proton beam radiation, concentrate on precise cancer targeting [45].

**Figure 1:**
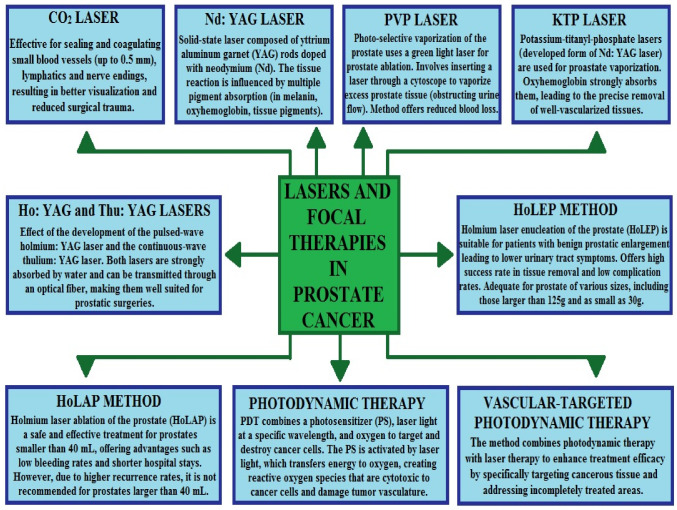
Lasers and focal therapies available for prostate cancer treatment (based on Naserghandi et al. (2025) [45]). The arrows in the figure refer to the types of therapies available for prostate cancer treatment.

Furthermore, minimally invasive ablative therapies, such as focal laser ablation and high-frequency ultrasound ablation, constitute another option for localized prostate cancer. These therapies are applied to destroy cancerous tissue while preserving healthy prostate tissue effectively. In this context, focal therapy is widely explored, as it results in fewer severe and overall side effects than surgery and radiation. Focal treatment targets only the tumor within the prostate, helping to preserve sexual function [45]. Diverse focal therapy methods include cryotherapy, high-intensity focused ultrasound, irreversible electroporation, focal brachytherapy, radiofrequency ablation, photodynamic therapy, and laser ablation. Focal laser ablation is particularly effective for treating low-to-intermediate-risk prostate cancer, giving promising results in tumor eradication while preserving the quality of life and reducing side effects such as urinary incontinence compared to more invasive treatments. Laser ablation techniques are described in more detail in Figure 1 [45]. Therefore, many possible therapies create the opportunity to choose the optimal treatment for a particular patient. It also guarantees certain advantages, such as reduced blood loss, lower complication rates, and shorter hospital stays. Thus, lasers and focal therapies constitute a valuable alternative to such radical techniques as prostatectomy (Figure 1).

On the other hand, Rai (2025) [46] mentions another possible type of therapy, namely anti-tumor vaccines. The presence of tumor-associated antigens in prostate cancer makes it adequate for anti-tumor vaccines that elicit an adaptive immune response via antigen presentation. Common vaccines for prostate cancer encompass cell-based vaccines (dendritic cell or tumor cell), vector-based vaccines, DNA/mRNA-based vaccines, and antigen- or peptide-based vaccines. DNA- and mRNA-based vaccines, consisting of plasmid DNA and mRNA, elicit an immune response. In contrast, vector-based vaccines include vectors derived from oncolytic viruses or bacterial pathogens, triggering a specific immune response [46].

## 4. Prostate Cancer Epidemiology

The etiology of prostate cancer, the second leading cause of cancer death in men, is still unclear. Two hundred thousand men die from it each year, while 190,000 new cases occur in the United States alone [15,16]. Despite significant progress in understanding the underlying causes of the disease, such as the discovery of the role of testosterone, scientists are still striving to identify both environmental and genetic factors that increase susceptibility to the disease. The above studies are, for now, a preliminary analysis of the causes of prostate cancer, the origin of which should be the subject of extensive research to identify specific factors clearly. Testicular cancer is an equally important cancer affecting men, with its incidence increasing in most countries in recent decades. Although its incidence is much lower than prostate cancer, accounting for 1–2% of all male cancers, its occurrence in young men, among whom it is the most common cancer, is cause for concern [30]. Furthermore, possible environmental factors among its causes have been implicated, which merits further analysis. The above studies are, to date, a preliminary analysis of the causes of prostate cancer, the basis of which should be the subject of extensive research to identify the specific factors clearly.

Prostate cancer is the second most common malignancy in men and most often appears late in life. According to Daniyal et al. (2014) [44], over 80% of cases are diagnosed after age 65, and the risk of developing the disease increases after age 50 [43]. Mortality associated with the disease is low at 10%, but it is believed that many cases go undetected during a man’s lifetime [44]. The prevalence of the disease, its health consequences, and mortality have become significant health problems in the modern world [44]. Prostate cancer is one of the most frequently diagnosed cancers in the world, accounting for 7% of all cancer cases. Globally, it accounts for a tenth of all cancers in men, including 14% in men from highly developed countries and 4% in men from developing countries. Prostate cancer is also responsible for 6% of cancer deaths worldwide, with 600,000 new cases diagnosed annually [8,9,47,48].

Prostate cancer is also the second leading cause of cancer in Polish men, accounting for 14% of all reported cancer cases. In 2020, 12,000 Poles were diagnosed with prostate cancer, three times more than in the 1980s. The 1990s saw a significant increase in the number of cases diagnosed [49]. In Poland, 87% of cases occur after men’s sixth decade of life, peaking after age 75. Annual survival rates are high, reaching 89% in patients diagnosed with prostate cancer between 2020 and 2023. For the same period, the 5-year survival rate was 76%, with an upward trend [8,9]. Prostate cancer causes approximately 8% of all deaths in men in Poland, accounting for 8000 cases in 2020. Most deaths occur in patients over 70, and the number of fatal cases increases with age. Compared to Europe, Poland ranks slightly above average. In 2020, the mortality rate was 12.4 × 10^−5^ for Poland vs. 12.1 × 10^−5^ for Europe [8,9]. Detection of the disease has been increasing in recent years, driven by the increased availability of diagnostic tests, including prostate-specific antigen testing. This phenomenon also contributes to a decline in prostate cancer-related mortality [50].

It is worth noting that the COVID-19 pandemic and the unexpected restrictions imposed to limit SARS-CoV-2 transmission also caused profound transformations in healthcare systems worldwide. Subsequently, these changes resulted in a full range of cancer screenings and diagnosis gaps. There was a tendency that, regardless of the recommendations, prostate cancer screening and diagnosis programs were momentarily postponed [51]. Furthermore, a decrease in prostate-specific antigen screening would significantly decrease prostate cancer detection and exert influence on possible growth in prostate cancer-specific deaths. Thus, some of the screening losses noted in 2020 were likely due to delayed or canceled screenings. Another critical factor was a drop in patients’ willingness to undertake screening owing to the anxiety of catching COVID-19 at the hospital [51]. The progression of the pandemic undoubtedly altered healthy-looking behaviors and the availability of essential diagnostic services. The problem was severe, as so many hospitals and healthcare facilities were overwhelmed by the pandemic, with high demand for medical supplies and personal protective equipment. Therefore, it is essential to ensure that patients can be continuously screened and diagnosed for prostate cancer. However, it will take years before the overall effect of the pandemic on cancer care can be adequately considered [51].

Despite certain achievements in reducing prostate cancer burden in recent years, there is still a long way to go to achieve low disease burdens globally. Furthermore, applying the same healthcare policies in different regions and populations may not be effective. Thus, there is a need to introduce comprehensive intervention measures that prioritize high-risk groups. In this context, high-income regions such as North America and Australasia should continue to invest in prostate cancer diagnosis and treatment, attempting to minimize the disease burden. On the other hand, Sub-Saharan Africa, Western Africa, and the Caribbean need to urgently promote prostate cancer screening and treatment to curb the rising disease burden [52]. Zi et al. (2024) [53] reported incidence, prevalence, mortality, and disability-adjusted life-years (DALYs) for common urologic diseases, including benign prostatic hyperplasia (BPH), urinary tract infections (UTI), urolithiasis, bladder cancer, kidney cancer, and prostate cancer. Subsequently, researchers comprehensively assessed the global burden of urologic diseases. In the context of the age-standardized prevalence rate for prostate cancer, the parameter demonstrated a noticeable increasing trend in the period from 1990 to 2021. Furthermore, in 2021, prostate cancer exhibited a significantly higher age-standardized mortality rate when compared to other tested urologic diseases [53] (Table 1).

## 5. Risk Factors for Developing Prostate Cancer

The risk factors for prostate cancer have not yet been clearly determined, despite the recognition of well-established risk factors, such as age as the main risk factor, ethnicity, geographic origin, and family history of prostate cancer [35,42,44,48]. Environmental factors are believed to be responsible for the development of prostate cancer, but genetic factors are also considered to play a significant role [40]. Among ethnic groups, Black men are at the highest risk of developing the disease. Hispanics, on the other hand, are less likely to develop the disease than Caucasian men [43,47]. Comparing the incidence of prostate cancer in white men with that in African Americans, the latter has an incidence rate that is over 50% higher. Mortality rates are also significantly higher in this case [40].

Studies have shown that a patient whose father or brother has had prostate cancer has twice the risk of developing the disease than the general population [35]. Men with one, two, or three first-degree relatives have a two-fold, five-fold, and eleven-fold increased risk, respectively, compared to individuals with no family history of prostate cancer [47]. A Scandinavian study of twin pairs suggests that inheritance of specific predisposing genes accounts for 42% of cases, with the remaining cases attributed to environmental factors [54]. The etiology of prostate cancer includes factors to which a man was exposed at home or at work [43]. Studies have highlighted the impact of tobacco smoking, excessive alcohol consumption, a high-fat diet, sexual behavior, exposure to UV radiation, and exposure to carcinogens [35,42,44]. Occupational exposures include pollution from industrial substances and agricultural pesticides [42]. Prostate cancer, as a multifactorial disease, involves a complex interaction of environmental and genetic factors, and identifying the relevant carcinogens and genes responsible for predisposition to this cancer is an interesting aspect of further research leading to an understanding of its etiology.

Currently, microbiome research is considered a valuable tool in oncology, offering novel insights and therapeutic possibilities for the diagnosis and treatment of prostate cancer. In prostate cancer, microbiome research focuses on two domains: the prostate tissue and urine microbiomes, and the oral cavity and gastrointestinal tract microbiomes. The oral and gut microbiomes are complex microbial environments that impact host immune responses, systemic inflammation, and metabolic regulation. Gut microbiota dysbiosis is connected with cancer development, and microbial imbalance may disrupt the equilibrium between pro-tumorigenic pathogens and anti-tumor commensals. Therefore, alterations in the gut microbiome are engaged in the pathogenesis and therapeutic responsiveness of prostate cancer. These effects are mediated by mechanisms such as modulation of androgen metabolism, immune regulation, and intestinal barrier integrity [55].

On the other hand, investigations into the prostate tissue microbiome remain limited. However, the microbiome of prostate cancer tissues surely presents notable diversity and significant regional heterogeneity. These microbial communities may influence tumor initiation and progression by triggering chronic inflammation, particularly prostatitis. It is worth noting that, beyond bacterial taxa, the prostate-associated microbiome encompasses a broader spectrum of microorganisms, including fungi, viruses, and parasites [55]. Finally, the urinary microbiome may be considered a reflection of disease-related microbial shifts in prostate cancer pathogenesis. However, the reliability of urine as a potential surrogate marker for the prostate microbiome requires further validation. Among various microorganisms, *Cutibacterium acnes* is commonly identified in prostate tissue, urine, and prostatic fluid from prostate cancer patients. The bacterium elicits inflammatory responses that impact tumorigenesis, cancer progression, and remodeling of the tumor microenvironment. There are noticeable differences in microbial composition across populations of different ethnic and geographic backgrounds. Thus, dietary patterns, environmental exposures, and host genetic factors may be decisive in shaping the prostate microbiome [55].

There is another report that chronic prostate inflammation constitutes a risk factor for the development of both benign prostatic hyperplasia and prostate cancer. In this context, *Propionibacterium acnes* is considered the most prevalent microorganism in the prostate gland and is thought to be a predisposing factor for prostatic tissue inflammation [56]. Thus, *P. acnes* may be involved in cancer development by modifying the prostate extracellular environment and enhancing proinflammatory responses. Furthermore, T CD4(+), FoxP3(+) (Treg), and Th17 cells may play an essential role in regulating the response to *P. acnes* infection in the context of the mentioned prostate diseases. Finally, the association between the immune response and the presence of *P. acnes* in prostate tissue indicates that the bacterium contributes to the escalation of inflammatory processes [56].

**Figure 2:**
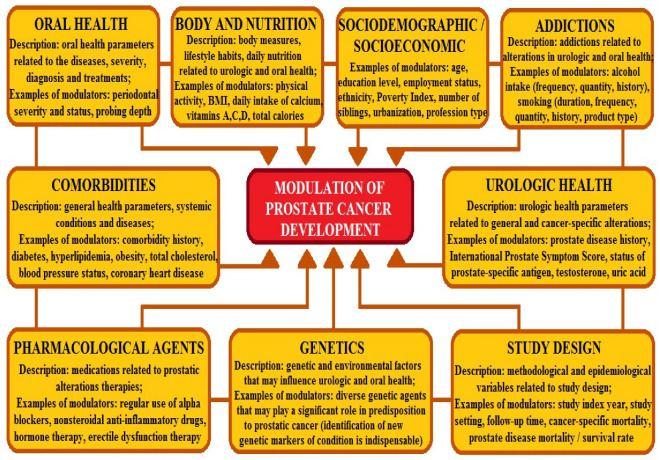
Diverse modulators of prostate cancer development (based on Peinado et al. (2025) [57]). The arrows in the figure refer to the diverse modulators of prostate cancer development.

On the other hand, Treg and Th17 cells can trigger prostate disease as a consequence of *P. acnes* infection. Subsequently, the balance between Treg and Th17 cells in patients with benign prostatic hyperplasia and prostate cancer may be a significant implication for clinicians seeking new prognostic markers and more targeted therapeutic approaches [56]. Peinado et al. (2025) [57] focused on periodontitis, an inflammatory disease that may modulate systemic conditions and influence prostatic alterations. Generally, they noted a significant connection between periodontitis and prostate cancer, with a higher risk in patients with periodontal disease. Additionally, a team underscores other confounding agents that may be engaged during the condition or considered potential risk factors [57]; see Figure 2.

## 6. The Impact of Chemical Elements on the Cancer of the Male Reproductive System

The carcinogenic impact of chemical elements and their influence on neoplastic lesions in the prostate are essential from the viewpoint of medicine and seminology. Excessive calcium intake has been studied, suggesting a possible link to prostate cancer risk [27,58]. Chemical carcinogens are divided into genotoxic and epigenetic ones. Genotoxic compounds interact directly with DNA by binding to cellular nucleic acids, causing structural and functional changes, or turning off efficient repair systems. Epigenetic carcinogens do not directly affect DNA; their effects may include cytotoxicity, tissue damage, altered immune and hormonal activity, disruption of DNA synthesis, and ROS production. Their influence is associated with further stages of the cancer process, through their effects on hereditary cells or those previously exposed to genotoxic chemical carcinogens [32,59]. Biomonitoring of elements in the blood of cancer patients can help delineate mechanisms within the patient’s body and establish relationships with other markers [60,61].

The carcinogenic effects of chemical elements and their interactions on prostate cancer are essential from a medical perspective. Numerous studies have explicitly focused on the impact of cadmium and its compounds. Cadmium, a known human carcinogen that increases the risk of lung cancer, among other things, is suspected of contributing to the etiology of prostate cancer. Despite positive results in rat tests, previous results have not provided conclusive information [10,12]. Animal studies have shown an unusual correlation in prostate tumor development, with changes occurring only in rodents treated with doses of the element below the threshold for significant testicular toxicity. This may be due to the crucial importance of androgen production by the testes, which is critical to maintaining prostate cancer. With significant toxicity, androgen secretion by the testes may be impaired, resulting in changes in the testes themselves without sustaining changes in the prostate gland [12]. In studies based on human populations, Potts et al. (1965) [62] and Elinder et al. (1985) [63] demonstrated significant associations between occupational exposure to cadmium and prostate cancer.

Cadmium is considered a carcinogen, and exposure to its compounds has been associated with the development of numerous cancers, including prostate cancer. Cadmium is suspected to play a significant role in prostate cancer etiology. The previous results did not provide unambiguous information, despite the positive results in rat tests [12]. Studies with animals show unusual dependence of prostate tumor formation, in which lesions occur only in rodents treated with doses of the element below the threshold of significant toxicity in the testes. It could be caused by the essential importance of androgen production by the testes, which is substantial in prostate cancer maintenance. Androgen secretion by the testes may be disturbed in high toxicity and results in lesions in the testes, without causing lesions in the prostate [12]. Tests on rats conducted by Białkowski et al. (1999) [64] indicate a cadmium contribution in promutagenic 8-oxo-2′-deoxyguanosine (8-OHdG) induction, through 8-oxo-dGTPase inhibition. Thus, Cd may be associated with testicular neoplastic lesions [10,12].

The unclear mechanism of action of cadmium is the source of research aimed at determining its role in carcinogenesis [65,66,67]. The International Agency for Research on Cancer (1993) classified cadmium as a human carcinogen [65,66,67,68]. Long-term exposure to cadmium is assessed by determining cadmium concentration in urine [65]. One of the main biological effects of cadmium is its association with cancer development. Research indicates a role for this metal in the etiology of kidney, liver, blood, bladder, and stomach cancers. There is evidence suggesting a link between cadmium and the induction of prostate, pancreatic, and breast cancers [67]. The mechanism of cadmium-induced carcinogenesis remains incompletely understood. The regulation of mitogenic signaling, disruption of DNA repair pathways, acquisition of resistance to apoptosis, and substitution of cadmium for zinc in transcriptional regulatory proteins may be necessary [65].

The effect of zinc supplementation, an element essential for the proper functioning of the male reproductive system, has also been studied in prostate cancer. Leitzmann et al. (2003) [69] report an increased incidence of prostate cancer in men taking high doses of zinc. A possible hypothesis in this case is that excess zinc, by preventing testicular toxicity, maintains testosterone production, which promotes the development of prostate cancer [69]; however, the negative Zn impact when imbalanced should be considered. Therefore, knowledge about the metabolism of this element and its impact on life processes continues to expand [70,71,72,73,74,75]. Zn accumulates in muscles and bones; larger amounts are found in the prostate, liver, gastrointestinal tract, kidneys, lungs, and brain. Zn is transported throughout the body via serum proteins (albumin, α-microglobulin, transferrin) [74]. Unlike other transition metal ions (Cu, Fe), it does not induce oxidative damage during metabolic processes [70,76,77]. Despite its many vital functions in the body, both deficiency and excess of Zn can cause adverse health effects. Excess Zn may be a factor in the development of prostate cancer. A promising biomarker of zinc content in the body has not been established; plasma zinc concentration is considered one possible mechanism [70,74]. A potential mechanism of zinc toxicity may involve the activation of other elements (Cd, Ni, As). The concept of reduced Cu and Fe absorption due to high Zn supplementation is also under consideration. Additionally, the mechanisms underlying the possible carcinogenicity of zinc at high doses remain unclear. Finally, zinc is considered essential for maintaining prostate health because it helps prevent the growth of malignant cells [70,74].

Another essential factor encompasses relationships between heavy metals and other potentially toxic chemical element concentrations in plasma and urine of patients with benign prostatic hyperplasia, precancerous lesions, and prostate cancer [78]. Thus, in addition to evaluating key clinical parameters such as age, total PSA levels, monocyte/lymphocyte ratio, and hemoglobin concentrations, it may be essential to determine whether specific heavy metals contribute to prostate cancer progression or even serve as biomarkers for early diagnosis. Coradduzza et al. (2025) [78] identified significant differences in plasma vanadium and antimony concentrations, suggesting a possible role in the pathophysiology of prostate disease. Researchers revealed that lower plasma antimony concentrations are connected with an increased risk of prostate cancer. On the other hand, plasma vanadium concentrations are significantly higher in the precancerous lesions group.

Studies highlighted the potential of vanadium and copper as possible biomarkers for prostate health. Finally, researchers noted a significant association between urine lead concentration and prostate cancer [78]. Undoubtedly, toxic exposures to heavy metals like arsenic and cadmium, either through inhalation and/or ingestion, may be connected with prostate cancer risk and mortality. These toxic metals also influence cellular proliferation, differentiation, apoptosis, and/or angiogenesis. They may also inhibit DNA repair and trigger oxidative stress in cell-based models. Lead generally exhibits cell toxicity and is engaged in gene mutations. Finally, mercury may trigger chromosomal aberrations [79]. In this context, Wu et al. (2021) [79] conducted logistic regression analysis to examine the association between heavy metal levels and elevated serum prostate-specific antigen (PSA) levels, a marker used for prostate cancer screening. In the group of 5477 men, 7% had elevated PSA, and participants with elevated PSA levels also showed statistically significantly higher levels of blood cadmium and blood lead compared to men with a normal PSA level, with black men having higher levels. It is well established in the literature that men with elevated serum PSA levels determined by age and race are considered at higher risk for prostate cancer. Moreover, acute and chronic prostatic inflammation is widely regarded as connected with elevated serum PSA levels [79]. The effects of lead have been widely studied, including its association with the induction of genetic mutations. Particular attention is paid to occupational exposure to lead and the possible consequences of long-term exposure to its compounds [80]. Lead is a probable carcinogen [80,81,82]. The mechanisms of carcinogenicity are not yet understood, and disturbances in nuclear DNA synthesis and repair are suggested as possible processes. The types of cancers associated with high lead doses have not been clearly defined, with lung, stomach, and gliomas being considered potential candidates [80,82].

WHO (2009, 2019, 2024) [5,6,7,8,9] has recognized 19 trace elements essential for health, including arsenic, cadmium, nickel, and zinc [83]. Exceeding the concentrations of these elements and others, including those required by the human body, can result in high toxicity. Deficiencies of essential trace elements can also disrupt the physiological processes in which they participate [32,60,83,84,85,86,87,88]. Chemical substances containing trace elements affect the human body, depending on their physicochemical properties, age, and duration of exposure [32,83]. Environmental factors, including toxic trace elements, influence prostate cancer development. The mechanisms by which elements trigger the initiation of the neoplastic process remain poorly understood.

Iron plays a significant role in prostate cancer, and increasing attention is paid to the relationship between this element’s metabolism and carcinogenesis [89]. As an essential trace element, it participates in several physiological functions. Iron in two oxidation states (Fe^2+^, Fe^3+^) can participate in various reactions as an electron donor and acceptor [71,89,90]. Unbound iron is toxic, so the body has developed effective defense mechanisms against free iron ions. It is captured by apotransferrin and, through processes facilitated by ceruloplasmin and transferrin, is transported into the cell, where it is stored by ferritin and hemosiderin. These proteins do not accumulate unbound iron in the body in harmful amounts [90]. Free iron initiates reactions that lead to the formation of ROS, which are destructive to proteins, lipids, and cellular DNA. Changes to nucleic acids are particularly harmful because they are closely linked to carcinogenesis. Iron participates in the Fenton reaction, which produces a highly reactive hydroxyl radical. This radical attacks DNA, leading to the formation of 8-oxo-2′-deoxyguanosine. Such a change introduced in a nucleic acid strand can lead to point mutations, thus increasing the risk of carcinogenesis [90,91]. Efficient antioxidant defense mechanisms, including iron metabolism, protect the body from oxidative stress, which can lead to several diseases, including cancer.

Proper copper homeostasis is essential for maintaining an appropriate metabolism level [92,93,94,95]. Copper-binding proteins are responsible for oxygen and electron transport and are catalysts of oxidation and reduction [76]. Copper is also involved in gene expression [96]. While it is an essential trace element, excess copper has toxic effects, for example, on the liver, bones, immune system, and nervous system [93]. Cu ions also form free radicals, possibly contributing to an increased mutation rate. High copper levels have been reported in the blood plasma of patients with lung cancer, gastrointestinal cancer, sarcomas, and leukemia [76]. Available studies have not identified the mechanism underlying changes in copper concentration in cancer, nor have they determined whether this is a primary phenomenon or a consequence of carcinogenesis. Cu metabolism is vital in human physiology because, as an essential element, excess copper can cause dysfunctional health issues.

Chromium is an essential trace element for humans, yet hazardous to health [97]. This element has been repeatedly linked to an increased risk of cancers, such as lung cancer [98]. It may have an affinity for erythrocytes or plasma proteins, depending on its oxidation state. This element does not accumulate in the body and is excreted primarily in urine [99,100,101]. Chromium in excess is toxic, even leading to death. Chromium is mutagenic, while Cr^3+^ is more potent in its effects on DNA. However, Cr^6+^ has been recognized as an occupational carcinogen, whereas Cr^3+^ has not been classified as a carcinogen due to insufficient evidence [100]. The role of chromium in metabolism has not been thoroughly studied. This element affects several diseases, and its supplementation is required in cases of impaired glucose tolerance. On the other hand, high doses of Cr have toxic effects, and occupational exposure to chromium has been associated with an increased risk of cancer. At the same time, environmental exposure to Cr has not been associated with carcinogenic effects and poses no danger to the general population, apart from sensitization [99,100,102].

Exposure to nickel compounds may increase susceptibility to various cancers [103]. Nickel acts as a cofactor in intestinal iron absorption and is an essential trace element required by microorganisms inhabiting the human gut [103,104]. Furthermore, nickel has been classified as a carcinogen by the International Agency for Research on Cancer. This is evidenced by studies using animal models and by studies of workers occupationally exposed to this agent [67,102]. High nickel concentrations have been found in individuals exposed to inhalation, and studies involving steelworkers, refinery workers, and nickel miners have shown an increased susceptibility to cancer in these groups [104]. Depending on the route of absorption, nickel can have genotoxic, hematotoxic, immunotoxic, and neurotoxic effects. It can impair fertility, induce respiratory diseases, and support carcinogenesis. As a potential carcinogen, nickel compounds can induce neoplastic transformation of cells. Genetic and/or epigenetic factors may influence nickel’s carcinogenicity. For example, nickel’s epigenetic effects are caused by the production of free radicals. Nickel can bind to DNA repair enzymes and generate reactive oxygen species, leading to glutathione depletion, which can result in changes in nucleic acids, resulting in mutations in active genes, a critical point in carcinogenesis [103]. Many studies indicate the role of nickel in increasing susceptibility to cancer (laryngeal sarcoma, kidney, prostate, and stomach cancer). However, the data are inconclusive and require further research [67]. The mechanisms of nickel toxicity have not been clearly defined and are still the subject of study [67].

Exposure to arsenic compounds is associated with an increased risk of prostate cancer, although the precise mechanisms of arsenic carcinogenicity have not been fully understood. An interesting aspect of arsenic effects may be the determination of the contribution of gene polymorphisms to individual susceptibility to arsenic exposure [105]. Based on epidemiological studies, the International Agency for Research on Cancer (IARC 1993) has recognized arsenic and its compounds as carcinogens, classifying them as group 1 human carcinogens [106,107,108]. Cancers associated with arsenic toxicity include skin, lung, and bladder cancer [108,109]. The mechanisms underlying arsenic-induced toxicity and carcinogenesis have not been elucidated. Attention is drawn to the possibility of causing chromosomal aberrations, inducing oxidative stress, impairment of DNA repair and methylation processes, disturbances in growth factors, cell proliferation, gene amplification, or suppression of the *p53* gene [109].

The role of mercury in increasing prostate cancer risk has not been precisely determined, despite associations with lung cancer in humans and adenocarcinomas in experimental animals. Methylmercury has been implicated as a probable human carcinogen, while the metallic and inorganic forms of the element require further study [110,111,112,113,114]. The relationship between mercury exposure and carcinogenic effects remains unclear. Mercury compounds can exert a genotoxic effect on cells. Molecular mechanisms of this process include the induction of oxidative stress, effects on microtubules, disruption of DNA repair mechanisms, and direct effects on nucleic acids [115]. From a toxicological perspective, measuring blood concentrations is a good indicator of the absorbed dose of mercury. Mercury compounds bind to hemoglobin, making peripheral blood a significant biomarker of current exposure. The toxic effects of mercury have been well understood, but the precise mechanisms of its action and its relationship to carcinogenicity remain unclear.

The prostate requires optimal levels of selenium to perform its functions, among others, in synthesizing melanoproteins that protect cellular DNA. In prostate cancer, there is a tendency for decreased levels of selenium in the prostate, probably due to a decrease in the activity of selenoproteins. On the other hand, prostate cancer can result from chronic exposure to toxic metals. It can occur via environmental contamination, food sources, or occupational exposure that compromise cellular homeostasis [116]. Thus, monitoring and controlling occupational and environmental exposures, as well as reporting increased rates of prostate cancer in communities excessively exposed to industrial metals, is so important. In this context, targeted public health campaigns may increase awareness of metal toxicity and encourage the use of exposure-reduction techniques. Another significant factor is maintaining a balance in chemical element management to lower prostate cancer risk. Cadmium, lead, nickel, mercury, and arsenic generally are linked to an elevated prostate cancer risk through triggering oxidative stress, DNA damage, inflammation processes, and disruption of cellular functions [106,116].

On the other hand, zinc and selenium decrease prostate cancer risk by supporting DNA repair and cellular health and reducing oxidative stress. Furthermore, other elements, such as manganese, are recognized as vital cofactors for several enzymatic processes integral to antioxidant defense and cellular metabolism. Ultimately, the most crucial point is that while certain trace metals can meaningfully increase prostate cancer risk, others may offer protective and beneficial activities. Therefore, managing environmental exposures and maintaining a balanced diet and undisturbed interactions between trace metals appear essential [116].

The studies by Dróżdż et al. (2012, 2013), Dróżdż-Afelt (2015, 2020, 2022, 2024), and Bombolewska et al. (2013) [117,118,119,120,121,122,123] compared trace element concentrations in the blood of patients with prostate cancer and healthy men. They found statistical significance for arsenic, chromium, copper, zinc, cadmium, and lead. They also found higher arsenic concentrations in the blood of individuals with cancer. Patients had lower concentrations of chromium, copper, zinc, as well as cadmium and lead [117,118,119,120,121,122,123], elements commonly considered carcinogenic [67,80,82]. Reports on the association of arsenic with prostate cancer suggest a link between exposure to the inorganic form of the element and the occurrence and mortality of the cancer. Evidence indicates an association between As and other types of cancer, such as skin, bladder, and lung cancer. The mechanism of arsenic’s action on prostate cells has not yet been determined. Still, human prostate epithelial cells are believed to be susceptible to neoplastic transformation after exposure to As [124]. In the studies by Dróżdż et al. (2012, 2013), Dróżdż-Afelt (2015, 2020, 2022, 2024), and Bombolewska et al. (2013) [117,118,119,120,121,122,123], the median of arsenic concentration in patients was 0.6 µg·L^−1^; in the control group, it was lower (0.2 µg·L^−1^). In the study by Goulle et al. (2005) [125], reference values for arsenic in whole blood ranged from 2.6 to 17.8 µg·L^−1^ in 100 healthy volunteers. These data are significantly higher than ours, but it should be noted that these analyses were conducted on the French population. Defining specific reference values is very difficult, as they are influenced by age, gender, environmental conditions, and ethnicity [126]. Considering the healthy control group studied by Dróżdż et al. (2012, 2013), Dróżdż-Afelt (2015, 2020, 2022, 2024), and Bombolewska et al. (2013), it can be assumed that people with prostate cancer were exposed to arsenic compounds to a greater extent [117,118,119,120,121,122,123]. Still, it is not possible to determine the duration and extent of exposure. These studies provide an exciting basis for conducting studies of long-term occupational exposure of men to arsenic to determine the risk of developing prostate cancer.

Dróżdż et al. (2012, 2013), Dróżdż-Afelt (2015, 2020, 2022, 2024), and Bombolewska et al. (2013) [117,118,119,120,121,122,123] found lower Cu, Zn, and Cr concentrations in individuals with prostate cancer. Median copper concentrations were 487.5 µg·L^−1^ in patients and 656.6 µg·L^−1^ in the control group. For zinc, these values were 4615.6 µg·L^−1^ and 5247.9 µg·L^−1^ in patients and healthy controls, respectively [117,118,119,120,121,122,123], and for chromium, the values were 6.4 µg·L^−1^ and 9.7 µg·L^−1^, respectively [117,118,119,120,121,122,123]. The zinc concentrations obtained in both groups are comparable to the reference values reported by Alimonti et al. (2005) [126]. In the case of copper, concentrations in people with prostate cancer were below the published values, whereas our obtained Cr concentrations differ significantly [126,127,128,129]. When analyzing studies of Cu and Zn concentrations in men with prostate cancer, they should be compared with the lower SOD values reported by Dróżdż et al. (2012, 2013), Dróżdż-Afelt (2015, 2020, 2022, 2024), and Bombolewska et al. (2013) [117,118,119,120,121,122,123]. Both Cu and Zn are essential trace elements that are components of enzymatic proteins, including SOD [70,76,77,96]. The low levels of these elements in patients’ blood confirm low SOD activity, which cannot function efficiently without the required components. Therefore, it is reasonable to assume that a deficiency of essential trace elements in individuals with prostate cancer may ultimately result in an imbalance in antioxidant balance. Chromium levels were also found to be lower in individuals with prostate cancer compared to the control group [117,118,119,120,121,122,123]. This element has been linked to sugar metabolism, RNA structure stabilization, and participation in protein and lipid metabolism [99,100]. No scientific reports link it to prostate cancer. In the case of the prostate cancer patients studied, reduced Cr levels in blood may be due to the body’s depletion caused by the disease or may be the result of other processes associated with the cancer that have not yet been studied.

An interesting finding is the higher concentration of Cd and Pb in the control group compared to the group of men with prostate cancer [117,118,119,120,121,122,123]. Given the correlations between chemical elements and cancer [67,80,82], higher Cd and Pb concentrations are expected in patients, especially those with greater exposure to harmful factors at work. These results indicate a significant increase in the percentage of smokers in the control group compared with patients (42% vs. 18%). Cigarette smoking is a substantial source of exposure to lead and cadmium. Higher concentrations of these elements were found in smokers [130], as also evidenced by the results of Dróżdż et al. (2012, 2013), Dróżdż-Afelt (2015, 2020, 2022, 2024), and Bombolewska et al. (2013) [117,118,119,120,121,122,123]. Analysis of trace element concentrations in patients with prostate cancer revealed differences in blood concentrations of As, Cr, Cu, Zn, Cd, and Pb. Exposure to these elements does not confirm a relationship between exposure and disease occurrence. However, it provides preliminary data for analyzing element accumulation in the prostate gland to determine its association with disease occurrence. Furthermore, combining the results with analyses of polymorphisms in detoxification genes and antioxidant enzymes provides insight into the effectiveness of these defense mechanisms against xenobiotics [117,118,119,120,121,122,123].

## 7. The Role of Enzymatic Antioxidant Mechanisms in Prostate Cancer

In the case of prostate cancer, analyses have been conducted on the functioning of mechanisms responsible for the inactivation and excretion of toxic xenobiotic substances [13,131,132]. It has long been known that oxidative stress, influenced by environmental factors, induces increased antioxidant activity, which maintains intracellular homeostasis. When this activity is disrupted, the body, lacking adequate defenses, may be exposed to damage, including changes in genetic material, which can lead to cancer development [131]. To determine the importance of antioxidant mechanisms in prostate cancer, studies were conducted on the activity of catalase CAT, superoxide dismutase SOD, and glutathione peroxidase GPx [13,131,132]. Additionally, analyses of antioxidant vitamin C and E concentrations and 8-hydroxy-2′-deoxyguanosine levels were included [132]. The studies by Battisti et al. (2011) [13] demonstrated decreased CAT activity and increased SOD in prostate cancer patients compared with healthy controls. Serum vitamins C and E were reduced in cancer patients.

Studies involving patients from Macedonia and Turkey [132] showed reduced CAT, similar to the previously cited research. Still, superoxide dismutase levels were lower in prostate cancer patients than in controls. These analyses also showed lower GPx levels in the cancer group. Studies of 8-OHdG levels, used to assess oxidative DNA damage, did not yield the expected patient increases due to an underdeveloped research process. The ambiguous data indicate an antioxidant imbalance in prostate cancer patients, supporting the hypothesis that oxidative stress influences this type of cancer [13,132]. In the case of prostate cancer, the study by Koizumi et al. (1992) [28] assessed levels of antioxidant enzymes after treatment of rats with Cd compounds. They point to the possible initiation of carcinogenesis by this element, mediated by active oxygen species. In the study above, lipoperoxidation and cellular hydrogen peroxide H_2_O_2_ production were observed, along with an increase in glutathione peroxidase activity and a reduction in glutathione reductase GR and catalase activity after Cd exposure. These data suggest a role for environmental stress in the development of prostate cancer, which provides the basis for further studies of antioxidant enzymes in men with this disease [64].

There are reports that water-soluble flavonoid derivatives, anthocyanidins, and anthocyanins, which are abundant in berries, grapes, and other dark-colored fruits, are effective at suppressing tumor cells. Furthermore, anthocyanins, such as cyanidin-3-O-glucoside, demonstrated higher activity against prostatic neoplasms than the corresponding anthocyanidins, such as delphinidin. Nevertheless, both anthocyanidins and anthocyanins have potential anti-tumor capacities. The mentioned compounds exert anti-inflammatory and antioxidative effects, may prevent mutagenesis, and stimulate beneficial processes such as autophagy or apoptosis [133]. Such fruits as cranberry, strawberry, or wild blueberry showed a high capacity to target various human cell lines (among others, CRPC (castration-resistant prostate cancer), LNCaP (androgen-independent prostate cancer), or DU145 (androgen-resistant tumoral prostatic cells). As a result, cell survival decreased while apoptosis was induced. The mechanism of action may involve down-regulation of cyclin-dependent kinase activity, enhanced expression of p21, activation of caspase-3, inhibition of matrix metalloproteinase activity, and reduced expression of phosphorylated signal transducer and activator of transcription-3 (pSTAT3) [133]. Animal studies indicate that the insoluble fraction of purple rice ethanolic extract reduced the incidence of adenocarcinoma in the lateral lobes of the prostate and prostatic intraepithelial neoplasia [133].

Much research has been devoted to the broad concept of oxidative stress in prostate cancer. It is caused by the excessive production of molecules commonly referred to as “free radicals”, more broadly referred to as reactive oxygen species (ROS). Not all ROS are free radicals; ROS contain at least one oxygen atom and, possessing one or more unpaired electrons, can readily react with other compounds, including components of body cells. ROS include the hydroxyl radical (*OH), the superoxide anion radical (O*^−^), singlet oxygen, and hydrogen peroxide (H_2_O_2_), which, as an exception, does not possess an unpaired electron. These molecules are produced during the body’s metabolic processes, such as aerobic respiration and inflammation. They are essential to cell function by participating in many processes, including hormone secretion, immune system function, muscle contraction, apoptosis, regulation of vascular tone, and removal of foreign substances from the body [134,135,136,137,138].

Djokic et al. (2022) [139] remind us that since oxidative damages during prostate carcinogenesis may contribute to modifications of macromolecules that trigger cellular malignant transformations, it is worth considering that functional single-nucleotide polymorphisms of enzymes engaged in redox homeostasis can also disrupt the pro-oxidant–antioxidant balance, resulting in accumulation of reactive oxygen species and oxidative damage. In this context, researchers focused on the roles of polymorphisms in antioxidant enzymes GPx1 (*GPX1* rs1050450) and SOD2 (*SOD2* rs4880), as well as the regulatory antioxidant protein nuclear factor erythroid 2-related factor 2 (*Nrf2* rs6721961) in shaping prostate cancer prognosis and development [139]. The team concluded that independently, carriers of at least one SOD2*C allele presented an increased risk of prostate cancer development. The effect was significantly further amplified in advanced statistical models. Additionally, when tested in combination, participants with both the SOD2*C allele and the Nrf2*C/C genotype also showed an increased risk of prostate cancer development. At the same time, the phenomenon was augmented when combined with acquired contributory factors. Researchers suggest that tested gene polymorphisms may potentially serve as risk biomarkers of prostate cancer evolution [139].

In addition, Lin et al. (2023) [140] underscore the importance of nonenzymatic antioxidants in shaping prostate-specific antigen (PSA) levels. In this context, high PSA can indicate potential prostate problems and constitute an alarming sign of prostate cancer. Therefore, researchers evaluated fourteen dietary and endogenous antioxidants connected with PSA levels (Table 2). PSA values were categorized into normal, borderline, and elevated levels. Additionally, two groups of men were included, namely middle-aged men (40–64.9 years) and older men (≥65 years) [140]. The team noted that 0.3% and 3.4% of the middle-aged and older groups, respectively, had elevated PSA levels (>10 ng·mL^−1^). Furthermore, men with higher serum albumin levels had a decreased risk of an elevated PSA level. In contrast, the impact of albumin on PSA was larger in middle-aged men than in older men (based on ORs for elevated PSA of 0.82 and 0.90, respectively; interaction *p* = 0.002). Researchers also identified a potentially beneficial effect of vitamin D on PSA levels in middle-aged men. The impact of other tested antioxidants on PSA was more ambiguous. Finally, a well-balanced diet rich in protein is recommended for maintaining prostate health [140]; see Table 2. These experiments demonstrate the significant role of enzymatic and nonenzymatic agents in counteracting prostate cancer.

Currently, attention is being paid to the possible involvement of lipoperoxidation in the development of prostate cancer [141,142]. Measurement of malondialdehyde MDA concentration is considered a reliable indicator of increased oxidative processes and the antioxidant status [143,144]. Dróżdż et al. (2012, 2013), Dróżdż-Afelt (2015, 2020, 2022, 2024), and Bombolewska et al. (2013) [117,118,119,120,121,122,123] measured the activity of GST, SOD, CAT, and MDA concentrations in patients with prostate cancer and controls. A significant difference between the study groups was observed only in SOD activity. Comparison of measurements of other biomarkers of oxidative stress did not reveal significant differences. The median GST enzyme activity was lower in the study group (0.7 U·mL^−1^ vs. 1.0 U·mL^−1^ in the control group).

Superoxide dismutase is the first line of defense against the toxic effects of peroxides, and disturbances in its function allow free radicals to attack body structures, including DNA, which can consequently lead to the development of cancerous changes [135,145]. In studies by Dróżdż et al. (2012, 2013), Dróżdż-Afelt (2015, 2020, 2022, 2024), and Bombolewska et al. (2013) [117,118,119,120,121,122,123], reduced SOD activity was observed in individuals with prostate cancer compared to controls. Similar results were obtained by Kotrikadze et al. (2008) [142], who found lower SOD activity in erythrocytes from patients with prostate cancer. Similar results were presented in a study involving Turkish and Macedonian patients, which also examined SOD activity in erythrocytes [132]. However, the survey by Battisti et al. (2011) [13] showed higher SOD activity in whole blood from patients with prostate cancer compared to controls. Similar results were obtained by Surapaneni et al. (2006) [146] in India, who examined enzyme activity in the erythrocytes of men with prostate cancer.

Limited antioxidant activity of SOD may lead to mutations [145]. The low SOD activity observed by Dróżdż et al. (2012, 2013), Dróżdż-Afelt (2015, 2020, 2022, 2024), and Bombolewska et al. (2013) [117,118,119,120,121,122,123] may be due to impaired antioxidant defense systems in patients with prostate cancer [132]. The lack of significant differences in MDA concentration between patients and controls hampers detailed interpretation of these investigations. In the study by Arsova-Sarafinovska et al. (2009) [132], a decrease in SOD activity correlated with an increase in MDA concentration. This was explained by the exhaustion of the antioxidant defense system due to severe oxidative stress. In the prostate cancer patients included in the studies by Dróżdż et al. (2012, 2013), Dróżdż-Afelt (2015, 2020, 2022, 2024), and Bombolewska et al. (2013) [117,118,119,120,121,122,123], lipoperoxidation processes were not enhanced; therefore, the decreased SOD activity may be associated either with a primary defect in prostate cancer patients or with impaired enzyme function due to disease processes. A reduction in SOD activity is not expected as a result of long-term exposure to free radicals, as analysis of MDA concentration, one of the most frequently used measures of oxidative stress, did not reveal significant differences between patients and healthy individuals [117,118,119,120,121,122,123]. Many studies on cancer indicate higher MDA concentrations in patients [13,146,147,148]. However, reduced lipid peroxidation has been demonstrated in studies of breast cancer [146]. The results obtained by Dróżdż et al. (2012, 2013), Dróżdż-Afelt (2015, 2020, 2022, 2024), and Bombolewska et al. (2013) [117,118,119,120,121,122,123] are therefore different from others, which indicate increased oxidative stress in people with cancer. Still, they confirm the fact of disturbances in the functioning of antioxidant defense in men with prostate cancer, characterized by reduced SOD activity (1.4 U·mL^−1^ in patients vs. 1.6 U·mL^−1^ in the controls).

The activities of GST and CAT did not differ significantly from those of the controls. GST activity was lower in the cancer group, but there was no statistical significance compared to the controls [117,118,119,120,121,122,123]. A similar lack of differences between the study groups was observed by Surapaneni et al. (2006) [146], who examined enzyme activity in the same material collected from patients (plasma). In turn, results from other authors on catalase activity indicate decreased enzyme activity in the prostate cancer group [13,142], suggesting enzyme depletion after long-term exposure to free radicals. In the studies by Dróżdż et al. (2012, 2013), Dróżdż-Afelt (2015, 2020, 2022, 2024), and Bombolewska et al. (2013) [117,118,119,120,121,122,123], the authors found that in the absence of indications of long-term oxidative stress by measuring the intensity of lipoperoxidation processes, the lack of significant changes in the activity of GST and CAT supports the theory of a low-intensity contribution of excessive oxidative stress to the disease process in people with prostate cancer.

The reduced SOD activity in these individuals supports the theory of antioxidant system dysfunction in patients with prostate cancer. It is believed that a weakened defense system against ROS may lead to the accumulation of free radicals, thereby exacerbating the neoplastic process [145]. In the patients studied by Dróżdż et al. (2012, 2013), Dróżdż-Afelt (2015, 2020, 2022, 2024), and Bombolewska et al. (2013) [117,118,119,120,121,122,123], the authors demonstrated no increase in lipoperoxidation and, therefore, no increase in oxidative stress; however, this is the current state of knowledge. Retrospective knowledge regarding the condition of the study subjects before the development of cancer is lacking. During the study, their pro-antioxidant balance was maintained through treatment and isolation from factors that induce oxidative stress. The possibility of free radical-induced damage other than increased lipoperoxidation or the existence of a primary defect contributing to the reduction in SOD activity should be considered [117,118,119,120,121,122,123].

## 8. Biomarkers in Prostate Cancer

Recent studies also focus on gene polymorphisms that may be responsible for increased susceptibility to prostate cancer in men [14,15,16,17]. Studies of prostate cancer have revealed the significant role of genetic factors. Several polymorphisms that increase susceptibility to this cancer have been identified, and selected mutations in the *CHECK2*, *NBS1*, and *BRCA1* genes are being analyzed as markers. Additionally, new population-specific changes are being sought that may contribute to an increased risk of prostate cancer [14,15,17]. Intensive work is underway to identify genetic polymorphisms in *GST*, one of the key enzymes that inactivate carcinogens. GSTs are an intriguing subject of analysis due to their broad substrate specificity. *GST* polymorphisms resulting in reduced or lost enzymatic activity (*GSTM1*, *GSTT1*, *GSTP1*) are being extensively studied. Reports of associations between these genetic variants and cancer suggest their involvement in the etiology of colon, breast, lung, and prostate cancer. Despite ambiguous results, changes in the human *GST* genes may be associated with the incidence of various types of cancer [149,150]. These analyses attempt to explain the etiology and processes driving disease progression. However, despite numerous attempts, specific risk factors, interactions between them, and pathways for prostate cancer development have not been identified. Furthermore, no detailed analyses have been conducted on the relationship between exposure to chemical elements and internal body conditions, including polymorphisms in genes responsible for detoxification and antioxidant mechanisms.

Finally, microRNA-based signatures are promising biomarkers for stratification, diagnosis, predisposition, and progression in prostate cancer. miRNAs also appear essential in absorption-distribution-metabolism-excretion-toxicity (ADMET) related transcripts. Thus, miRNAs may be considered as candidate prognostic indicators for prostatic oncogenesis. On the other hand, central to the xenobiotic response system is the cytochrome P450 heme enzyme system [151]. These enzymes also play a dual role in carcinogenesis as they are engaged in the bioactivation and inactivation of carcinogens and anticancer drugs.

Additionally, CYPs appear essential for activating various environmental carcinogens and oncogenic mutations. It gains significance since variations in the metabolic pathways of androgens, specifically testosterone metabolites, also exert an influence on prostate cancer susceptibility through the enzymatic activity of CYP450 members [151]. Therefore, future approaches will likely focus on more accurate use of miRNA panels for prostate cancer prediction. Polygenic integrated signatures based on variants that cannot be generalized to drug outcomes, resulting from the combined effect of polypharmacy, should also be considered, as should the utility of urinary androgen metabolomic patterns for prostate cancer sensitivity in conjunction with epigenetic and CYP450 genotyping or expression analysis [151].

The mechanisms of prostate cancer remain unclear, implicating both genetic and environmental factors. Alterations in genes encoding DNA repair pathways, which play a critical role in preventing carcinogenesis, may increase susceptibility to cancers, including prostate cancer [152,153,154]. Many studies on prostate carcinogenesis indicate the involvement of oxidative stress and reactive oxygen species in the development of neoplastic lesions. Impaired removal of these products can damage biomolecules, influencing the initiation of cell transformation. These connections prompt the study of the relationships among various antioxidants, substances that generate free radicals, and indicators of oxidative stress in cancer [155,156]. Prostate cancer is primarily associated with processes of aging, including the accumulation of DNA damage in cells across various tissues. Another recognized risk factor for prostate cancer development is an imbalance in redox homeostasis, manifested by changes in antioxidant enzyme activity and the accumulation of reactive oxygen species [157]. Certain chemical compounds may also be responsible for the development of neoplastic lesions, as their properties directly and indirectly influence the occurrence of mutations that lead to the disease. Among such carcinogenic processes is the induction of free radicals by xenobiotics. Carcinogenic elements include chromium, arsenic, nickel, and cadmium, whose compounds are widely distributed as industrial pollutants [32,158].

In the genetics of prostate cancer, attention is focused on a superfamily of phase II enzymes of xenobiotic metabolism, glutathione S-transferases, which catalyze the conjugation of chemically reactive compounds with reduced glutathione. They also play a crucial role in protecting DNA and other macromolecules from oxidative stress-induced damage. The GSTM1 enzyme is involved in the metabolism of styrene oxide, the GSTT1 enzyme participates in the detoxification of dichloromethane and ethylene oxide, and the GSTP1 enzyme is responsible for the detoxification of carcinogenic heterocyclic amines. All of these substances are carcinogens; therefore, impairment of these enzymes’ function prevents the efficient removal of toxic compounds, which may indirectly increase susceptibility to various cancers, including prostate cancer. However, the mechanisms by which *GST* polymorphisms may influence the increased risk of prostate cancer have not yet been identified [154,156,159].

Therefore, this review aims to determine the associations of gene polymorphisms, as markers of impaired xenobiotic detoxification capacity, with determinants of antioxidant defense, such as dismutases, catalases, and transferases, with lipoperoxidation, which determines the intensity of ROS production, and with trace element concentrations, which reflect exposure to environmental toxins in men diagnosed with prostate cancer. This study aims to determine whether *GSTM1*, *GSTT1*, and *GSTP1* polymorphisms modify the risk of developing prostate cancer and whether, in conjunction with other factors, these genetic variations may be determinants of specific changes in biomarkers (antioxidant and detoxification enzymes, MDA concentration, trace elements). This review is one of the first analyses of *GST* polymorphism studies in a group of Polish patients with prostate cancer, along with the correlation of the obtained data with the activity of antioxidant and detoxification enzymes, the level of lipoperoxidation, and the concentration of trace elements.

In the studies by Dróżdż et al. (2012, 2013), Dróżdż-Afelt (2015, 2020, 2022, 2024), and Bombolewska et al. (2013) [117,118,119,120,121,122,123], the material from patients diagnosed with prostate cancer and healthy men who served as controls was analyzed. The control group was over 50 years old. The mean age difference between patients and healthy controls was 8.5 years. Environmental factors were also considered and analyzed using a survey according to WHO standards [8,9]. The responses provided allowed us to characterize and compare the study participants with controls. The analysis provided information on significant differences in the prevalence of genetic diseases in the family, harmful workplace factors, and smoking rates. These factors lead to the conclusion that they may be related to the occurrence of the disease in men with prostate cancer. In the studies by Dróżdż et al. (2012, 2013), Dróżdż-Afelt (2015, 2020, 2022, 2024), and Bombolewska et al. (2013) [117,118,119,120,121,122,123], nearly half of the respondents with prostate cancer reported a history of cancer in their immediate family. The responses included various types of cancer, including prostate cancer in the respondent’s father or brother.

Because cancer is a genetic disease resulting from damage to several genes, many such changes are induced during life as sporadic mutations. Still, hereditary changes also predispose to the disease’s development, which may have occurred in the previous generation [160,161]. The risk of prostate cancer is strongly dependent on familial occurrence of the disease, indicating a 5–10% contribution of hereditary genetic defects in the overall incidence of the disease and approximately 45% of cases with early diagnosis [162,163,164]; see Dróżdż et al. (2012, 2013), Dróżdż-Afelt (2015, 2020, 2022, 2024), and Bombolewska et al. (2013) [117,118,119,120,121,122,123]. The occurrence of different types of cancer in the family has been analyzed, keeping in mind that some cancer types are associated with the same mutations (e.g., the *BRCA1* and *BRCA2* genes, which predispose to breast and ovarian cancer). Despite extensive genetic studies of prostate cancer predisposition over the past two decades, specific genes remain largely unidentified [165]. These results prompted further analysis of genes suspected to contribute to the etiology of prostate cancer, such as *GST* genes.

Many studies on occupational exposures have also been conducted in relation to prostate cancer, but their results have been inconclusive. The primary focus is on agriculture, the rubber industry, and the metallurgical industry. Exposure factors suspected of contributing to the etiology of prostate cancer include pesticides, cadmium compounds, and polycyclic aromatic hydrocarbons [166,167,168]. In the studies by Dróżdż et al. (2012, 2013), Dróżdż-Afelt (2015, 2020, 2022, 2024), and Bombolewska et al. (2013) [117,118,119,120,121,122,123], over 42% of men with prostate cancer reported high or moderate exposure to harmful factors. Among the toxic substances reported by the survey respondents were metals, chemical reagents, paints, fertilizers, car exhaust fumes, and ionizing radiation. The statistically significantly higher percentage of responses in the study group compared to the controls indicates the likelihood of prostate cancer being related to the environmental factors to which the subjects were exposed at work. This provides the basis for further analysis of element concentrations in patients, which will serve as an assessment of the risk of toxic substances [117,118,119,120,121,122,123].

A comparison of smoking rates between prostate cancer patients and controls revealed a higher percentage of smokers in the control group (including current smokers and those who had smoked within the past 10 years). This may be due to greater health care among hospitalized patients (perhaps the duration of the disease lasts many years) or their awareness of the risk of cancer based on knowledge of cases in close family members. Scientific data, which to date do not confirm a link between cigarette smoking and the occurrence of prostate cancer, are also important [168]. Survey results also showed no differences between the study groups in questions on place of residence, alcohol consumption, diet, occupation, the occurrence of lifestyle diseases, and medication use. These data demonstrate the homogeneity of the comparison groups and the absence of factors that could constitute a significant confounding factor in the interpretation of the obtained biomarker measurements [117,118,119,120,121,122,123].

## 9. Polymorphisms of Genes Involved in the Activation of Carcinogens

Research on prostate cancer has revealed the critical role of genetic factors. Several polymorphisms that increase susceptibility to this cancer have been identified, and selected mutations in the *CHECK2*, *NBS1*, and *BRCA1* genes are being analyzed as markers. Additionally, new, population-specific changes are being sought that may contribute to an increased risk of prostate cancer [14,15,17]. Intensive work is underway to identify genetic polymorphisms in glutathione S-transferase, one of the main enzymes involved in the inactivation of carcinogens. Harries et al. (1997) [29] developed a PCR assay to determine the frequency of variants in the *GSTP1* locus, which may contain adenine or guanine. Possessing a reduced form of the enzyme activity may be a risk factor for cancer, and reduced *GSTP1* expression has been found in prostate cancer.

Despite the unclear results, scientists believe that variations in the human *GST* gene may be linked to the incidence of various cancers. Mittal et al. (2011) [17] studied polymorphisms of the *p53* gene, which, among its numerous regulatory functions, also repairs DNA damage. They discovered that a variant in codon 72 of this gene causes impaired protein function, which may influence the development of various types of cancer. The study included a population of African descent and demonstrated a possible association of this *p53* polymorphism with prostate cancer. However, other studies on codon 72 variation have reported conflicting results, suggesting different associations across populations [16]. In testicular cancer, Peng et al. (1993) [169] reported that none of the patients studied had mutations in the *p53* gene. However, work is still ongoing, suggesting the possible occurrence of changes related to this cancer.

There are contradictory results on the genotype distribution of the *p53* codon 72 polymorphism. However, there are current reports that the *Arg*/*Pro* genotype and *Pro* alleles are more frequently detected in individuals with prostate cancer. The apoptosis-stimulating proteins of the *p53* family regulate the apoptosis function of the *p53* codon 72 *Arg*/*Pro* polymorphism. The apoptosis function of the p53 codon 72 *Pro* variant is also selectively inhibited by apoptosis-stimulating protein inhibitors. On the other hand, the capacity of the *p53* codon 72 *Arg* variant to trigger apoptosis results from its ability to escape the aforementioned inhibition and localize to the mitochondria. Finally, apoptosis is less frequently detected in individuals with the *p53* codon 72 *Pro*/*Pro* genotype than in those with the *Arg*/*Arg* genotype. Thus, the *Pro* allele appears to be more susceptible to cancer development [170].

While analyzing the association between cytochrome *P450 1B1* polymorphisms and prostate cancer, Tang et al. (2000) [14] noted an increased frequency of the *Leu432Val* polymorphism in cancer patients compared with healthy controls. Their finding suggests that impaired enzyme activity may contribute to carcinogenesis among Caucasians. This alteration has not been studied in patients with testicular cancer, which presents an interesting area for research. Studies are also underway on breast cancer susceptibility genes (*BRCA1*, *BRCA2*) as possible causative factors of prostate tumors. The results are also inconsistent; however, numerous studies show meaningful associations [171,172]. The survey by Kirchoff et al. (2004) [171] confirms the hypothesis that *BRCA2* deletions are associated with an increased risk of prostate cancer. The impact of *BRCA2* mutations on this type of cancer is also described by Agalliu et al. (2009) [172], who additionally demonstrated that the *BRCA1-185delAG* mutation was associated with cancers classified as high Gleason (7–10) or low Gleason (2–6). Given the association of *BRCA1* and *BRCA2* gene mutations with many types of cancer (high Gleason scores 7–10 and low Gleason scores 2–6), changes can also be sought in patients for whom larger-scale research has not been conducted. The results may provide a new understanding of the causality of male reproductive system cancers. 

Prostate cancer studies have also shown that genetic factors play an essential role. Scientists identified many polymorphisms that confer increased susceptibility to this cancer, and selected *CHECK2*, *NBS1*, and *BRCA1* gene mutations have been analyzed as markers. Attention is paid to *SRY* (high 7–10 and low 2–6 Gleason score cancers) gene mutations, which could be involved in this disease development. In addition, new directions, including population-specific changes that could underlie an increased risk of these tumors, are sought [14,15,17]. Intensive studies of GST genetic polymorphisms (in *GSTP1*) are also being conducted. It is one of the main enzymes involved in the inactivation of carcinogens. Harries et al. (1997) [29] developed PCR analysis to determine the incidence of variants at the *GSTP1* locus, where adenine or guanine could occur. The possession of a lower activity form of the enzyme may be a cancer risk factor, and in prostate cancer, decreased expression of *GSTP1* has been found [29]. In testicular cancer, the increased incidence of the lower activity allele of *GST* homozygotes has been noted. Still, it has also drawn attention to the possible association with patients’ age. Despite unclear results, it is believed that changes in the human glutathione S-transferase gene can be linked to the incidence of various cancer types (such as prostate cancer).

Recent studies by Sheehan et al. (2025) [173] concern Prostaglandin-Endoperoxide Synthases (PTGS) 1 and 2. The polymorphic genes *PTGS1* and *PTGS2* encode cyclooxygenases COX-1 and COX-2, respectively, and overexpression of these cyclooxygenases is associated with inflammation and neoplasms. Thus, researchers focused on the potential association between the single-nucleotide polymorphism*-842A* > *G* (rs10306114) of the *PTGS1* gene and *-765G* > *C* (rs20417) of the *PTGS2* gene with benign prostate hyperplasia and prostate cancer (Sheehan et al. 2025) [173]. Finally, no significant association was observed between the *A* or *G* alleles or the *AA*, *AG*, or *GG* genotypes at SNP*-842A* > *G* of the *PTGS1* gene and prostatic diseases. On the other hand, researchers identified that the *C* allele of *SNP-765G* > *C* of the *PTGS2* gene was significantly associated with an increased risk of benign prostate hyperplasia. What is more, differences in the ratios of *GG*/*GC* and *GG*/(*GC* + *CC*) genotypes suggested a possible association between the *C* allele and prostate cancer, and the combined affected (prostate cancer + benign prostate hyperplasia) group [173]. Since damaged DNA repair ability can lead to the development of prostate cancer, Deng et al. (2025) [174] focused on another polymorphism concerning the *X-ray repair cross-complementary group 1* (*XRCC1*) gene at codon 399, namely *XRCC1-Arg399Gln* (rs25487), that may alter the structure of DNA repair enzymes that are responsible for the regulation of DNA repair capacity. Researchers involved 5803 cases of prostate cancer and 5470 controls and, through meta-analysis, identified a significant association between the *XRCC1-Arg399Gln* polymorphism and the risk of prostate cancer [174]. This team explains that according to the recessive models, it was associated with an insignificantly increased prevalence of prostate cancer in the Asian population considered in the mentioned experiment (*AA* versus *AG* + *GG*:OR = 1.255, *p* = 0.507). Finally, the presence of the 399Gln variant allele reduces DNA repair activity, thereby increasing susceptibility to various cancers. However, the distribution of *XRCC1* SNPs probably differs across ethnic groups [174]. According to Goel et al. (2025) [175], low XRCC1 mRNA and protein levels in cancer cells are associated with increased cancer risk, poorer survival, greater tumor aggressiveness, and resistance to certain therapies. There is also a wide variance in XRCC1 protein expression across prostate cancer tumors. For instance, significantly lower expression tends to be noted in African American prostate cancer cases. The appearance of aggressive prostate cancer with poor clinical outcomes often accompanies it. Since XRCC1 is a DNA repair protein critical for base excision repair and single-strand break repair, the team concludes that XRCC1 deficiency may hypersensitize prostate cancer cells to multiple Poly(ADP-ribose) Polymerase (PARP) inhibitors. The mechanism involves promoting the accumulation of DNA double-strand breaks, increasing cell-cycle arrest, and inducing apoptosis. Thus, XRCC1 deficiency may be considered as a promising target for treatment with PARP inhibitors, especially for African American prostate cancer patients [175]. Mentioned recent studies show that the identification of new genetic markers of prostate cancer is a dynamic and indispensable process.

## 10. The Role of Glutathione S-Transferase (GST) Polymorphisms in Prostate Cancer

Glutathione-S-transferases are a multigene family responsible for producing phase II enzymes in xenobiotic metabolism [176,177,178]. These proteins catalyze the conjugation of glutathione to electrophilic compounds, thereby mediating the removal of endogenous and exogenous substances, including carcinogens, from the body [150]. An example of a carcinogenic substance inactivated by GST enzymes is polycyclic aromatic hydrocarbons (PAHs), components of cigarette smoke, grilled meats, and diesel fuel. GSTs also detoxify ROS and endogenous metabolites of steroid hormones [179]. Among the functions of GSTs is participation in the modulation of the induction of other enzymes, which is necessary, among others, in DNA repair processes [177]. GST enzymes are encoded by at least eight distinct loci containing one or more homodimeric or heterodimeric isoforms. The most widely described are three loci: *GSTT1*, *GSTM1*, and *GSTP1* [177,180]. These include two deletion variants of *GST* (*GSTT1*, *GSTM1*), which result in loss of enzymatic activity, and one polymorphism within the enzyme’s active site (*GSTP1*), which alters conjugation activity and substrate-specific thermostability [150,181]. GST enzymes are primarily involved in the metabolism of xenobiotics, including potential carcinogens. Therefore, they are believed to protect the body against toxic and carcinogenic compounds that enter the body through pollutants, drugs, or food additives. It has been suggested that individuals carrying loss-of-function genes or genes with altered enzymatic activity may have a limited ability to efficiently eliminate carcinogens, leading to their accumulation and, consequently, an increased risk of mutations [178,182,183]. The *GSTM1* gene encodes information for GST, a “mu” class member. This enzyme’s function includes detoxifying electrophilic compounds, including drugs, various carcinogens (such as benzopyrene, a component of cigarette smoke), environmental toxins, and oxidative stress products. This occurs through coupling with glutathione in phase II of xenobiotic metabolism [178,184,185]. Genes encoding the “mu” class are located on chromosome *1q13.3* and are highly polymorphic. These genes are thought to influence susceptibility to carcinogens and toxins, as well as the body’s response to certain drugs. Due to their presumed increased sensitivity to the carcinogenic effects of environmental toxins, they are suspected of being associated with an increased risk of certain cancers [184,186].

Inheritance of a *GSTM1* deletion, i.e., a homozygous null variant of the gene, leads to the inactivity of this form of the enzyme. The null genotype, with its reduced ability to detoxify selected carcinogens, may be associated with an increased risk of solid tumors [35,178]. The prevalence of this polymorphism in the Caucasian population has been determined to range from 13.1% to 54.5% [177]. Slovak studies report a prevalence of the *GSTM1* polymorphism of 47–58% among white Europeans [35]. The *GSTT1* gene, located on chromosome *22q11.23*, is a member of a superfamily of genes encoding proteins that catalyze the conjugation of reduced glutathione with various hydrophobic and electrophilic compounds. Its likely role in carcinogenesis has been emphasized. The function of *GSTT1* is to detoxify smaller, reactive hydrocarbons, such as ethylene oxide [178,187]. The *GSTT1* null genotype, as with the *GSTM1* null polymorphism, results in a lack of protein enzymatic activity. It is believed that, due to reduced ability to detoxify ethylene oxide metabolites, this variant may provide information on exposure to environmental or dietary factors that may cause genetic damage [178,186,188]. Deletion of the *GSTT1* gene has been associated with an increased risk of developing ovarian, bladder, and lung cancer. The prevalence of homozygous deletion of this gene has been estimated at 11.1–28.6% in the Caucasian population [177], while a Slovak study reported a prevalence of 13–25% in white Europeans [35].

The *GSTP1* gene is considered one of the key enzymes involved in the inactivation of carcinogens in cigarette smoke. Substances neutralized by *GSTP1* include benzo-(α)-pyrene epoxide diol and acrolein, a common environmental pollutant that exhibits high reactivity with body cells [29,189,190]. It has been noted that the gene encoding this transferase is inactivated by hypermethylation in the early stages of prostate carcinogenesis, and that its expression is also disrupted in samples from other cancers, suggesting a role in cancer etiology [29]. The most extensively studied *GSTP1* polymorphism, *Ile105Val*, results from an isoleucine-to-valine substitution at codon 105 and most likely alters substrate-specific thermostability and impairs conjugation activity. The *GSTP1 Val*/*Val* variant has been associated with reduced detoxification of the epoxy diols of some polycyclic aromatic hydrocarbons compared with the *GSTP1 Ile*/*Ile* variant [150,191,192]. According to the 2009 meta-analysis by Mo et al. (2009) [177], the frequency of this polymorphism, located on chromosome *11q13*, is approximately 9–12.6% in the Caucasian population for the homozygous *Val*/*Val* variant. Sivonova et al. (2009) [35], in turn, reported data for the white European population, showing a prevalence of *Ile*/*Val* heterozygotes ranging from 38% to 45.7% and *Val*/*Val* homozygotes ranging from 7% to 13% [193].

Dróżdż et al. (2012, 2013), Dróżdż-Afelt (2015, 2020, 2022, 2024), and Bombolewska et al. (2013) [117,118,119,120,121,122,123] examined the presence of genetic variants in *GSTM1*, *GSTT1*, and *GSTP1* in 66 patients diagnosed with prostate cancer at the Oncology Center in Bydgoszcz, Poland. Statistical analysis of the results revealed no significant differences in the frequency of these polymorphisms between the study groups. These authors detected *GSTM1* deletions in 47% of prostate cancer patients and 55% of control subjects. *GSTT1* deletions were detected in 17% of cases and 14% of controls. In turn, the distribution of *GSTP1 Ile*/*Ile*, *Ile*/*Val*, and *Val*/*Val* polymorphisms was 51.5%, 39%, and 9% in patients and 61%, 34%, and 5% in the controls, respectively [117,118,119,120,121,122,123].

The meta-analysis by Mo et al. (2009) [177] reported population prevalences of *GSTM1* deletions ranging from 13.1% to 54.5% and of the *GSTT1* null variant ranging from 11.1% to 28.6%. These authors reported previously published data on the *Val*/*Val* allele prevalence ranging from 9% to 12.6% in Caucasians. Also, they reported higher percentages (38.9% to 62.1%), which may be biased by including studies that reported the *Ile*/*Val* and *Val*/*Val* variants in the analysis, resulting in combined percentages. According to these data, the results of Dróżdż et al. (2012, 2013), Dróżdż-Afelt (2015, 2020, 2022, 2024), and Bombolewska et al. (2013) [117,118,119,120,121,122,123] for *GSTM1* and *GSTT1* polymorphisms are within the range of those obtained in the general population, both in cases and controls. In turn, the *GSTP1 Val*/*Val* variant observed in the patients falls within the range reported in previously cited studies of the Caucasian population. In contrast, this percentage is lower than expected in the controls in the studies by Dróżdż et al. (2012, 2013), Dróżdż-Afelt (2015, 2020, 2022, 2024), and Bombolewska et al. (2013) [117,118,119,120,121,122,123]. Similar low results for the *GSTP1 Val*/*Val* allele frequency were obtained by other authors studying Caucasians, e.g., Nam et al. (2003) [194]—5.5%, Mittal et al. (2006) [195]—4.8%, Srivastava et al. (2005) [15]—3.5%.

In a meta-analysis by Mo et al. (2009) [177], *GSTM1* deletion was found to increase the risk of prostate cancer in the general population, including Asians, African Americans, and Caucasians. No significant associations were found between *GSTT1* polymorphism and the disease, and the same conclusion was obtained for *GSTP1* variants [177], which found no association with the risk of prostate cancer in men. Another meta-analysis [153] found a significant association of the *GSTM1* polymorphism with the risk of prostate cancer. Still, no such associations were found for *GSTT1* and *GSTP1* deletions, confirming the data of Mo et al. (2009) [177]. Additionally, Gong’a et al. (2012) [153] examined the combination of both genotypes. They demonstrated a significant increase in disease risk in individuals with the double null genotype *GSTM1−*/*GSTT1−* and in individuals with the *GSTT1−*/*GSTP1 Ile*/*Val* variant. Similar trends in the percentage of these combined variants can be observed in the comparisons of combined phenotypes (GSTM1/T1/P1) in the studies conducted by Dróżdż et al. (2012, 2013), Dróżdż-Afelt (2015, 2020, 2022, 2024), and Bombolewska et al. (2013) [117,118,119,120,121,122,123].

The most recent meta-analysis by Cai et al. (2014) [154] also found significant associations between prostate cancer and the *GSTM1* polymorphism, but no such findings for the *GSTT1* variant in the Caucasian population. This meta-analysis did not include studies of *GSTP1* genetic variants. The most recent meta-analysis on this polymorphism by Wei et al. (2013) [196] indicates no association between the *GSTP1 Ile*/*Ile*, *Ile*/*Val*, and *Val*/*Val* variants and an increased risk of prostate cancer. These studies show the same results but also note numerous limitations, including group size, heterogeneity, and the lack of analyses in some populations. It is difficult to find studies conducted in the Polish population that report the occurrence of *GST* polymorphisms and examine the association of these genetic changes with prostate cancer [153,154,177]. On the other hand, the studies by Dróżdż et al. (2012, 2013), Dróżdż-Afelt (2015, 2020, 2022, 2024), and Bombolewska et al. (2013) [117,118,119,120,121,122,123], demonstrating the frequency of genetic variants in *GSTM1*, *GSTT1*, and *GSTP1*, confirm the lack of association between *GST T1* and *P1* polymorphisms and prostate cancer risk, but contradict reports of a possible role of *GSTM1* genetic variants in increasing prostate cancer risk. It should be noted that the studies by Dróżdż et al. (2012, 2013), Dróżdż-Afelt (2015, 2020, 2022, 2024), and Bombolewska et al. (2013) [117,118,119,120,121,122,123] are the first of this kind to include Polish patients with prostate cancer.

## 11. Correlations of *GSTM1* Deletion with Mercury in Prostate Cancer

Detoxification, a process essential to the functioning of organisms, is not uniform across individuals. This indicates that regulatory mechanisms depend highly on genetic predisposition [197]. Many people are exposed to mercury through their diet, with fish consumption a frequent source of methylmercury (MeHg). It has been shown that genetic factors determine differential susceptibility to mercury toxicity. Proteins regulating methylmercury metabolism include reduced glutathione, glucose-6-phosphate dehydrogenase, and γ-glutamyl transpeptidase [198]. In rat studies, Brambila et al. (2002) [199] demonstrated that specific *GST* genes are activated in response to mercury exposure. It has also been noted that individuals representing appropriate genotypes may be better protected against methylmercury-induced cytotoxicity [199].

Analyses by Dróżdż et al. (2012, 2013), Dróżdż-Afelt (2015, 2020, 2022, 2024), and Bombolewska et al. (2013) [117,118,119,120,121,122,123] showed that individuals with prostate cancer who lacked a functional form of the *GSTM1* gene (individuals with the *GSTM1* null genotype) had higher mercury concentrations than individuals with a properly functioning enzyme. Similar results were obtained by Klautau-Guimaraes et al. (2005) [198] in a study of the Murunduku tribe (an Amazonian Indian population) consuming large amounts of fish. They demonstrated high Hg concentrations in the hair of individuals with the *GSTM1−* genotype. Gundacker et al. (2007) [197] also suggest a role for *GST* in mercury metabolism, demonstrating higher mercury concentrations in individuals with the double deletion *GSTM1−*/*GSTT1−* genotype. The studies by Barcelos et al. (2013) [200] are consistent with previous reports [197,198]. They emphasize the link between methylmercury and oxidative stress, which has not yet been well studied in humans. They analyzed samples from Amazonian populations exposed to MeHg from fish. They demonstrated higher blood Hg in individuals with the homozygous *GSTM1* deletion variant [200].

The results of analyses by Dróżdż et al. (2012, 2013), Dróżdż-Afelt (2015, 2020, 2022, 2024), and Bombolewska et al. (2013) [117,118,119,120,121,122,123], similar to those mentioned above, suggest that *GSTM1* deletion may increase the body’s susceptibility to exposure to mercury and its compounds. Furthermore, specific GST isoforms may be expected to be involved in mercury biotransformation in humans [197,198]. These studies indicate an association between the *GSTM1* null variant and mercury concentrations in men with prostate cancer, confirming data from healthy individuals. To investigate the relationship between this association and the occurrence of the disease, studies should be conducted in men with prostate cancer with an established *GSTM1* genotype and increased mercury exposure. Further studies of the effect of *GST* on Hg metabolism are essential to determine the mechanisms and, perhaps, to identify additional, specific enzyme substrates.

## 12. Correlations of *GSTM1−*, *GSTT1−*, and *GSTP1 Val*/*Val* Polymorphisms, Antioxidant Enzymes, Lipoperoxidation, and Trace Elements in Patients with Prostate Cancer

In the studies by Dróżdż et al. (2012, 2013), Dróżdż-Afelt (2015, 2020, 2022, 2024), and Bombolewska et al. (2013) [117,118,119,120,121,122,123], the authors first demonstrated relationships among *GST* polymorphisms, antioxidant enzyme activity, trace element concentrations, and lipid peroxidation intensity. The analyzed parameters are intended to illustrate detoxification and antioxidant mechanisms functioning under various conditions of trace element exposure, in conjunction with the established *GST* genotype in prostate cancer patients. The studies by Dróżdż et al. (2012, 2013), Dróżdż-Afelt (2015, 2020, 2022, 2024), and Bombolewska et al. (2013) [117,118,119,120,121,122,123] revealed relationships that illustrate the functioning of defense mechanisms in individuals with reduced detoxification capacity resulting from specific genetic alterations in *GST*. They demonstrated a significant positive correlation (r = 0.80) between malondialdehyde and Cd concentrations in individuals with the *GSTT1* gene deletion. Thus, as Cd concentration increases in the blood of men with prostate cancer with the *GSTT1−* genotype, there is a simultaneous increase in MDA concentration, which is a significant indicator of increased free radical production [117,118,119,120,121,122,123]. The authors did not find any references for these results. On the other hand, Khansakorn et al. (2012) [201] provide information on the role of *GST* in the biotransformation and detoxification of Cd. They believe that *GST* genes shape susceptibility to this element and report increased blood Cd concentrations in individuals with the *GSTP1 Val*/*Val* genotype, particularly in combination with the *GSTT1−* genotype. They also note that individuals with such genotypes are at increased risk of Cd toxicity.

A study conducted on laboratory rats, analyzing lipoperoxidation in the pancreas of animals exposed to cadmium [202], showed increased oxidative stress in animals exposed to Cd. This was explained by a breakdown of the pancreatic antioxidant barrier, which can lead to organ damage. A similar study was conducted by Koyu et al. (2006) [203], who assessed the effects of Cd on rat livers. The authors point to the role of oxidative mechanisms in Cd-induced liver damage. In the studies by Dróżdż et al. (2012, 2013), Dróżdż-Afelt (2015, 2020, 2022, 2024), and Bombolewska et al. (2013) [117,118,119,120,121,122,123], lipoperoxidation is intensified in men with prostate cancer who have reduced detoxification due to a lack of a functional protein encoded by the *GSTT1* gene in response to increased Cd exposure. In individuals with normal enzyme function, *GSTT1* deletion may lead to reduced antioxidant and cadmium detoxification defenses in individuals with prostate cancer. In the studies by Dróżdż et al. (2012, 2013), Dróżdż-Afelt (2015, 2020, 2022, 2024), and Bombolewska et al. (2013) [117,118,119,120,121,122,123], due to the low number of patients with the *GSTT1−* genotype, this finding therefore represents an interesting proposition for extended analyses of oxidative stress markers in men with prostate cancer who lack a functional *GST* gene.

Further significant correlations between the analyzed markers concerned the concentration of trace elements in individuals with prostate cancer who had the established *GSTT1−* genotype. In these individuals, Dróżdż et al. (2012, 2013), Dróżdż-Afelt (2015, 2020, 2022, 2024), and Bombolewska et al. (2013) [117,118,119,120,121,122,123] demonstrated strong positive correlations between the concentration of iron relative to nickel (r = 0.65), iron relative to zinc (r = 0.68), and nickel relative to mercury (r = 0.70). There are no appropriate scientific references for these results. Analyzing studies on environmental pollutants, familiar sources of exposure to these elements can be identified [204]; the low number of patients with the *GSTT1−* genotype indicates the need for expanded research. However, in individuals with the established *GSTT−* genotype, it should be expected that, with the accumulation of one element, the concentration of the other increases simultaneously, due to the exposure source that incorporates both elements (Fe-Ni, Fe-Zn, Ni-Hg).

Dróżdż et al. (2012, 2013), Dróżdż-Afelt (2015, 2020, 2022, 2024), and Bombolewska et al. (2013) [117,118,119,120,121,122,123] also found significant correlations in men with prostate cancer with the *GSTP1 Val*/*Val* genotype; they demonstrated a negative correlation for CAT and arsenic (r = −0.80) and, separately, for CAT and Cd (r = −0.87). These results may indicate a breakdown in the antioxidant response in individuals exposed to high concentrations of these elements. At high concentrations of Cd or As, SOD activity is reduced in men with prostate cancer with the *GSTP1 Val*/*Val* genotype, perhaps due to the exhaustion of antioxidant capacity in the face of oxidative stress. Cd and As are considered elements responsible for the induction of free radicals [109,205]. Kulikowska-Karpińska et al. (2009) [202] demonstrated a reduction in the activity of antioxidant enzymes, including CAT, in the pancreas of rats treated with Cd. Koyu et al. (2006) [203] also reported a breakdown of the antioxidant barrier in response to Cd exposure, demonstrating a reduction in CAT activity in the liver of rodents exposed to Cd. In their studies on the effects of arsenic on antioxidant mechanisms in rats, Nandi et al. (2006) [206] observed that low doses of As, administered over a shorter period, induce the activation of antioxidant enzymes, whereas prolonged exposure to As suppresses catalase, among others. Despite the low number of patients with the *GSTP1 Val*/*Val* variant, the results found by Dróżdż et al. (2012, 2013), Dróżdż-Afelt (2015, 2020, 2022, 2024), and Bombolewska et al. (2013) [117,118,119,120,121,122,123] present a preliminary analysis of the antioxidant response in individuals with impaired enzyme function exposed to environmental pollutants.

A robust positive correlation (r = 0.89) was also observed in men with prostate cancer with the *GSTP1 Val*/*Val* genotype, indicating the relationship between chromium and exposure to harmful factors in the workplace [117,118,119,120,121,122,123]. Exposure to chromium in chromates and dichromates is associated with carcinogenicity and mutagenicity [207]. In men with prostate cancer with the *GSTP1 Val*/*Val* genotype, a strong association can be observed between high Cr concentrations and individuals with high levels of harmful factors in the workplace. It can be assumed that in this group of individuals with cancer with impaired *GSTP1* activity, Cr accumulation occurs upon exposure to toxic factors in the workplace. These men may have been particularly exposed to Cr compounds at work. Still, the small number of people in this group does not allow for more detailed conclusions and constitutes the basis for extended studies involving people with the *GSTP1 Val*/*Val* genotype [117,118,119,120,121,122,123].

For the *GSTP1 Val*/*Val* genotype in men with prostate cancer, Dróżdż et al. (2012, 2013), Dróżdż-Afelt (2015, 2020, 2022, 2024), and Bombolewska et al. (2013) [117,118,119,120,121,122,123] observed a strong negative correlation (r = −0.95) between MDA and Hg concentrations. These data provide information on an inversely proportional relationship between the concentration of the oxidative stress product MDA and Hg concentration, suspected of inducing free radicals [115]. This result suggests a possible lack of association between oxidative stress induction and mercury exposure in the *Val*/*Val* genotype group. The results of studies by Dróżdż et al. (2012, 2013), Dróżdż-Afelt (2015, 2020, 2022, 2024), and Bombolewska et al. (2013) [117,118,119,120,121,122,123] contradict the reports by Kobal et al. (2004) [208], which indicate the role of long-term mercury exposure in inducing lipoperoxidation. Their studies provided only data on current mercury exposure, which does not allow any conclusions about long-term exposure. Therefore, the analyses should be expanded; given the small number of subjects studied, they provide only a limited basis for further investigation. The established *GST* genotype may determine relationships among detoxification mechanisms under different conditions of trace element exposure. The studies conducted, illustrating the functioning of defense mechanisms during trace element exposure in individuals with reduced detoxification capacity, indicate the need to consider these factors as necessary when assessing the impact of oxidative stress on the bodies of patients with prostate cancer. It can therefore be concluded that there are interrelationships between *GST* polymorphisms, which indicate xenobiotic detoxification capabilities, and the activity of antioxidant mechanisms, i.e., the body’s defenses against free radicals; the level of lipoperoxidation, which means the intensity of oxidative stress; and the concentration of trace elements, which reflect exposure to toxins and deficiencies of essential elements. An important aspect of the future will be conducting extended analyses that incorporate these interesting findings.

## 13. Current Problems of Prostate Cancer

**Figure 3:**
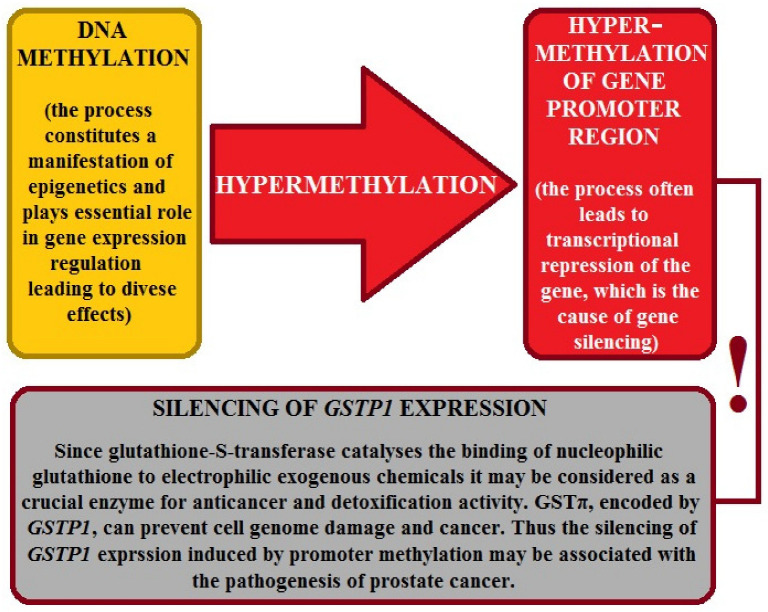
Hypermethylation of the *GSTP1* promoter may cause prostate cancer progression; therefore, silencing of *GSTP1* may be considered as a marker of the disease (based on Zhou et al. (2019) [209]). GSTπ—glutathione S-transferase associated with the *π* gene is encoded by the *GSTP1* polymorphism.

The pathogenesis of prostate cancer is multifactorial, with up to 58% of the risk caused by genetic factors. In this context, *GSTP1* belongs to the metabolic enzyme family and plays a crucial role in preventing the initial occurrence of cancer when the body is exposed to carcinogenic substances. Furthermore, as a tumor suppressor gene, abnormal methylation of the *GSTP1* promoter usually occurs in various cancer types, such as breast cancer, lung cancer, and prostate cancer. Subsequently, hypermethylation of the *GSTP1* promoter in individuals with prostate cancer may cause inactivation of *GSTP1* expression, which may be connected with tumor progression [209]. Thus, epigenetic silencing of *GSTP1* may be considered a marker of the transformation of normal prostate epithelium into prostate cancer (Figure 3). Currently, the role of epigenetic changes in tumor-associated genes in the pathogenesis and development of prostate cancer is attracting interest, especially in the context of investigating potential indicators for cancer diagnosis and treatment [209]. According to the results of meta-analysis conducted by Zhou et al. (2019) [209], the incidence of *GSTP1* promoter methylation was higher in patients with prostate cancer than in those without prostate cancer (OR = 18.58, 95% CI: 9.60–35.95, *p* = 0). Therefore, *GSTP1* promoter methylation was highly correlated with the incidence of prostate cancer. On this basis, the team suggests that the process of methylation of the *GSTP1* promoter may increase the risk of prostate cancer. Finally, researchers performed a subgroup analysis to determine whether the effects of *GSTP1* promoter methylation on prostate cancer risk differ by region. Results demonstrated that *GSTP1* promoter methylation was closely related to the risk of prostate cancer in North America, Asia, and Europe, with stronger correlation in the case of Asia than other continents [209].

Liu et al. (2022) [210] sought to determine whether *GST* gene polymorphisms and metabolic syndrome (MetS) interact to influence prostate cancer risk. The premise was that Mets etiology is considered to be the result of multiple gene-environment interactions and tends to be associated with the occurrence and development of prostate cancer. Furthermore, Mets’ manifestations are often accompanied by inflammation and oxidative stress. There are also reports that in the United States, Mets is connected with an augmented risk of prostate cancer in African American men but not in whites. What is more, African American men with Mets tend to exhibit higher DNA methylation levels, including at *GSTT1* [210]. Researchers noted that the *GSTT1* null genotype in Mets patients was positively correlated with prostate cancer. Moreover, the *GSTT1* null genotype (OR = 2.844, 95% CI: 1.791–4.517), *GSTM1* null genotype (OR = 2.192, 95% CI: 1.395–3.446), and *GSTP1* (*A*/*G* + *G*/*G*) genotype (OR = 2.315, 95% CI: 1.465–3.657) seemed to be associated with prostate cancer susceptibility and malignancy. However, the *GSTM1* null genotype and the influence of *GSTP1* (*AG* + *GG*) on prostate cancer were not significantly related to Mets. Still, the *GSTT1* null genotype significantly increased the threat of prostate cancer, indicating that the *GSTT1* null genotype may serve as a predictor of prostate cancer occurrence in individuals with Mets [210]. The team suggests possible mechanisms that may explain their results. They mention that when the organism struggles with Mets, the expression and activity of *GSTT1* are diminished. In such circumstances, brominated diphenyl ethers (added to many consumer goods as flame retardants) can perturb insulin signaling and inhibit glucose transport, leading to insulin resistance.

**Figure 4:**
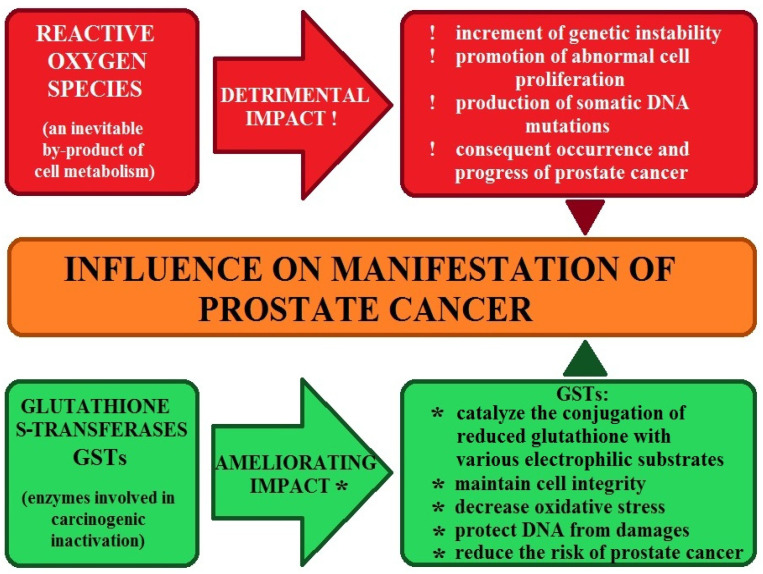
Different effects of reactive oxygen species and glutathione S-transferases activities in the context of the manifestation of prostate cancer (based on Liu et al. (2022) [210]). The symbols in the figure refer to the various effects of reactive oxygen species and glutathione S-transferase activities in the context of the manifestation of prostate cancer.

In contrast, high insulin levels tend to increase the risk of prostate cancer. As an alternative explanation, they note that in patients with Mets, the *GSTT1* gene may be hypermethylated, leading to reduced *GSTT1* expression. In such circumstances, effective detoxification is not provided. When local prostate tissue is simultaneously burdened with escalated oxidative stress, genetic instability, uncontrolled cell divisions, and immune disorders, the risk of prostate cancer increases [210]. Finally, the opposing effects of reactive oxygen species and glutathione S-transferases on prostate cancer risk are shown in Figure 4.

On the other hand, Seven et al. (2025) [211] focus on glutathione S-transferase mu 3 (GSTM3), which also may play a crucial role in the detoxification of carcinogens. For instance, in the context of metastatic prostate cancer, the presence of the *GSTM3* rs7483 polymorphism is significantly associated with an augmented risk of disease progression. Thus mentioned polymorphism may be considered as both a prognostic and predictive marker in the management of prostate cancer. However, the role of the *GSTM3* gene in general tumor biology is probably even more complex, as its expression levels vary significantly across cancer types. Low *GSTM3* expression has been reported in other cancers, including renal cell carcinoma and pancreatic cancer.

In contrast, elevated *GSTM3* expression tends to be reported in such malignancies as hepatocellular carcinoma, colon, bladder, and breast cancer. The diverse expression pattern shown indicates that *GSTM3* may act as either a tumor suppressor or a promoter, depending on the cellular context and cancer type. Moreover, Afro-American men exhibit higher *GSTM3* expression levels than European Americans [211]. Furthermore, the cited researchers noted a significant increase in *GSTM3* expression in metastatic prostate cancer cells, and this observation positioned *GSTM3* as a possible biomarker for aggressive prostate cancer. Tumorsphere models showed even higher *GSTM3* expression than conventional cell lines. In addition, in *GSTM3*-silenced cells, the team observed increased mitochondrial membrane potential and a slight decrease in ROS levels. Nevertheless, silencing *GSTM3* led to cell cycle arrest in the G0/G1 phase, a significant increase in necrosis, and a modest rise in apoptosis. Based on the obtained results, researchers concluded that *GSTM3* may be considered a critical regulator of oxidative stress, mitochondrial function, and cell death pathways in prostate cancer cells [211].

Zhang et al. (2023) [212] studied the association of mutations in the androgen-receptor gene and copy numbers of the androgen-receptor silk protein A complex with glutathione S-transferases T1 and M1 in prostate cancer individuals. Researchers report that the wild-type androgen-receptor gene rs5918762 is of the *TT* type, while the frequencies of the *CC* and *TC* alleles in the prostate cancer group were significantly higher than those in the control group (*p* < 0.05). Furthermore, compared with *TT* type prostate cancer (PC) individuals, PC individuals with *TC* type and *CC* type exhibited higher expression levels of the sex hormone receptor silk protein A complex, as well as higher copy numbers of *GSTT1* and *GSTM1* (*p* < 0.05). Finally, the androgen-receptor gene mutation (T > C) was significantly positively correlated with the expression level of the androgen-receptor silk protein A complex and the copy numbers of *GSTT1* and *GSTM1* [212]. The cumulative conclusion underscored that the obtained results demonstrated that androgen-receptor gene polymorphisms were associated with the expression levels of the androgen-receptor silk protein A complex and the copy numbers of *T1* and *M1* glutathione S-transferases. Subsequently, the team suggests that the analyzed factors can be combined to determine prostate cancer susceptibility and potential disease progression. Thus, the mentioned factors together may serve as a useful prognostic biomarker [212].

Benabdelkrim et al. (2018) [213] remind us that, in addition to xenobiotic detoxification, GSTs possess peroxidase and isomerase activities that can inhibit c-Jun N-terminal kinase (JNK). Eventually, GSTs can also bind non-catalytically to various endogenous and exogenous ligands. However, the matter of the association between *GSTM1* and/or *GSTT1* polymorphisms and prostate cancer risk is pending due to inconsistent and conflicting results. For instance, Benabdelkrim et al. (2018) [213] noted an evident association between the *GSTM1* null genotype and the prostate cancer risk (OR = 3.69, 95% CI: 1.30–10.44; *p* = 0.01), identifying the *GSTM1* null genotype as a factor that may increase individual susceptibility to prostate cancer. On the other hand, the *GSTT1* null genotype did not appear to influence (OR = 0.92, 95% CI: 0.32–2.62; *p* = 0.49). Moreover, researchers reported no statistically significant differences between the double null genotype and prostate cancer risk status. However, they mention that the genes implicated in the metabolic activation or detoxification of carcinogens do not act in isolation. Therefore, evaluating multiple genes is essential to fully understanding this phenomenon.

Additionally, the effects of polymorphisms in low-penetrance genes, such as *GSTs*, are usually not identified correctly until several hundred patients are studied. These statements indicate that the subject in question definitely requires further study with large sample sizes to be fully explained [213]. Even short-term exposure to mercury can cause immunotoxic effects, inflammation, and autoimmune dysfunction. Therefore, mercury-laden residues that enter the bloodstream may subsequently trigger diverse immune responses, reducing its effectiveness against infections. On the other hand, constant exposure to mercury may seriously impair the immune defense system.

Furthermore, such forms of mercury as methyl mercury (MeHg) or inorganic mercury (IHg) may damage cells and induce apoptosis. There is also a connection between mercury exposure and the production of pro-inflammatory agents, such as tumor necrosis factor and interleukin 1 [214]. At the molecular level, mercury impacts oxidative stress, mitochondrial dysfunction, and gene regulation. Antioxidative defense may also be impaired by inhibition of certain antioxidants, such as catalase and glutathione, as well as by increased lipid peroxidation.

**Figure 5:**
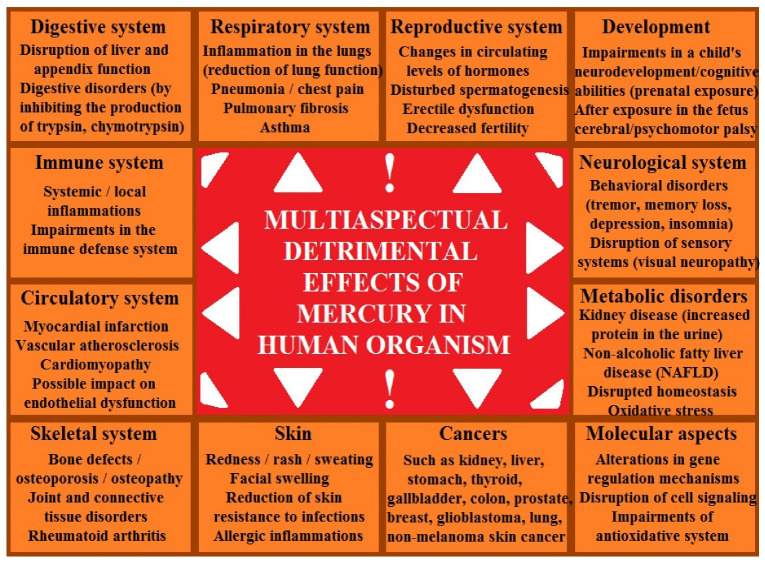
Adverse effects of mercury in relation to various parts of the human organism (based on Charkiewicz et al. (2025) [214]). The symbols in the figure refer to the adverse effects of mercury on various parts of the human body.

Mercury may increase the levels of rhodamine oxygen species, suggesting its ability to act as a catalyst in Fenton-type reactions. Reports indicate that low doses of mercury and its forms can damage pancreatic function and exert detrimental effects on pancreatic cells, as well as on human embryonic kidney and thyroid cell lines [214]. Finally, the International Agency for Research on Cancer and the U.S. Environmental Protection Agency have recognized mercury as a probable human carcinogen (Group 2B). In this context, mercury exposure is connected with the incidence and location of diverse cancers (including prostate cancer). This chemical element is also considered a factor in mononucleosis and leukemia. Generally, even low doses of mercury may trigger a proliferative response in both normal and cancer cells. The engaged mechanism is probably connected with interference with the estrogen receptor, extracellular signal-regulated kinases (ERK1/2), c-Jun N-terminal kinases (JNK), NADPH oxidase, and Transcription Factor (Nrf2). The possible harmful effects of mercury on the human organism are summarized in Figure 5. Obviously, there is an urgent need to integrate public health strategies and future studies to reduce the adverse effects of mercury and its derivatives. The use of safe, effective mercury-free alternatives in everyday life may constitute a possible solution [214].

Coradduzza et al. (2024) [215], based on analyses of reports from current studies, note that, in the case of prostate cancer, several heavy metals, such as Cd, As, Zn, and Fe, are considered essential agents potentially involved in biochemical processes underlying the disease’s pathogenesis. Their impact may include impairment of protection against oxidative damage during cellular respiration, disruption of cell signaling, disturbance of genomic stability and immunity, and deregulation of apoptosis. Some reports indicate that Zn and As appear to exert an impact on the risk of prostate cancer due to specific associations with prostate cancer occurrence. Other studies confirm differences in Fe and Zn concentrations in normal-appearing tissue compared with adjacent cancer areas. On the other hand, Cd exposure from smoking is suspected to exert an influence on disease progression [215]. Ultimately, the hypothesis of a direct relationship between the presence of heavy metals in tissues and the increased risk of carcinogenesis is correct and well justified. However, the levels and types of metal involved in the processes mentioned differ substantially across cancer types. Still, there is consensus that levels of heavy metals are elevated in biological samples from tumor patients.

Nevertheless, the mechanisms underlying subsequent detrimental effects are highly diverse and may include genotoxicity, inhibition of DNA repair, and other mechanisms. Thus, the subject is one of the critical areas of study that requires ongoing research to better understand the relationships between heavy metal exposure and cancer [215]. Human exposure to heavy metals can arise from natural or anthropogenic activities. While their use may be beneficial in many aspects of modern everyday life, environmental exposure can be hazardous and cause toxic effects. Examples of applications of five common toxic heavy metal compounds (Cr, Cd, Hg, Pb, As) are summarized in Table 3 [216].

In this context, chelators constitute pharmaceutical antidotes for the treatment of adverse effects caused by toxic heavy metal intoxications. However, it is essential to know that while some chelating agents can be used for more than one heavy metal, in other cases, the use of a single specific chelator for the treatment of poisoning is advocated. In general, chelating agents are compounds that allow the coordination of two or more donor atoms to a toxic heavy metal, forming a stable ring complex. Thus, chelating agents can efficiently mobilize heavy metal deposits in the urine. Unfortunately, there may be side effects such as renal toxicity and imbalances in electrolytes. For instance, the cadmium-dimercaprol (BAL) complex has been shown to be more nephrotoxic than cadmium alone [216]. There is also no specific antidote for mercury, but chelation therapy is favored in patients with moderate to severe intoxication. Chelation therapy also exhibits only limited efficacy in relation to chronic arsenic toxicity. Moreover, during chelation therapies, monitoring urinary levels of nutritional metals, such as calcium, iron, and zinc, is required to prevent their loss. The doses of chelators should be precisely determined based on the serum levels of toxic heavy metals and the patient’s symptoms and signs of toxicity [216].

Nevertheless, chelation therapy gained attention as a promising medical intervention for toxic heavy metal poisonings. For instance, succimer appears to be a less toxic metal chelator among chelating agents. It has the FDA approval for the clinical management of plumbism. Furthermore, dimercapto-propanesulfonic acid also exhibits fewer side effects. On the other hand, dimercaprol is almost no longer being used as an antidote of choice in the treatment of chronic toxic heavy metal poisonings.

**Figure 6:**
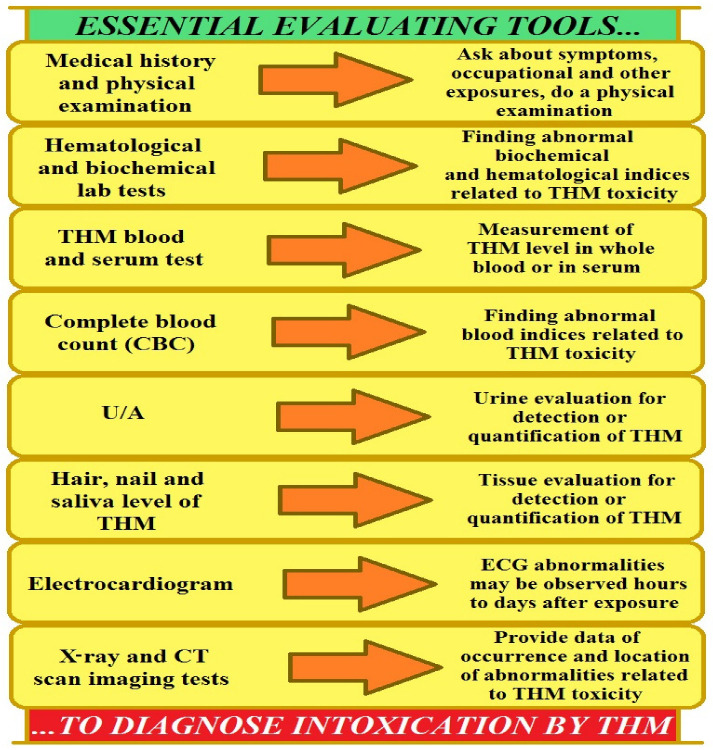
Crucial clinical evaluation tools applied in an attempt to diagnose and determine intoxication by toxic heavy metals (THM) (based on Balali-Mood et al. (2025) [216]). The arrows in the figure refer to crucial clinical evaluation tools applied in an attempt to diagnose and determine intoxication by toxic heavy metals.

In many cases, combination therapy with two chelating agents or co-administration of certain antioxidants with chelators may also be beneficial for achieving therapeutic effects. The development of less toxic chelating agents, as well as the introduction of new treatments with bioactive compounds that exhibit antioxidant and anti-inflammatory properties, constitute subjects of great interest [216]. Ultimately, despite differences in terms of toxicity, application, and the target organs’ effects between various toxic heavy metals, there are specific clinical evaluation tools to diagnose and determine intoxication, as summarized in Figure 6.

Sochacka et al. (2024) [217] remind us that oxidative stress constitutes a frequent therapeutic target for addressing prostate conditions such as prostate hypertrophy, cancer, or chronic prostatitis. However, cancer cells also tend to have reduced activity of certain antioxidant enzymes (due to genetic mutations) compared to normal cells. Subsequently, diminished enzymatic activity can trigger oxidative stress, leading to damage to cellular components in these cancerous cells. Therefore, chemotherapeutic agents such as cisplatin or anthracycline antibiotics act by elevating reactive oxygen species levels in cancer cells. Similarly, inorganic selenium compounds (+4) have been recognized to inhibit cancer cell growth and proliferation more than organic ones (+2) due to their pro-oxidant properties. Unfortunately, the high toxicity of commercially available inorganic Se (+4) compounds limits their applicability. This forces an ongoing search for selenium compounds that exhibit high chemoactivity and, at the same time, relatively low toxicity [217]. In this context, the cited researchers focus on the seleno-triglyceride compound derived from sunflower oil, known as Selol. This novel organic selenium compound shows lower toxicity than sodium selenite (+4), no mutagenic effects, and vigorous cytostatic activity against cancer cell lines, with no side effects on normal cells. The mechanism of action of the mentioned compound involves inducing reactive oxygen species production in cancer cells, leading to excessive oxidative stress that may ultimately result in their apoptosis. Finally, the team reports that the application of selenium compounds increases the activity of antioxidant enzymes in the blood of healthy animal models. In tumor-bearing mice, Selol alters tumor cell morphology, triggering necrotic changes. Moreover, it affects the antioxidant-pro-oxidant potential in the tumor, possibly due to the induction of oxidative stress. Nevertheless, researchers recommend further studies, as Selol appears to break down cancer cells more effectively in small tumors than in larger ones. In contrast, in advanced tumors, it may even accelerate tumor growth when used as monotherapy [217].

**Figure 7:**
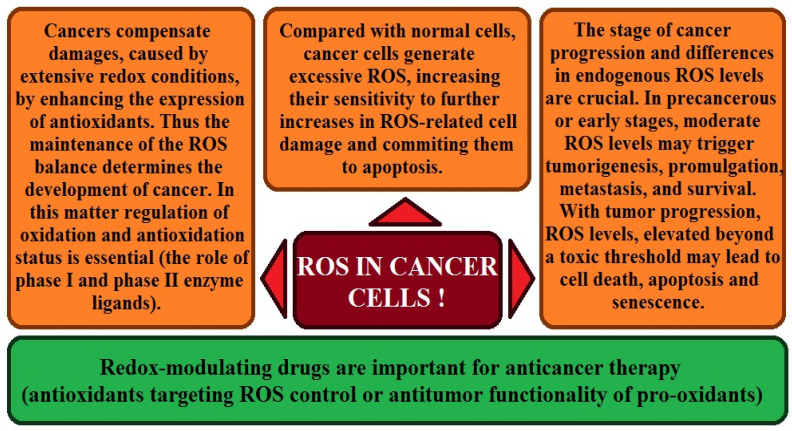
Reactive oxygen species (ROS) in cancer cells in the context of anticancer therapy (based on Lin et al. (2024) [218]).

Nevertheless, antioxidant drugs targeting phase II enzyme pathways have been extensively developed to help control ROS balance in patients with cancer. The phase II enzyme-regulating transcription factor NRF2 (nuclear factor erythroid 2-related factor 2) appears crucial for modulation of cancer development against oxidative stress. Thus, modulating NRF2 activity may be a promising therapeutic approach. However, such therapies should be carefully selected based on tumor type, stage, and other factors [218]. The characterization of reactive oxygen species in cancer cells is summarized in Figure 7.

In general, controlling the balance between reactive oxygen species and redox status is a critical way to address tumor cell occurrence and progression. In this context, alterations in cancer redox activity, driven by antioxidation or increased ROS production, may shift the ROS balance threshold in cancer cells, leading to cell cycle arrest or cell death. Therefore, some anticancer strategies aim to inhibit ROS-mediated cancer occurrence and progression by inducing oxidative damage, such as ROS-mediated apoptosis, or by destabilizing the ROS balance in cancer cells. Therapeutic trials of antioxidants targeting ROS control or studies focused on the antitumor potential of pro-oxidants are gaining interest [218]. However, drugs targeting ROS still appear less effective than more conventional anticancer drugs. There are concerns that the actual sensitivity of tumor cells to ROS may not be so augmented as previously thought. Insufficient biomarkers to measure endogenous redox levels may also constitute a problem. Finally, the complexity of drugs targeting ROS/redox systems requires more thorough characterization.

In the context of prostate cancer, the risk and development of the common impact of the phenomenon of oxidative stress, chemical elements management, and genetic disturbances are briefly characterized in the following three tables (Table 4, Table 5 and Table 6). Oxidative stress factors and inflammation play a significant role in the development of neoplastic conditions and prostate cancer. During the development of prostate cancer, a series of interdependent functional relationships occur, characterized by biochemical pro-antioxidant reactions (Table 4).

The summary of the multifaceted interactions of ions of chemical elements of different chemical nature, presented in Table 5, highlights the importance of this influence. It generates multifaceted biochemical reactions and triggers diverse defense mechanisms at the organ and whole-body levels (Table 5).

In addition to oxidative stress factors and the interactions of ions of chemical elements with various physiological effects, equally important prostate cancer risk factors include gamma interactions, single-nucleotide polymorphisms, and relationships between gene polymorphisms. These are unpredictable and therefore manifest as interconnected, multifaceted reactions (Table 6).

**Figure 8:**
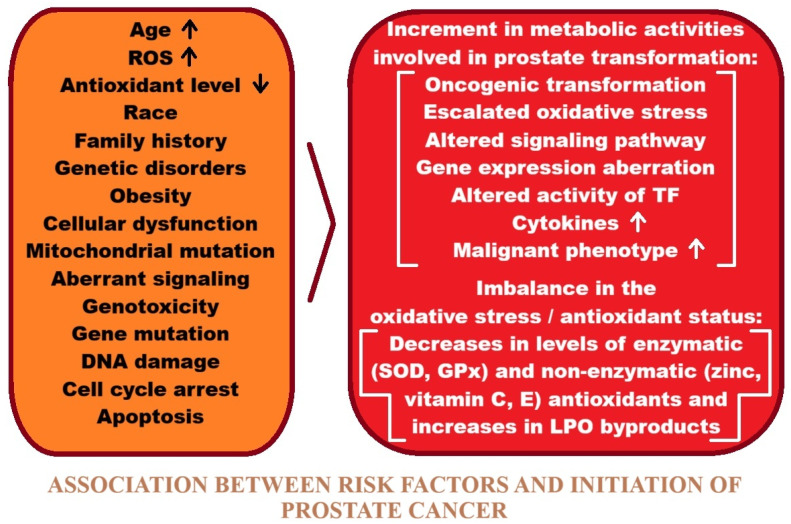
Well-characterized risk factors of prostate cancer and their influence on the possible initiation of the disease, with a particular role of oxidative stress in the phenomenon (based on Udensi and Tchounwou (2016) [224]). The arrows in the figure refer to the increasing or decreasing risk factors for prostate cancer and their influence on the possible initiation of the disease, with a particular role for oxidative stress in the phenomenon.

**Figure 9:**
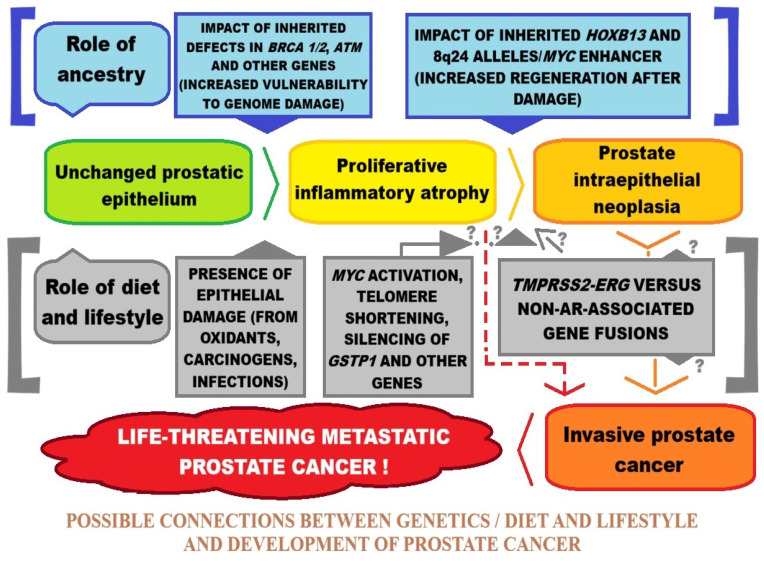
Common impact of ancestry and factors connected with diet and lifestyle on the genetic background of prostate cancer development (based on Nelson et al. (2022) [225]). The symbols, dashed lines, and arrows in the figure refer to the relationships and the course of the dependencies between the impact of ancestry and factors connected with diet and lifestyle on the genetic background of prostate cancer development indicated in the figure.

**Figure 10:**
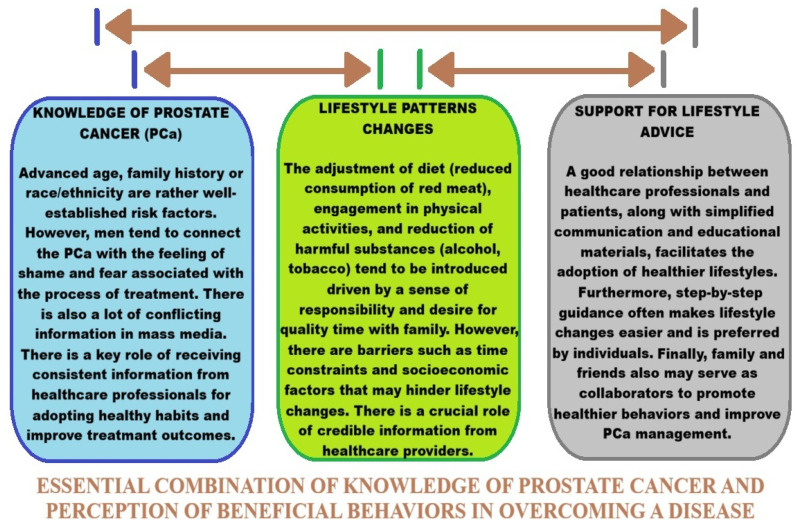
The beneficial role of obtaining consistent information, introducing beneficial behaviors, and supporting lifestyle advice in overcoming prostate cancer or improving treatment outcomes (based on Leitão et al. (2025) [226]). The colors in the figure refer to the various factors (aspects) considered in the figure. The arrows in the figure refer to the mutual (reciprocal) relationships between these factors.

Three figures presented below (Figure 8, Figure 9 and Figure 10) characterize the nature of prostate cancer initiation (Figure 8) and development (Figure 9), and underscore the importance of actions patients take to improve treatment outcomes. In the context of prostate cancer stages, the meanings of oxidative status, genetic agents, and lifestyle factors are collectively presented in the figures (Figure 8 and Figure 9). Escalation of oxidative stress may be considered a consequence of specific risk factors and a key modulating agent of the unfavorable aftereffects [224]; see Figure 8.

Reports of inherited susceptibility underscore the roles of genes involved in prostate cell and tissue repair (*BRCA1* and *BRCA2*, Breast Cancer gene 1 and 2; *ATM*, Ataxia Telangiectasia Mutated) and regeneration (*HOXB13*, Homeobox B13; *MYC*, putative *MYC* enhancer). Furthermore, the absence of *GSTP1* (glutathione S-transferase pi gene) sensitizes prostate cancer cells to mutagenic damage. Finally, *TMPRSS2–ERG* (a fusion of *TMPRSS2*, a transmembrane protease serine 2, and *ERG*, an *ETS*-related gene encoding erythroblast transformation-specific transcription factors) is also detected in prostate cancers from individuals of various races and ethnicities. Despite the frequent occurrence of rearrangements involving AR (androgen receptor) regulated genes in prostate cancers, other sites of DNA breakage and recombination are evident in certain cases. Ultimately, many effects and impacts of the various pathways remain incompletely explained (represented by question marks in the scheme) [225]; see Figure 9.

The actions undertaken by patients with a sense of responsibility and a desire to overcome the disease may be crucial for strengthening the beneficial effects of treatment [226].

All three aspects mentioned in Figure 10 influence one another, and their cumulative impact ensures optimal effect [226].

## 14. Summary and Conclusions

This review focused on determining the frequencies of polymorphic variants in *GSTM1*, *GSTT1*, and *GSTP1*, examining the activity of antioxidant defense enzymes, the intensity of lipid peroxidation, and the concentrations of selected trace elements in patients with prostate cancer. The data were compared with results from a group of healthy controls. A key aim of this review was to show the interrelationships between complex detoxification mechanisms and the concentrations of trace elements, primarily toxic heavy metals, in patients with prostate cancer. For the first time, it reported correlations between *GST* polymorphisms and the activity of antioxidant enzymes, trace element concentrations, and the intensity of lipid peroxidation. This analysis provided medically relevant data indicating whether the levels of toxic trace elements may be associated with polymorphisms of detoxification genes and the activity of antioxidant enzymes in patients with prostate cancer. This review demonstrated the importance of lifestyle, exposure to stressors, diseases, and genetic predisposition. Analyses allowed us to assess whether the concentrations of chemical elements depend on *GST* polymorphisms and, thus, on the body’s detoxification capacity. This paper also analyzed such correlations for individual antioxidant enzymes and malondialdehyde, allowing us to assess the exposure of the studied men to oxidative stress under conditions of limited or normal xenobiotic biotransformation capacity, as determined by molecular testing (*GST* analysis).

This review indicates no significant differences in the frequency of *GST* polymorphisms between individuals with prostate cancer and the control group. It detected *GSTM1* deletion in 47% of individuals with cancer and in 55% of individuals in the control group. It also detected *GSTT1* deletion in 17% of patients and 14% of controls. The distribution of *GSTP1 Ile*/*Ile*, *Ile*/*Val*, and *Val*/*Val* genetic variants was 51%, 39%, and 9% in patients, and 61%, 34%, and 5% in controls, respectively. For antioxidant and detoxification enzymes, a significant difference between the study groups was shown only for SOD activity.

Comparison of measurements of the remaining biomarkers of antioxidant activity (GST, CAT) did not reveal significant differences. Also, in measuring MDA concentration, this review reports no significant differences between patients with prostate cancer and controls. Our paper indicates that As, Cr, Cu, Zn, Cd, and Pb are significant in prostate cancer. Patients had lower concentrations of Cr, Cu, Zn, Cd, and Pb. This review indicates higher Hg concentrations than in individuals with cancer who carried the *GSTM1+* genetic variant. In individuals with prostate cancer and the *GSTM1* genotype, there is a significant positive correlation (r = 0.80) between MDA and Cd levels. In individuals with prostate cancer who have the established *GSTT1* genotype, there are strong positive correlations between Fe and Ni (r = 0.65), Fe and Zn (r = 0.68), and Ni and Hg (r = 0.70). In individuals with prostate cancer carrying the *GSTP1 Val*/*Val* genetic variant, there is a negative correlation for CAT and As (r = −0.80) and separately for CAT and Cd (r = −0.87). A robust positive correlation (r = 0.89) occurs in men with prostate cancer with the *GSTP1 Val*/*Val* genotype, concerning the relationship between chromium and exposure to harmful factors at work. These men may have been particularly occupationally exposed to Cr compounds, but the small number in this group warrants expanded studies involving individuals with the *GSTP1 Val*/*Val* genotype. For the *GSTP1 Val*/*Val* genotype in men with prostate cancer, there is a strong negative correlation (r = −0.95) between the concentration of MDA and Hg.

Analysis of the relationships between *GSTM1*, *GSTT1*, and *GSTP1* polymorphisms with the occurrence of prostate cancer did not reveal an association of these genetic variants with an increased risk of this cancer. Lower SOD activity in patients may be due to impaired antioxidant defense systems. Low concentrations of Cu and Zn, elements necessary for the mechanisms of SOD, may be associated with reduced SOD activity. The higher As concentration in patients with prostate cancer provides a basis for further studies of exposure to this element in prostate cancer cases. The different concentrations of chemical elements indicate differential exposure between individuals with cancer and healthy controls. This review suggests that *GSTM1* deletion may be a factor increasing the body’s susceptibility to exposure to mercury and its compounds in patients with prostate cancer. In men with prostate cancer with the *GSTT1−* genotype, lipoperoxidation processes are intensified in response to increased Cd exposure. In individuals with the *GSTT1+* genotype, this deletion may reduce the antioxidant defense functions and Cd detoxification. Based on the correlations between trace elements in patients with prostate cancer and the *GSTT1* genotype, it can be concluded that the accumulation of one element is accompanied by an increase in the concentration of another (Fe, Ni, Hg), possibly due to a common source of exposure. These results indicate a decline in the antioxidant response in patients exposed to high concentrations of As and Cd and carrying the *GSTP1* genetic variant (likely associated with less efficient detoxification).

This review examines the relationships among polymorphisms, antioxidant mechanisms, lipid peroxidation, and trace element concentrations in men with prostate cancer. Trace elements may be associated with polymorphisms in detoxification genes and with antioxidant enzyme activity in patients. This review indicates the need to consider these factors in further assessing the risks of oxidative stress exposure. Despite good progress in understanding the origins of the disease, which included the discovery of the role of testosterone, scientists are still trying to define both environmental and genetic factors that increase susceptibility to this cancer. This paper examines the interactions between exposure to environmental stressors and increased susceptibility to cancers, including cancers of the male reproductive system. Differentiated chemical elements introduced into the body may play a significant role in this process. Perhaps individuals with cancer have a disturbed antioxidant enzyme status, which could be a basis for decreased defense against carcinogenic factors or result from disturbed body balance caused by the carcinogenic process. In turn, studies of repair gene polymorphism may indicate disorders of proteins needed for the organism’s defense. The review presented provides data for conclusive population studies of the impact of environmental factors on the carcinogenic process in the male reproductive system.

## Figures and Tables

**Table 1:** Global incidence, prevalence, mortality, and disability-adjusted life-years of six urologic diseases from 1990 to 2021 (based on Zi et al. (2024) [53]).

Year	BPH	UTI	Urolithiasis	Bladder Cancer	Kidney Cancer	Prostate Cancer
1990						
Incidence (×10^5^, 95% UI)	64.06 (50.00–79.95)	2698.15 (2417.07–2992.89)	731.16 (604.47–900.45)	2.60 (2.43–2.72)	1.60 (1.55–1.64)	5.06 (4.81–5.25)
Prevalence (×10^5^, 95% UI)	507.06 (387.36–656.93)	51.37 (46.11–56.88)	27.63 (22.67–33.75)	13.29 (12.49–13.84)	7.52 (7.28–7.71)	35.96 (34.45–37.05)
Mortality (×10^5^, 95% UI)	-	1.03 (0.94–1.16)	0.11 (0.07–0.13)	1.23 (1.13–1.30)	0.77 (0.75–0.80)	2.12 (1.94–2.24)
DALYs (×10^5^, 95% UI)	10.11 (6.06–15.56)	33.93 (30.33–37.19)	4.95 (3.79–6.10)	27.33 (24.72–28.94)	22.92 (21.9–23.86)	41.47 (37.54–44.02)
ASIR (1/100,000, 95% UI)	335.00 (262.28–414.71)	5294.50 (4784.14–5869.53)	1602.48 (1315.54–1983.36)	6.90 (6.46–7.23)	3.89 (3.75–3.99)	32.64 (30.86–33.86)
ASPR (1/100,000, 95% UI)	2899.84 (2240.05–3682.58)	100.96 (91.2–111.55)	60.72 (49.44–74.41)	33.41 (31.49–34.81)	17.26 (16.76–17.67)	218.33 (208.48–225.67)
ASMR (1/100,000, 95% UI)	-	2.84 (2.58–3.20)	0.29 (0.20–0.35)	3.51 (3.23–3.70)	1.99 (1.91–2.06)	16.35 (15.02–17.28)
ASDR (1/100,000, 95% UI)	57.48 (34.56–87.77)	74.98 (67.72–81.95)	11.38 (8.72–13.99)	71.05 (64.72–75.17)	53.02 (50.96–54.93)	275.30 (251.66–292.14)
2021						
Incidence (×10^5^, 95% UI)	137.88 (109.08–170.15)	4491.02 (4008.94–4998.43)	1059.84 (883.49–1286.45)	5.40 (4.95–5.83)	3.88 (3.65–4.07)	13.24 (12.17–14.00)
Prevalence (×10^5^, 95% UI)	1125.02 (881.32–1426.34)	85.61 (76.74–94.97)	40.21 (33.64–48.16)	30.26 (28.23–32.24)	19.61 (18.62–20.52)	103.88 (97.06–109.04)
Mortality (×10^5^, 95% UI)	-	3.00 (2.68–3.24)	0.18 (0.14–0.21)	2.22 (2.01–2.42)	1.61 (1.50–1.69)	4.32 (3.82–4.64)
DALYs (×10^5^, 95% UI)	22.36 (13.46–34.03)	68.48 (61.75–73.69)	6.93 (5.68–8.50)	43.97 (40.64–48.14)	40.16 (38.07–42.47)	81.42 (71.77–88.09)
ASIR (1/100,000, 95% UI)	326.12 (258.88–400.32)	5531.88 (4965.44–6161.01)	1242.84 (1034.93–1506.99)	6.35 (5.80–6.85)	4.52 (4.26–4.75)	34.05 (31.27–36.00)
ASPR (1/100,000, 95% UI)	2782.59 (2191.58–3508.04)	105.35 (94.44–116.98)	47.10 (39.41–56.31)	34.91 (32.54–37.19)	22.70 (21.54–23.76)	260.05 (243.39–272.68)
ASMR (1/100,000, 95% UI)	-	3.71 (3.31–4.01)	0.21 (0.17–0.25)	2.68 (2.42–2.93)	1.91 (1.78–2.01)	12.63 (11.16–13.55)
ASDR (1/100,000, 95% UI)	55.12 (33.21–83.48)	83.74 (75.54–90.22)	8.15 (6.68–9.99)	51.58 (47.56–56.42)	47.33 (44.76–50.07)	217.83 (192.65–235.53)
1990–2021						
ASIR (EAPC, 95% CI)	0.03 (−0.02–0.08)	0.15 (0.10–0.20)	−0.87 (−0.91 to −0.84)	−0.36 (−0.41 to −0.30)	0.53 (0.40–0.66)	−0.06 (−0.20–0.08)
ASPR (EAPC, 95% CI)	−0.01 (−0.06–0.04)	0.15 (0.10–0.19)	−0.87 (−0.90 to −0.84)	0.10 (0.01–0.19)	0.98 (0.80–1.16)	0.42 (0.27–0.58)
ASMR (EAPC, 95% CI)	-	1.02 (0.95–1.10)	−1.02 (−1.24 to −0.80)	−0.98 (−1.03 to −0.94)	−0.14 (−0.21 to −0.07)	−1.05 (−1.14 to −0.95)
ASDR (EAPC, 95% CI)	0.00 (−0.05–0.05)	0.42 (0.35–0.49)	−1.15 (−1.28 to −1.02)	−1.19 (−1.24 to −1.13)	−0.37 (−0.43 to −0.30)	−0.96 (−1.05 to −0.88)

BPH—benign prostatic hyperplasia; UTI—urinary tract infections; DALYs—disability-adjusted life-years; ASIR—age-standardized incidence rate; ASPR—age-standardized prevalence rate; ASMR—age-standardized mortality rate; ASDR—age-standardized DALYs rate; EAPC—estimated annual percentage change; CI—confidence interval; UI—uncertainty intervals.

**Table 2:** Levels of antioxidants by prostate-specific antigen PSA status for middle-aged men (40–64.9) and older men (≥65 years); based on Lin et al. (2023) [140].

	Middle-Aged Men Aged 40–64.9 Years					Older Men Aged ≥65 Years				
Factors	Total Mean +/− SE ^a^ (n = 3651)	PSA ≤ 4 Mean +/− SE ^a^ (n = 3515)	PSA 4.01–10 Mean +/− SE ^a^ (n = 120)	PSA > 10 Mean +/− SE ^a^ (n = 16)	*p*-Value ^b^	Total Mean +/− SE ^a^ (n = 2119)	PSA ≤ 4 Mean +/− SE ^a^ (n = 1716)	PSA 4.01–10 Mean +/− SE ^a^ (n = 316)	PSA > 10 Mean +/− SE ^a^ (n = 87)	*p*-Value ^b^
Endogenous antioxidants										
Bilirubin (µmol·L^−1^)	14.28 +/− 0.12	14.27 +/− 0.1	14.7 +/− 0.7	12.5 +/− 0.8	0.077	14.9 +/− 0.2	15.0 +/− 0.2	14.9 +/− 0.4	14.3 +/− 0.4	0.298
Albumin (g·L^−1^)	43.39 +/− 0.08	43.4 +/− 0.08	42.5 +/− 0.3	40.6 +/− 0.9	<0.001	41.9 +/− 0.1	42.0 +/− 0.1	41.9 +/− 0.2	40.6 +/− 0.2	<0.001
Uric acid (µmol·L^−1^)	363.78 +/− 1.94	364.00 +/− 2.0	357.9 +/− 10.5	343.9 +/− 21.5	0.514	368.2 +/− 2.3	366.7 +/− 2.4	373.7 +/− 6.2	381.5 +/− 20.3	0.392
Dietary antioxidants										
Vitamin A, RAE ^c^ (µg)	687.9 +/− 12.2	686.9 +/− 12.3	720.1 +/− 52.8	707.9 +/− 170	0.815	753.7 +/− 20.4	757.7 +/− 22.6	766.2 +/− 57.4	606 +/− 48.2	0.011
Vitamin B2 (mg)	2.6 +/− 0.03	2.6 +/− 0.03	2.5 +/− 0.2	2.5 +/− 0.4	0.589	2.4 +/− 0.04	2.4 +/− 0.04	2.3 +/− 0.1	2.2 +/− 0.2	0.626
Vitamin C (mg)	92.4 +/− 2.1	92.0 +/− 2.1	103.5 +/− 9.5	112.3 +/− 43.6	0.443	92.4 +/− 2.0	92.0 +/− 2.4	96.9 +/− 6.4	82.9 +/− 9.9	0.519
Vitamin D (µg)	5.6 +/− 0.2 (n = 2131)	5.5 +/− 0.2 (n = 2051)	7.2 +/− 1.7 (n = 71)	3 +/− 0.7 (n = 9)	<0.001	5.6 +/− 0.2 (n = 1116)	5.6 +/− 0.2 (n = 905)	5.7 +/− 0.6 (n = 161)	4.6 +/− 0.5 (n = 50)	0.141
Vitamin E (mg)	8.6 +/− 0.1	8.6 +/− 0.1	8.4 +/− 0.4	9.6 +/− 2	0.759	7.4 +/− 0.1	7.5 +/− 0.2	7.3 +/− 0.3	6.6 +/− 0.4	0.117
Alpha carotene (µg)	405.3 +/− 18.1	403.6 +/− 18.4	468.7 +/− 97.7	367.7 +/− 168.9	0.789	472.5 +/− 28.2	486.9 +/− 29.6	431.5 +/− 66.4	294.7 +/− 77.7	0.072
Selenium (µg)	132.1 +/− 1.4	132.1 +/− 1.4	131.3 +/− 5.7	126.3 +/− 13	0.869	105.3 +/− 1.3	105.6 +/− 1.3	103.8 +/− 2.8	106.9 +/− 7	0.679
Lycopene (µg)	6767.1 +/− 227.2	6761.9 +/− 242.8	6869.7 +/− 1052.5	7631 +/− 2463.2	0.939	5447.5 +/− 291.4	5452.1 +/− 309.1	5538.8 +/− 698.8	4964.3 +/− 819.1	0.824
Lutein + zeaxanthin (µg)	1581.4 +/− 66	1582.1 +/− 68.1	1501.8 +/− 149.8	2174.9 +/− 854.5	0.656	1548.9 +/− 81.6	1559.6 +/− 89.0	1504.2 +/− 154.5	1473.2 +/− 233.5	0.886
Beta-cryptoxanthin (µg)	115.9 +/− 5.0	115.4 +/− 5.1	132.2 +/− 20.6	113.7 +/− 44.4	0.750	133.5 +/− 5.6	132.8 +/− 6.7	147.9 +/− 23.2	92.9 +/− 15.1	0.057
Folate, DFE ^c^ (µg)	467.8 +/− 6.6	467.4 +/− 6.7	473.7 +/− 22	544.7 +/− 78.2	0.570	427.7 +/− 6.3	429.2 +/− 7.3	430.5 +/− 24.2	377.8 +/− 20.5	0.055

^a^ weighted mean +/− standard error based on the NHANES (National Health and Nutrition Examination Survey) sampling weights. ^b^ compared antioxidant levels among the three PSA prostate-specific antigen groups using the univariate weighted multinomial logistic model. ^c^ RAE: Retinol activity equivalents, DFE: Dietary folate equivalents; PSA values in ng·mL^−1^.

**Table 3:** Toxic heavy metal compounds, their chemical formula, and applications (based on Balali-Mood et al. (2025) [216]).

Five Common Toxic Heavy Metals Include Cr, Cd, Hg, Pb, and As		
**Chromium (Cr) compounds**	**Chemical formula**	**Uses**
Barium chromate (Cr^6+^)	BaCrO_4_	Anticorrosive, safety matches, paint pigment
Calcium chromate (Cr^6+^)	CaCrO_4_	Batteries, metallurgy,
Chromic acid (Cr^6+^)	H_2_CrO_4_	Oxidizer, electroplating
Lead chromate (Cr^6+^)	PbCrO_4_	Paints and dyes yellow pigment
Potassium dichromate (Cr^6+^)	K_2_Cr_2_O_7_	Leather tanning, oxidizer of organic compounds, porcelain painting
Chromic chloride (Cr^3+^)	CrCl_3_	Total parenteral nutrition
Chromium picolinate (Cr^3+^)	C_18_H_12_Cr N_3_O_6_	Nutritional supplement
Chromic fluoride (Cr^3+^)	CrF_3_	Protect against moth for wool and silk, fixing material in the dye industry
Chromic oxide (Cr^3+^)	Cr_2_O_3_	Metal plating, wood treatment
Chromite ore (Cr^3+^)	FeCr_2_O_4_	Water tower treatment
**Cadmium (Cd) compounds**	**Chemical formula**	**Uses**
Cadmium carbonate	CdCO_3_	Paints and dye pigment
Cadmium chromate	CdCrO_4_	Chemical manufacturing
Cadmium sulfide	CdS	Solar cells, light-emitting diodes
Cadmium oxide	CdO	Paints and dye pigment
Cadmium telluride	CdTe	Solar cells
**Mercury (Hg) compounds**	**Chemical formula**	**Uses**
Mercury acetate	Hg(CH_3_CO_2_)_2_	Chemical and pharmaceutical manufacturing
Mercury chloride	HgCl_2_	Disinfectant, photography
Methylmercury chloride	CH_3_HgCl	Chemical manufacturing
Phenylmercury acetate	CH_3_CO_2_HgC_6_H_5_	Used in pesticides
**Lead (Pb) compounds**	**Chemical formula**	**Uses**
Lead acetate	Pb(CH_3_CO_2_)_2_	Chemical manufacturing, pigment production
Lead nitrate	Pb(NO_3_)_2_	Manufacture of explosives, pigment production, and chemical manufacturing
Lead chloride	PbCl_2_	Chemical manufacturing, ceramics manufacturing
Lead thiocyanate	Pb(SCN)_2_	Manufacture of explosives
Lead oxides (tetroxide and dioxide)	Pb_3_O_4_ and PbO_2_	Battery and pigment manufacturing
**Arsenic (As) compounds**	**Chemical formula**	**Uses**
Sodium arsenite	NaAsO_2_	Used in pesticides
Arsine	AsH_3_	Chemical warfare agent, metals refining industries
Arsenates of sodium, calcium, potassium, and lead	AsO_4_^−^	Disinfectant, paper industries

**Table 4:** The impact of oxidative stress and inflammation on prostate cancer development (based on Liou et al. (2024) [219]).

Oxidative Stress and Inflammation in Prostate Cancer	Conclusion
Higher levels of intracellular ROS (like superoxide or H_2_O_2_) tend to be present in human prostate cancer cells than in prostate primary epithelial cells. Similarly, human prostate cancer tissue samples tend to present increased levels of hydrogen peroxide and Nox1 (NADPH oxidase 1) expression. Finally, increased mRNA levels of Nox1, Nox2, and Nox5 are also observed in human malignant prostate cell lines. On the other hand, inhibition of ROS production by the NADPH oxidase (Nox) system is often associated with impairments in the migration and proliferation of prostate cancer cells. Knockdown of Nox5 in certain human malignant prostate cancer cell lines may also lead to impaired proliferation of cancer cells.	ROS may play a crucial role in the malignant cell behaviors of prostate cancer. In this context, ROS appear to be essential for prostate cancer cell growth.
Increased plasma C-reactive protein levels tend to be negatively correlated with poor cancer-specific, overall, and disease-free survival. Simultaneously, higher C-reactive protein levels are rather positively associated with a higher risk of clinically significant prostate cancer, while higher circulating C-reactive protein levels tend to be positively linked to a higher Gleason score in prostate cancer individuals.	Since a high C-reactive protein level indicates elevated inflammation in the body, the escalated process of inflammation is also connected with the development of prostate cancer.
The levels of oxidative stress and inflammation are rather elevated in the case of individuals with benign prostatic hyperplasia and prostate cancer with various risk levels when compared to healthy controls. Furthermore, the levels of total antioxidant status tend to be decreased in prostate cancer patients. On the other hand, individuals who underwent surgery tend to exhibit lower levels of oxidative stress and inflammation after surgery. Finally, serum prostatic-specific antigen levels as well as levels of 8-OHdG (oxidized nucleosides of DNA) are rather elevated in high-risk prostate cancer individuals than in healthy controls. In contrast, healthy individuals tend to have higher levels of the beneficial enzyme glutathione S-transferase than prostate cancer patients.	Both oxidative stress and escalated inflammation may trigger prostate cancer development, progression, and invasiveness.

**Table 5:** The ambiguous roles of trace elements and macroelements in the development of prostate cancer in the context of oxidative stress (based on Banas et al. (2010), Rahmati et al. (2018), Maly and Hofmann (2018) [220,221,222]).

Element	Role of Chemical Elements in the Development of Prostate Cancer (in the Context of Oxidative Stress)
Manganese (Mn)	For early prostate cancer stage cases (Gleason grade 2), the tissue concentration of Mn may reach a value almost 10 times higher than for healthy and hyperplastic individuals. In this context, Mn, as a transition metal, can catalyze the Fenton reaction. Subsequently, such a reaction may cause site-specific accumulation of free radicals and initiate biomolecular damage processes. Therefore, an elevated Mn level in Gleason grade 2 patients may even enhance prostatic carcinogenesis. On the other hand, Mn is a significant component of the free-radical defense system (Mn-SOD protects endothelial cells, mitochondria, and red blood cells from damage). Therefore, individuals with more advanced cancer (Gleason grades 3 and 4) tend to exhibit lower Mn levels than those with less advanced cancer (Gleason grade 2), and this phenomenon may be associated with loss of Mn-SOD’s beneficial activity.
Iron (Fe)	Since Fe is an integral part of certain proteins and enzymes, it appears essential for maintaining physiological function and for regulating cell differentiation. However, any unregulated level of Fe^2+^ may catalyze the generation of hydroxyl radicals from superoxide and hydrogen peroxide via the Fenton reaction. In contrast, highly reactive hydroxyl radicals may trigger lipid peroxidation and the degradation of other macromolecules (cell damage). Furthermore, Fe contributes to disease development by serving as a nutrient for microbial and neoplastic cells or by promoting inflammation and increased cancer cell growth. There is a tendency for higher levels of Fe in the malignant prostate tissues when compared to the noncancerous ones. What is more, Fe concentrations tend to increase with advancing cancer stage (about 3 times for Gleason grades 2 and 3, and almost 6 times higher for Gleason grade 4 compared with healthy prostate tissue). Therefore, there is a possibility that high body Fe burden may be connected with increased risk of prostate cancer development.
Copper (Cu)	Engaged in the improvement of the defensive capabilities of the organism as a cofactor of superoxide dismutase. In this context, the concentration of Cu rather decreases with more advanced stages of prostate cancer (reduced defensive potential). On the other hand, Cu may be considered as a switcher that turns on angiogenesis in tumor cells. In this matter, elevated Cu concentrations are present not only in cancerous tissues (Gleason grades 2–4) but also in hyperplastic tissues, compared with healthy controls. Especially in Gleason grade 2 cases, Cu concentrations may reach levels even 15 times higher than in healthy prostate tissue. Furthermore, Cu acts as a catalyst in the Fenton reaction, altering its oxidation state and generating highly reactive oxygen species. This phenomenon may conjoin Cu with prostate cancer development in the first stage of neoplasm formation. At the same time, a high level of this element in individuals with Gleason grade 2 may also be associated with the formation of new blood vessels for tumor growth.
Zinc (Zn)	Conflicting information is often reported regarding the role of Zn in carcinogenesis (as a promoting or a suppressing agent). Similar to Mn and Cu, the level of Zn also tends to decrease gradually with more advanced Gleason grades, suggesting possible impairment of the antioxidative potential. However, an increase in Zn level is more pronounced in hyperplastic prostate tissues than in healthy ones, and the highest Zn concentration is observed in Gleason grade 2 tissues, with values almost 6 times higher than in healthy prostate tissue. It suggests that Zn availability is essential for the function of highly proliferative cell systems. That elevated Zn concentrations may promote disorders of the prostate gland, at least in the early stages of disease (neoplastic transformation and tumor growth).
Calcium (Ca)	There is clinical and laboratory evidence conjoining high intake of calcium and dairy products with increased risk of prostate cancer development. The mechanism involves suppressing the production of 1,25-dihydroxyvitamin D3, which is independent of vitamin D receptors. However, the relationship between total calcium intake and prostate cancer can rather be observed in the developed countries. Moreover, the presence of confounding factors, such as unequal reporting of articles and errors in calculating calcium amounts, constitutes a serious limitation for drawing unambiguous conclusions. On the other hand, calcium ion (Ca^2+^) signaling may play an important role in the function of regulatory pathways involved in prostate cancer development. Moreover, the intracellular propagation of a calcium-mediated signal and the import of downstream molecules into the nucleus can be considered as common events in the dysregulation involving the cell and its environment. Finally, calcium channels and G protein-coupled receptors are among the favored druggable targets in the development of therapeutics.

**Table 6:** Involvement of molecular pathogenesis in prostate cancer development (based on Kohli and Tindall (2010) [223]).

Genetic Disturbance	Conclusion
Gene fusions	Recurrent gene fusions involving two members of the oncogenic *ETS* (erythroblast transformation specific) family of transcription factors, *ERG* (ETS-related gene) and *ETV1* (ETS variant 1), and the 5′ untranslated region of *TMPRSS2* (transmembrane protease, serine 2) on chromosome 21 have been identified in 50% to 70% of malignant cells of localized tumors. Furthermore, fusion of *TMPRSS2* with *ERG* results in androgen-regulated *ERG* expression and is detected in essentially all malignant cells within a tumor focus, indicating a possible role in cancer initiation. However, there are still ambiguous reports about its actual impact on preneoplastic prostate intraepithelial neoplasia. In the matter of relatively common abnormalities in prostate cancer leading to prostate tumorigenesis, it is also unclear whether a connection exists between fusion gene rearrangements and molecular pathways, such as *PTEN* (phosphatase and tensin homolog) loss, *PI3* kinase (phosphoinositide 3-kinase) pathway activation, and *MYC* (v-myc myelocytomatosis viral oncogene homolog) amplification.
Single-nucleotide polymorphisms (SNPs)	Large genome-wide association studies conducted across different geographic populations have identified several common germline risk alleles, including SNPs in the 8q24 region and other groups of SNPs. Obviously, the mere presence of a single SNP confers a rather marginal increased risk, which may be further augmented by multiple disease risk alleles, resulting in a cumulative OR for cancer development. Finally, risk assessment for prostate cancer has also resulted in elaborating prevention strategies in “at-risk” populations.

## Data Availability

This paper is a review article. Archived datasets analyzed or generated during the preparation of this paper are included in the list of sources used at the end of the text.

## References

[1] WHO (World Health Organization) (2009). Guidelines for the Psychosocially Assisted Pharmacological Treatment of Opioid Dependence.

[2] WHO (World Health Organization) (2009). International Statistical Classification of Diseases and Related Health Problems, ICD-10.

[3] WHO (World Health Organization) (2010). Laboratory Manual for the Examination and Processing of Human Semen. WHO Library Cataloguing-in-Publication Data.

[4] WHO (World Health Organization) (2016). https://apps.who.int/iris/bitstream/handle/10665/250141/9789241511353-eng.pdf?sequence=1.

[5] WHO (World Health Organization) (2019). International Statistical Classification of Diseases and Related Health Problems, ICD-10.

[6] WHO (World Health Organization) (2019). World Report on Vision.

[7] WHO (World Health Organization) (2019). Launches First World Report on Vision. https://www.who.int/news/item/08-10-2019-who-launches-first-world-report-on-vision.

[8] WHO (World Health Organization) (2024). International Statistical Classification of Diseases and Related Health Problems, ICD-11 (Version: 05/2024). https://icd.who.int/browse/2024-01/mms/en.

[9] WHO (World Health Organization) (2024). Library Cataloguing-in-Publication Data. The Global Burden of Disease: 2004 Update. https://apps.who.int/iris/bitstream/handle/10665/43942/9789241563710_eng.pdf.

[10] Aitken R.J., Skakkebaek N.E., Roman S.D. (2006). Male reproductive health and the environment. Med. J. Aust..

[11] Myrup C., Westergaard T., Schnack T., Oudin A., Ritz C., Wohlfahrt J., Melbye M. (2008). Testicular Cancer Risk in First- and Second-Generation Immigrants to Denmark. J. Nat. Cancer Inst..

[12] Goyer R.A., Liu J., Waalkes M.P. (2004). Cadmium and cancer of prostate and testis. Biometals.

[13] Battisti V., Maders L.D.K., Bagatini M.D., Reetz L.G.B., Chiesa J., Battisti I.E., Gon J.F., Duarte M.M.F., Schetinger M.R.C., Morsch V.M. (2011). Oxidative stress and antioxidant status in prostate cancer patients: Relation to Gleason score, treatment and bone metastasis. Biomed. Pharmacother..

[14] Tang Y.M., Green B.L., Chen G.F., Thompson P.A., Lang N.P., Shinde A., Lin D.X., Tan W., Lyn-Cook B.D., Hammons G.J. (2000). Human CYP1B1 Leu432Val gene polymorphism: Ethnic distribution in African-Americans, Caucasians and Chinese; oestradiol hydroxylase activity; and distribution in prostate cancer cases and controls. Pharmacogenetics.

[15] Srivastava D.S., Mandhani A., Mittal B., Mittal R.D. (2005). Genetic polymorphism of glutathione S-transferase genes (GSTM1, GSTT1 and GSTP1) and susceptibility to prostate cancer in Northern India. BJU Int..

[16] Ricks-Santi L., Mason T., Apprey V., Ahaghotu C., McLauchlin A., Josey D., Bonney G., Dunston G.M. (2010). p53 Pro72Arg polymorphism and prostate cancer in men of African descent. Prostate.

[17] Mittal R.D., George G.P., Mishra J., Mittal T., Kapoor R. (2011). Role of Functional Polymorphisms of P53 and P73 Genes with the Risk of Prostate Cancer in a Case-Control Study from Northern India. Arch. Med. Res..

[18] Wang J., Cheng Q., Luo F., Wan Y., Liang H. (2025). Invastigating bidirectional causality between prostate cancer and inflammatory factors: A 2-sample Mendelian randomization analysis. Medicine.

[19] Robinson J., Banerjee I., Banerjee I. (2025). Testosterone Replacement in Prostate Carcinoma: A Systematic Review of an Emerging Paradigm and Therapeutic Potential. Cureus.

[20] Van Blarigan E.L., McKinley M.A., Washington S.L., Cooperberg M.R., Kenfield S.A., Cheng I., Gomez S.L. (2025). Trends in Prostate Cancer Incidence and Mortality Rates. JAMA Netw. Open..

[21] Xue M., Guo W., Zhou Y., Meng J., Xi Y., Pan L., Ye Y., Zeng Y., Che Z., Zhang L. (2025). Age-sex-specific burden of urological cancers attributable to risk factors in China and its provinces, 1990–2021, and forecasts with scenarios simulation: A systematic analysis for the Global Burden of Disease Study 2021. Lancet Reg. Health West. Pac..

[22] Zhang H., Huang D., Zhang Y., Wang X., Wu J., Hong D. (2023). Global burden of prostate cancer attributable to smoking among males in 204 countries and territories, 1990–2019. BMC Cancer.

[23] Zhang X., Li Y., Yan C., Ma L., Yu M., Yang Y., Lin S., Zhao R., Peng L. (2025). Global trends in testicular and prostate cancer among adolescents and young adult males aged 15–49 years, 1990–2021: Insights from the GBD study. Sci. Rep..

[24] Sabanegh E.S., Dada R., Burns W., Agarwal A. (2010). Male Infertility and Testicular Cancer—Points of Common Causality. Eur. Urol. Rev..

[25] Fingerhut M.A., Halperin W.E., Marlow D.A. (1991). Cancer mortality in workers exposed to 2,3,7,8-tetrachlorodibenzo-p-dioxin. N. Engl. J. Med..

[26] García Sánchez A., Antona J.F., Urrutia M. (1992). Geochemical prospection of cadmium in a high incidence area of prostate cancer, Sierra de Gata, Salamanca, Spain. Sci. Total Environ..

[27] Gao X., LaValley M.P., Tucker K. (2005). Prospective studies of dairy product and calcium untakes and prostate cancer risk: A meta-analysis. J. Nat. Cancer Inst..

[28] Koizumi T., Li Z.G., Tatsumoto H. (1992). DNA damaging activity of cadmium in Leydig cells, a target cell population for cadmium carcinogenesis in the rat testis. Toxicol. Lett..

[29] Harries L.W., Stubbins M.J., Forman D. (1997). Identification of genetic polymorphisms at the glutathione S-transferase Pi locus and association with susceptibility to bladder, testicular and prostate cancer. Carcinogenesis.

[30] Manecksha R.P., Fitzpatrick J.M. (2009). Epidemiology of testicular cancer. BJU Int..

[31] Zatoński W., Przewoźniak K. (2001). Tytoń, a zdrowie w Polsce. Gaz. Farmaceut..

[32] Darago A., Chmielnicka J. (2004). Znaczenie kadmu, selenu, cynku i miedzi w rozwoju nowotworów gruczołu krokowego. Nowotwory.

[33] Didkowska J., Wojciechowska U., Zatoński W. (2009). Prediction of Cancer Incidence and Mortality in Poland up to the Year 2025.

[34] Brockmoller J., Cascorbi I., Henning S., Meisel C., Roots I. (2000). Molecular Genetics of Cancer Susceptibility. Pharmacology.

[35] Sivonova M., Waczulikova I., Dobrota D., Matakova T., Hatok J., Racay P., Kliment J. (2009). Polymorphisms of glutatione-S-transferase M1, T1, P1 and the risk of prostate cancer: A case-control study. J. Exp. Clin. Cancer Res..

[36] Choi J.D., Lee J.S. (2013). Interplay between epigenetics and genetics in cancer. Genom. Inf..

[37] Kumar V.L., Majumder P.K. (1995). Prostate gland: Structure, functions and regulation. Int. Urol. Nephrol..

[38] Seisen T., Roupret M., Faix A., Droupy S. (2012). The prostate gland: A crossroad between the urinary and the seminal tracts. Prog. Urol..

[39] Senkus-Konefka E., Antoniewicz A., Borkowski A., Borówka A., Demkow T., Dobruch J., Fijuth J., Jassem J., Krzakowski M., Ligaj M. (2007). Recommendations on the management of prostate cancer- a round table conference. Onkol. Prakt. Klin..

[40] Kral M., Rosinska V., Student V., Grepl M., Hrabec M., Bouchal J. (2011). Genetic Determinants of Prostate Cancer: A Reviev. Biomed. Pap. Med. Fac. Univ. Palacky Olomouc Czech Repub..

[41] Stachurska A., Wronka M., Kowalczyńska H.M. (2007). Rak gruczołu krokowego w badaniach in vitro: Charakterystyka linii komórkowych PC3, DU145 i LNCaP. Urol. Pol..

[42] Szot W., Kostkiewicz M., Zając J., Owoc A., Bojar I. (2014). Prostate cancer in patients from rural and suburban areas- PSA value, Gleason score and presence of metastases in bone scan. Ann. Agric. Environ. Med..

[43] Nurzyński P. (2011). Prostata. Schorzenie, leczenie, profilaktyka. Wyd. Weltbild..

[44] Daniyal M., Siddiqui Z.A., Akram M., Asif H.M., Sultana S., Khan A. (2014). Epidemiology, Etiology, Diagnosis and Treatment of Prostate Cancer. Asian Pac. J. Cancer Prev..

[45] Naserghandi A., Abedi A.R., Zavareh M.A.T., Shahsavari M.J., Shadravan M.M., Mirzaei S., Kakaee M.A., Allameh F. (2025). Prostate Cancer and the Rise of Focal Laser Therapies: A Narrative Review of Benefits and Limitations. J. Lasers Med. Sci..

[46] Rai V. (2025). Immune Checkpoint Inhibitor Therapy for Prostate Cancer: Present and Future Prospectives. Biomolecules.

[47] Zeigler-Johnson C.M., Spangler E., Jalloh M., Gueye S.M., Rennert H., Rebbeck T.R. (2008). Genetic susceptibility to prostate cancer in men of African descent: Implications for global disparities in incidence and outcomes. Can. J. Urol..

[48] Mandair D., Rossi R.E., Pericleous M., Whyand T., Caplin M.E. (2014). Prostate cacer and the influnce of dietary factors and supplements: A systematic review. Nutr. Metab..

[49] Didkowska J., Wojciechowska U. (2015). Zachorowania i Zgony na Nowotwory Złośliwe w Polsce. Krajowy Rejestr Nowotworów, Centrum Onkologii-Inst. im. M. Skłodowskiej-Curie. Nowotwory. J. Oncol..

[50] Chłosta P. (2009). Hormonal treatment of the prostate cancer patients. Geriatria.

[51] Mostafavi Zadeh S.M., Tajik F., Moradi Y., Kiani J., Ghods R., Madjd Z. (2022). Impact of COVID-19 pandemic on screening and diagnosis of patients with prostate cancer: A systematic review protocol. BMJ Open.

[52] Chu F., Chen L., Guan Q., Chen Z., Ji Q., Ma Y., Ji J., Sun M., Huang T., Song H. (2025). Global burden of prostate cancer: Age-period-cohort analysis from 1990 to 2021 and projections until 2040. World J. Surg. Oncol..

[53] Zi H., Liu M.Y., Luo L.S., Huang Q., Luo P.C., Luan H.H., Huang J., Wang D.Q., Wang Y.B., Zhang Y.Y. (2024). Global burden of benign prostatic hyperplasia, urinary tract infections, urolithiasis, bladder cancer, kidney cancer, and prostate cancer from 1990 to 2021. Mil. Med. Res..

[54] Lichtenstein P., Holm N.V., Verkasalo P.K., Illiadou A., Kaprio J., Koskenvuo M., Pukkala E., Skytthe A., Hemminki K. (2000). Environmental and heritable factors in the causation of cancer- analyses of cohorts of twins from Sweden, Denmark, and Finland. N. Engl. J. Med..

[55] Fu F., Yu Y., Wang B., Zhao X., Wang N., Yin J., Wu K., Zhou Q. (2025). Prostate and urinary microbiomes in prostate cancer development: Focus on *Cutibacterium acnes*. Front. Cell. Infect. Microbiol..

[56] Radej S., Szewc M., Maciejewski R. (2022). Prostate Infiltration by Treg and Th17 Cells as an Immune Response to *Propionibacterium acnes* Infection in the Course of Benign Prostatic Hyperplasia and Prostate Cancer. Int. J. Mol. Sci..

[57] Peinado B.R.R., Nazário R.M.F., Frazão D.R., Né Y.G.S., Bittencourt L.O., Fagundes N.C.F., Mesquita C.M., Rösing C.K., de Souza-Rodrigues R.D., Paranhos L.R. (2025). Is There Any Association Between Periodontitis and Prostatic Alterations? A Systematic Review. Prostate.

[58] Butler L.M., Wong A.S., Koh W.P., Wang R., Yuan J.M., Yu M.C. (2010). Calcium intake increases risk of prostate cancer among Singapore Chinese. Cancer Res..

[59] Ociepa-Kubicka A., Ociepa E. (2012). Toksyczne oddziaływanie metali ciężkich na rośliny, zwierzęta i ludzi. Inż. Ochr. Środ..

[60] Wilhelm M., Ewers U., Schulz C. (2004). Revised and new reference values for some trace elements in blood and urine for human biomonitoring in environmental medicine. Int. J. Hyg. Environ. Health.

[61] Heitland P., Köster H.D. (2006). Biomonitoring of 30 trace elements in urine of children and adults by ICP-MS. Clin. Chim. Act..

[62] Potts C.L. (1965). Cadmium proteinuria. The health of battery workers exposed to cadmium oxide dust. Ann. Occup. Hyg..

[63] Elinder C.G., Kjellstrom T., Hogstedt C., Andersson K., Spang G. (1985). Cancer mortality of cadmium workers. Br. J. Ind. Med..

[64] Białkowski K., Białkowska A., Kasprzak K.S. (1999). Cadmium(II), unlike nickel(II), inhibits 8-oxo-dGTPase activity and increases 8-oxo-dG level in DNA of the rat testis, a target organ for cadmium(II) carcinogenesis. Carcinogenesis.

[65] Godt J., Scheidig F., Grosse-Siestrup C., Esche V., Brandenburg P., Reich A., Groneberg D.A. (2006). The toxicity of cadmium and resulting hazards for human health. J. Occup. Med. Toxicol..

[66] Nriagu J.O., Boughanen M., Linder A., Howe A., Grant Ch Rattray R., Vutchkov M., Lalor G. (2009). Levels of As, Cd, Pb, Cu, Se and Zn in bovine kidneys and livers in Jamaica. Ecotoxicol. Environ. Saf..

[67] Romanowicz-Makowska H., Forma E., Bryś M., Krajewska W.M., Smolarz B. (2011). Concentration of cadmium, nickel and aluminum in female breast cancer. Pol. J. Pathol..

[68] Czeczot H., Skrzycki M. (2015). Kadm-pierwiastek całkowicie zbędny dla organizmu Cadmium-element completely unnecessary for the organism. Post. Hig. Med. Dośw..

[69] Leitzmann M.F., Stampfer M.J., Wu K., Colditz G.A., Willett W.C., Giovannucci E.L. (2003). Zinc supplement use and risk of prostate cancer. J. Nat. Cancer Inst..

[70] Brown K.H., Wuehler S.E., Peerson J.M. (2001). The importance of zinc in human nutrition and estimation of the global prevalence of zinc deficiency. Food Nutr. Bull..

[71] Kabata-Pendias A., Szteke B. (2004). Żelazo i Mangan w Środowisku—Problemy Ekologiczne i Metodyczne.

[72] Kabata-Pendias A., Pendias H. (2004). Biogeochemia Pierwiastków Śladowych.

[73] Bhowmik D., Chiranjib K.P. (2010). A potential medicinal importance of zinc in human health and chronic. Int. J. Pharm..

[74] Plum L.M., Rink L., Haase H. (2010). The Essential Toxin: Impact of Zinc on Human Health. Int. J. Environ. Res. Public Health.

[75] Gapys B., Raszeja-Specht A., Bielarczyk H. (2014). Role of zinc in phisiological and pathological processes of the body. Diagn. Lab..

[76] Dobrowolski Z., Drewniak T., Cichocki T., Kwiatek W. (2000). Stężenie miedzi i cynku oraz współczynnik Cu/Zn w surowicy krwi oraz tkance raka jasnokomórkowego nerki. Urol. Pol..

[77] Chasapis C.T., Loutsidou A.C., Spiliopoulou C.A., Stefanidou M.E. (2012). Zinc and human health: An update. Arch. Toxicol..

[78] Coradduzza D., Sanna A., Di Lorenzo B., Congiargiu A., Marra S., Cossu M., Tedde A., De Miglio M.R., Zinellu A., Mangoni A.A. (2025). Associations between plasma and urinary heavy metal concentrations and the risk of prostate cancer. Sci. Rep..

[79] Wu H., Wang M., Raman J.D., McDonald A.C. (2021). Association between urinary arsenic, blood cadmium, blood lead, and blood mercury levels and serum prostate-specific antigen in a population-based cohort of men in the United States. PLoS ONE.

[80] Krzywy I., Krzywy E., Pastuszak-Gabinowska M., Brodkiewicz A. (2010). Lead- is there something to be affraid of?. Ann. Acad. Med. Stet..

[81] Kabata-Pendias A., Szteke B. (1998). Ołów w Środowisku—Problemy Ekologiczne i Metodyczne.

[82] Jemal A., Graubard B.I., Devesa S.S., Flegal K.M. (2002). The association of blood lead level and cancer mortality among whites in the United States. Environ. Health Perspect..

[83] Navarro Silvera S.A., Rohan T.E. (2007). Trace elements and cancer risk: A review of the epidemiologic evidence. Cancer Causes Control..

[84] Merian E. (1991). Metals and Their Compounds in the Environment.

[85] Pais I., Benton Jones J. (2000). The Handbook of Trace Elements.

[86] Fraga C.G. (2005). Relevance, essentiality and toxicity of trace elements in human health. Mol. Asp. Med..

[87] He Z.L., Yang X.E., Stoffella P.J. (2005). Trace elements in agroecosystems and impacts on the environment. J. Trace Elem. Med. Biol..

[88] Lobinski R., Moulin C., Ortega R. (2006). Imaging and speciation of trace elements in biological environment. Biochimie.

[89] Kubiak T. (2013). Związki metabolizmu żelaza z rozwojem raka piersi u kobiet przed i po menopauzie. Przegl. Menopauz..

[90] Artym J. (2008). The role of lactoferrin in the iron metabolism. Post. Hig. Med. Dośw..

[91] Sussman H. (1992). Iron in cancer. Pathobiology.

[92] Daniel K.G., Harbach R.H., Guida W.C., Dou Q.P. (2004). Copper storage diseases: Menkes, Wilsons, and cancer. Front. Biosci..

[93] Stern B.R., Solioz M., Krewski D., Aggett P., Aw T., Baker S., Crump K., Dourson M., Haber L., Hertzberg R. (2007). Copper and human health: Biochemistry, genetics, and strategies for modeling dose-response relationships. J. Toxicol. Environ. Health.

[94] Gupta A., Lutsenko S. (2009). Human copper transporters: Mechanism, role in human diseases and therapeutic potential. Fut. Med. Chem..

[95] Kabata-Pendias A., Szteke B. (2012). Pierwiastki Śladowe w Geo- i Biosferze.

[96] Ghazaryan S. (2011). Role of copper for human organism. Eur. Med. Heal. Pharm. J..

[97] Guertin J. (2004). Toxicity and Health Effects of Chromium (All Oxidation States).

[98] Gibb H.J., Lees P.S., Pinsky P.F., Rooney B.C. (2000). Lung cancer among workers in chromium chemical production. Am. J. Indust. Med..

[99] Terpiłowska S., Zaporowska H. (2003). Chromium and its role in a prevention and therapy of some diseases. Ann. Univ. Mariae Curie-Skłodowska.

[100] Sobański L., Sprzęczka-Niedolaz M., Łebek G. (2007). Rola chromu w życiu człowieka. Bromat. Chem. Toksykol..

[101] Eastmond D.A., MacGregor J.T., Slesinski R.S. (2008). Trivalent chromium: Assessing the genotoxic risk of an essential trace element and widely used human and animal nutritional supplement. Crit. Rev. Toxicol..

[102] Kabata-Pendias A. (1993). Chrom, Nikiel i Glin w Środowisku—Problemy Ekologiczne i Metodyczne.

[103] Das K.K., Das S.N., Dhundasi S.A. (2008). Nickel, its adverse health effects & oxidative stress. Indian J. Med. Res..

[104] Zambelli B., Ciurli S. (2013). Nickel and human health. Met. Ions Life Sci..

[105] Szymańska-Chabowska A., Antonowicz-Juchniewicz J., Andrzejak R. (2004). Analiza stężeń wybranych markerów neoplazmatycznych u osób zawodowo narażonych na arsen i metale ciężkie. Med. Pracy.

[106] Kabata-Pendias A., Szteke B. (1994). Arsen i Selen w Środowisku—Problemy Ekologiczne i Metodyczne.

[107] Gawęda E. (2005). Arsenic and arsenic compounds in the working environment - hazards, occupational risk assessment. BP Bezpieczeństwo Pr..

[108] Guha Muzumder D.N. (2008). Chronic arsenic toxicity & human health. Indian J. Med. Res..

[109] Kapaj S., Peterson H., Liber K., Bhattacharya P. (2006). Human health effects from chronic arsenic poisoning- a review. J. Environ. Sci. Health A.

[110] Kabata-Pendias A., Żmudzki J. (1992). Rtęć w Środowisku—Problemy Ekologiczne i Metodyczne.

[111] Kazantzis G. (2002). Mercury exposure and early effects: An overview. Med. Lav..

[112] Hyman M.H. (2004). The impact of mercury on human health and the environment. Altern. Ther..

[113] Zahir F., Rizwi S.J., Haq S.K., Khan R.H. (2005). Low dose mercury toxicityand human health. Environ. Toxicol. Pharmacol..

[114] Cyran M. (2013). Effects of environmental exposure to mercury of the functioning of the human body. Med. Srod..

[115] Crespo-Lopez M.E., Macedo G.L., Pereira S.I.D., Arrifano G.P.F., Picanco-Diniz D.L.W., Nascimento J.L.M., Herculano A.M. (2009). Mercury and human genotoxicity: Critical considerations and possible molecular mechanisms. Pharm. Res..

[116] Tshoni U.A., Mbonane T.P., Rathebe P.C. (2024). The Role of Trace Metals in the Development and Progression of Prostate Cancer. Int. J. Mol. Sci..

[117] Dróżdż J., Kamiński P., Bombolewska K. (2012). The influence of xenobiotics and environmental selection upon carcinogenic changes in human male reproductive system. Environ. Biotechnol..

[118] Dróżdż J., Bombolewska K., Kamiński P., Bogdzińska M. (2013). Polymorphism of the glutatione S-transferases in prostate cancer. Copernic. Lett..

[119] Dróżdż-Afelt J. (2015). Glutathione S-Transferase Polymorphism and Antioxidant Defense Reactions and Their Relationship with Trace Element Levels in Prostate Cancer Patients. Ph.D. Thesis.

[120] Dróżdż-Afelt J.M., Koim-Puchowska B., Kłosowski G., Kamiński P. (2020). Polymorphism of glutathione S-transferase in the population of Polish patients with carcinoma of the prostate. Environ. Sci. Pollut. Res..

[121] Dróżdż-Afelt J.M., Koim-Puchowska B., Kamiński P. (2022). Analysis of oxidative stress indicators in Polish patients with prostate cancer. Environ. Sci. Pollut. Res..

[122] Dróżdż-Afelt J.M., Koim-Puchowska B., Kamiński P. (2024). Concentration of trace elements in blood of Polish patients with prostate cancer. Environ. Toxicol. Pharmacol..

[123] Bombolewska K., Dróżdż J., Kamiński P., Bogdzińska M., Koim-Puchowska B. (2013). The role of molecular predictors of the diagnosis and treatment in women with gynecological cancer. Copernic. Lett..

[124] Benbrahim-Tallaa L., Waalkes M.P. (2008). Inorganic Arsenic and Human Prostate Cancer. Environ. Health Persp..

[125] Goullé J.P., Mahieu L., Castermant J., Neveu N., Bonneau L., Lainé G., Bouige D., Lacroix C. (2005). Metal and metalloid multi-elementary ICP-MS validation in whole blood, plasma, urine and hair. Reference values. Forensic Sci. Int..

[126] Alimonti A., Bocca B., Mannella E., Petrucci F., Zennaro F., Cotichini R., D’Ippolito C., Agresti A., Caimi S., Forte G. (2005). Assessment of reference values for selected elements in a healthy urban population. Ann. Inst. Super. Sanita.

[127] Forrer R., Gautschi K., Lutz H. (2001). Simultaneous measurement of the trace elements Al, As, B, Be, Cd, Co, Cu, Fe, Li, Mn, Mo, Ni, Rb, Se, Sr, and Zn in human serum and their reference ranges by ICP-MS. Biol. Trace Elem. Res..

[128] Schweitzer L., Cornett C. (2008). Determination of Heavy Metals in Whole Blood Using Inductively-Coupled Plasma-Mass Spectrometry: A Comparison of Microwave and Dilution Techniques. Big M..

[129] Litwin I., Lis P., Maciaszczyk-Dziubińska E. (2009). Dwie twarze arsenu. Kosmos.

[130] Mortada W.I., Sobh M.A., El-Defrawy M.M. (2004). The exposure to cadmium, lead and mercury from smoking and its impact on renal integrity. Med. Sci. Monit..

[131] Agarwal A., Gupta S., Sikka S. (2006). The role of free radicals and antioxidants in reproduction. Curr. Opin. Obstet. Ginecol..

[132] Arsova-Sarafinovska Z., Eken A., Matevska N., Erdem O., Sayal A., Savaser A., Banev S., Petrovski D., Dzikova S., Georgiev V. (2009). Increased oxidative/nitrosative stress and decreased antioxidant enzyme activities in prostate cancer. Clin. Biochem..

[133] Mottaghipisheh J., Doustimotlagh A.H., Irajie C., Tanideh N., Barzegar A., Iraji A. (2022). The Promising Therapeutic and Preventive Properties of Anthocyanidins/Anthocyanins on Prostate Cancer. Cells.

[134] Bartosz G. (2003). Druga Twarz Tlenu. Wolne Rodniki w Przyrodzie.

[135] Czajka A. (2006). Wolne rodniki tlenowe a mechanizmy obronne organizmu. Now. Lek..

[136] Kulawiak-Gałąska D. (2006). Mechanizmy Wolnorodnikowego Działania Doksorubicyny w Układach Biologicznych. Ph.D. Thesis.

[137] Kalisz O., Wolski T., Gerkowicz M., Smorawski M. (2007). Reaktywne formy tlenu (RTF) oraz ich rola w patogenezie niektórych chorób. Ann. Univ. Mariae Curie-Skłodowska Lub.-Pol..

[138] Janicka A., Szymańska-Pastenak J., Bober J. (2013). Polimorfizm genów obrony antyoksydacyjnej a ryzyko rozwoju raka. Ann. Acad. Med. Stet..

[139] Djokic M., Radic T., Santric V., Dragicevic D., Suvakov S., Mihailovic S., Stankovic V., Cekerevac M., Simic T., Nikitovic M. (2022). The Association of Polymorphisms in Genes Encoding Antioxidant Enzymes GPX1 (rs1050450), SOD2 (rs4880) and Transcriptional Factor Nrf2 (rs6721961) with the Risk and Development of Prostate Cancer. Medicina.

[140] Lin H., Zhu X., Aucoin A.J., Fu Q., Park J.Y., Tseng T. (2023). Dietary and Serum Antioxidants Associated with Prostatic-Specific Antigen for Middle-Aged and Older Men. Nutrients.

[141] Ozmen H., Erulas F.A., Karatas F., Cukurovali A., Yalcin O. (2006). Comparison of the concentration of trace metals (Ni, Zn, Co, Cu and Se), Fe, vitamins A, C and E, and lipoperoxidation in patients with prostate cancer. Clin. Chem. Lab. Med..

[142] Kotrikadze N., Alibegashvili M., Zibzibadze M., Abashidze N., Chigogidze T., Managadze L., Artsivadze K. (2008). Activity and content of antioxidant enzymes in prostate tumors. Exp. Oncol..

[143] Gaweł S., Wardas M., Niedworok E., Wardas P. (2005). Dialdehyd malonowy (MDA) jako wskaźnik procesów peroksydacji lipidów w organizmie. Int. J. Adv. Integr. Med. Sci..

[144] Kulbacka J., Saczko J., Chwiłkowska A. (2009). Stres oksydacyjny w procesach uszkodzenia komórek. Pol. Merk. Lek..

[145] Skrzycki M., Czeczot H. (2005). Rola dysmutazy ponadtlenkowej w powstawaniu nowotworów. Post. Nauk. Med..

[146] Surapaneni K.M., Ramana G.V. (2006). Lipoperoxidation and antioxidant status in patients with carcinoma of prostate. Indian J. Physiol. Pharmacol..

[147] Kumaraguruparan R., Subapriya R., Kabalimoorthy J., Nagini S. (2002). Antioxidant profile in circulation of patients with fibrodenoma and adenocarcinoma of breast. Clin. Biochem..

[148] Polat M.F., Taysi S., Gul M., Yilmax I., Bakan E., Erdogan F. (2002). Oxidant-antioxidant status in blood of patients with malignant breast tumor and benign breast disease. Cell Biochem. Funct..

[149] McIlwain C.C., Townsend D.M., Tew K.D. (2006). Glutathione S-transferase polymorphisms: Cancer incidence and therapy. Oncogene.

[150] Lavender N.A., Benford M.L., VanCleave T.T. (2009). Examination of polymorphic glutathione S-transferase (GST) genes, tobacco smoking and prostate cancer risk among Men of African Descent: A case-control study. BMC Cancer.

[151] Pagoni M., Zogopoulos V.L., Kontogiannis S., Tsolakou A., Zoumpourlis V., Tsangaris G.T., Fokaefs E., Michalopoulos J., Tsatsakis A.M., Drakoulis N. (2025). Integrated Pharmacogenetic Signature for the Prediction of Prostatic Neoplasms in Men with Metabolic Disorders. Cancer Genom. Proteom..

[152] Grönberg H. (2003). Prostate cancer epidemiology. Lancet.

[153] Gong M., Dong W., Shi Z., Xu Y., Ni W., An R. (2012). Genetic Polymorphisms of GSTM1, GSTT1 GSTP1 with Prostate Cancer Risk: A Meta-Analysis of 57 Studies. PLoS ONE.

[154] Cai Q., Wang Z., Zhang W., Guo X., Shang Z., Jiang N., Tian J., Niu Y. (2014). Association between glutathione S-transferases M1 and T1 gene polymorphisms and prostate cancer risk: A systematic review and meta-analysis. Tumor Biol..

[155] Valko M., Rhodes C.J., Moncol J., Izakovic M.M., Mazur M. (2006). Free radicals, metals and antioxidants in oxidative stress-induced cancer. Chem. Biol. Interact..

[156] Ouadri Q., Sameer A.S., Shah Z.A., Hamid A., Alam S., Manzoor S., Siddiqi M.A. (2011). Genetic polymorphism of the glutathione-S-transferase P1 gene (GSTP1) and susceptibility to prostate cancer in the Kashmiri population. Genet. Mol. Res..

[157] Przybyszewski W.M., Rzeszowska-Wolny J. (2009). Stres oksydacyjny w procesach przerostu i kancerogenezy gruczołu sterczowego. Post. Hig. Med. Dośw..

[158] Fukuda H., Ebara M., Yamada H., Arimoto M., Okabe S., Obu M., Saisho H. (2004). Trace elements and cancer. JMAJ Jpn. Med. Assoc. J..

[159] Ntais C., Polycarpou A., Ioannidis J.P. (2005). Association of GSTM1, GSTT1, and GSTP1 gene polymorphisms with the risk of prostate cancer: A meta-analysis. Cancer Epidemiol. Biomark. Prev..

[160] Dong J. (2001). Chromosomal deletions and tumor suppressor genes in prostate cancer. Cancer Metastasis Rev..

[161] Kozłowska J., Łaczmańska I. (2010). Niestabilność genetyczna- jej znaczenie w procesie powstawania nowotworów oraz diagnostyka laboratoryjna. Nowotwory.

[162] Schleutker J., Matikainen M., Smith J., Koivisto P., Baffoe-Bonnie A., Kainu T., Gillanders T., Sankila R., Pukkala E., Carpten J. (2000). A Genetic Epidemiological Study of Hereditary Prostate Cancer (HPC) in Finland: Frequent HPCX Linkage in Families with Late-onset Disease. Clin. Cancer Res..

[163] Bratt O. (2002). Hereditary prostate cancer: Clinical Aspects. J. Urol..

[164] Caceres D.D., Iturrieta J., Acevedo C., Huidobro C., Varela N., Quinones L. (2005). Relationship among metabolizing genes, smoking and alcohol used as modifier factors on prostate cancer. Eur. J. Epidemiol..

[165] Cybulski C., Gliniewicz B., Sikorski A., Lubiński J. (2008). Genetyka kliniczna raka prostaty. Post. Nauk. Med..

[166] Parent M.E., Siemiatycki J. (2001). Occupation and prostate cancer. Epidemiol. Rev..

[167] Alavanja M.C.R., Samanic C., Dosemeci M., Lubin J., Tarone R., Lynch C.h.F., Knott Ch Kent T., Hoppin J.A., Barker J., Coble J. (2003). Use of Agricultural Pesticides and Prostate Cancer Risk in the Agricultural Health Study Cohort. Am. J. Epidemiol..

[168] Bostwick D.G., Burke H.B., Djakiew D., Euling S., Ho S., Landolph J., Morrison H., Sonawane B., Shifflett T., Waters D.J. (2004). Human prostate cancer risk factors. Cancer.

[169] Peng H.Q., Hogg D., Malkin D., Bailey D., Gallie B.L., Bulbul M., Jewett M., Buchanan J., Goss P.E. (1993). Mutations of the p53 gene do not occur in testis cancer. Cancer Res..

[170] Aydin M., Bozkurt A., Cikman A., Gulhan B., Karabakan M., Gokce A., Alper M., Kara M. (2017). Lack of evidence of HPV etiology of prostate cancer following radical surgery and higher frequency of the Arg/Pro genotype in turkish men with prostate cancer. Int. Braz. J. Urol..

[171] Kirchhoff T., Kauff N.D., Mitra N., Nafa K., Huang H., Palmer C., Gulati T., Wadsworth E., Donat S., Robson M.E. (2004). BRCA mutations and risk of prostate cancer in Ashkenazi Jews. Clin. Cancer Res..

[172] Agalliu I., Gern R., Leanza S., Burk R.D. (2009). Associations of high-grade prostate cancer with BRCA1 and BRCA2 founder mutations. Clin. Cancer Res..

[173] Sheehan B.J., Edwards B., Soto Medrano I., El-Saidi M.A., Zaidan W.R., El-Ezzi A.A., Kuddus R.H. (2025). Association between two single nucleotide polymorphisms of the Prostaglandin-Endoperxide Synthase 1 and 2 genes and cell proliferative prostatic diseases in Lebanon. Oncotarget.

[174] Deng J., Xu L., Zhou J., Huang H. (2025). Associations between XRCC1-Arg399Gln polymorphism and the risk of prostate cancer: An updated meta-analysis. Amino Acids..

[175] Goel K., Venkatappa V., Krieger K.L., Chen D., Sreekumar A., Gassman N.R. (2025). PARP inhibitor response is enhanced in prostate cancer when XRCC1 expression is reduced. NAR Cancer.

[176] Marahatta S.B., Punyarit P., Bhudisawasdi V., Paupairoj A., Wongkham S., Petmitr S. (2006). Polymorphism of glutathione S-transferase omega gene and risk of cancer. Cancer Lett..

[177] Mo Z., Gao Y., Cao Y. (2009). An updating meta-analysis of the GSTM1, GSTT1, and GSTP1 polymorphisms and prostate cancer: A HuGE review. Prostate.

[178] Kwon D.D., Lee J.W., Han D.J. (2011). Relationship between the Glutathione-S-Transferase P1, M1, and T1 Genotypes and Prostate Cancer Risk in Korean Subjects. Korean J. Urol..

[179] Nock N.L., Bock C., Neslund-Dudas C.I. (2009). Polymorphisms in glutathione S-transferase genes increase risk of prostate cancer biochemical recurrence differentially by ethnicity and disease severity. Cancer Causes Control..

[180] Strange R.C., Spiteri M.A., Ramachandran S., Fryer A.A. (2001). Glutathione-S-transferase family of enzymes. Mutat. Res. Fundam. Mol. Mech. Mutag..

[181] Mitrunen K., Jourenkova N., Kataja V., Eskelinen M., Kosma V.M., Benhamou S., Hirvonen A. (2001). Glutathione S-transferase M1, M3, P1, and T1 genetic polymorphisms and susceptibility to breast cancer. Cancer Epidemiol. Biomark. Prev..

[182] Rebbeck T.R. (1997). Molecular epidemiology of the human glutathione S-transferase genotypes GSTM1 and GSTT1 in cancer susceptibility. Cancer Epidemiol. Biomark. Prev..

[183] Hayes J.D., Strange R.C. (2000). Glutathione S-transferase polymorphisms and their biological consequences. Pharmacology.

[184] http://www.ncbi.nlm.nih.gov/gene/2944.

[185] Medeiros R., Vasconcelos A., Costa S., Pinto D., Ferreira P., Lobo F., Lopes C. (2004). Metabolic susceptibility genes and prostate cancer risk in a southern European population: The role of glutathione S-transferases GSTM1, GSTM3, and GSTT1 genetic polymorphisms. Prostate.

[186] Hashibe M., Brennan P., Strange R.C., Bhisey R., Cascorbi I., Lazarus P., Boffetta P. (2003). Meta-and pooled analyses of GSTM1, GSTT1, GSTP1, and CYP1A1 genotypes and risk of head and neck cancer. Cancer Epidemiol. Biomark. Prev..

[187] http://www.ncbi.nlm.nih.gov/gene/2952.

[188] Gsur A., Haidinger G., Hinteregger S., Bernhofer G., Schatzl G., Madersbacher S., Micksche M. (2001). Polymorphisms of glutathione-S-transferase genes (GSTP1, GSTM1 and GSTT1) and prostate-cancer risk. Int. J. Cancer.

[189] Kehrer J.P., Biswal S.S. (2000). The Molecular Effects of Acrolein. Toxicol. Sci..

[190] Beer T.M., Evans A.J., Hough K.M., Lowe B.A., McWilliams J.E., Henner W.D. (2001). Polymorphisms of GSTP1 and related genes and prostate cancer risk. Prost. Cancer Prost. Dis..

[191] Rybicki B.A., Neslund-Dudas C., Nock N.L., Schultz L.R., Eklund L., Rosbolt J., Monaghan K.G. (2006). Prostate cancer risk from occupational exposure to polycyclic aromatic hydrocarbons interacting with the GSTP1 Ile105Val polymorphism. Cancer Detect. Prev..

[192] Buchard A., Sanchez J.J., Dalhoff K., Morling N. (2007). Multiplex PCR detection of GSTM1, GSTT1, and GSTP1 gene variants: Simultaneously detecting GSTM1 and GSTT1 gene copy number and the allelic status of the GSTP1 Ile105Val genetic variant. J. Mol. Diagn..

[193] http://www.ncbi.nlm.nih.gov/gene/2950.

[194] Nam R.K., Zhang W.W., Trachtenberg J., Jewett M.A., Emami M., Vesprini D., Chu W., Ho M., Sweet J., Evans A. (2003). Comprehensive assessment of candidate genes and serological markers for the detection of prostate cancer. Cancer Epidemiol. Biomark. Prev..

[195] Mittal R.D., Mishra D.K., Mandhani A. (2006). Evaluating polymorphic status of glutathione S-transferase genes in blood and tissue samples of prostate cancer patients. Asian Pacific. J. Cancer Prev..

[196] Wei B., Zhou Y., Xu Z., Ruan J., Cheng H., Zhu M., Hu Q., Jin K., Yan Z., Zhou D. (2013). GSTP1 Ile/Val polymorphism and prostate cancer risk: Evidence from a meta-analysis. PLoS ONE.

[197] Gundacker C., Komarnicki G., Jagiello P., Gencikova A., Dahmen N., Wittmann K.J., Gencik M. (2007). Glutathione-S-transferase polymorphism, metallothionein expression, and mercury levels among students in Austria. Sci. Total Environ..

[198] Klautau-Guimaraes M., Dascencao R., Caldart F.A., Grisolia C.K., de Souza J.R., Barbosa A.C., Cordeiro C.M.T., Ferrari I. (2005). Analysis of genetic susceptibility to mercury contamination evaluated through molecular biomarkers in at-risk Amazon Amerindian populations. Genet. Mol. Biol..

[199] Brambila E., Liu J., Morgan D.L., Beliles R.P., Waalkes M.P. (2002). Effect of mercury vapor exposure on metallothionein and glutathione stransferase gene expression in the kidney of nonpregnant, pregnant, and neonatal rats. J. Toxicol. Environ. Health.

[200] Barcelos G.R.M., Grotto D., de Marco K.C., Valentini J., van Helvoort Langert A., de Oliveira A.A.S., Garcia S.C., Braga G.U.L., Engstrom K.S., de Syllos Colus I.M. (2013). Polymorphisms in glutathione-related genes modify mercury concentrations and antioxidant status in subjects environmentally exposed to methylmercury. Sci. Total Environ..

[201] Khansakorn N., Wongwit W., Tharnpoophasiam P., Hengprasith B., Suwannathon L., Chanprasetyothin S., Sura T., Kaojarern S., Sritara P., Sirivarasai J. (2012). Genetic Variations of Glutathione S-Transferase Influence on Blood Cadmium Concentration. J. Toxicol..

[202] Kulikowska-Karpińska E., Popławski D., Gałażyn-Sidorczuk M., Rogalska J. (2009). Activity of antioxidant enzymes and lipoperoxidation in the pancreas of rats exposed to cadmium. Ochr. Środ. Zas. Nat..

[203] Koyu A., Gokcimen A., Ozguner F., Bayram D.S., Kocak A. (2006). Evaluation of the effects of cadmium on rat liver. Mol. Cell. Chem..

[204] Wilk M., Gworek B. (2009). Heavy metals in sewage sludge. Ochr. Środ. Zas. Nat..

[205] Birben E., Sahiner U.M., Sackesen C., Erzurum S., Kalayci O. (2012). Oxidative Stress and Antioxidant Defense. World Allergy Organ. J..

[206] Nandi D., Patra R.C., Swarup D. (2006). Oxidative stress indices and plasma biochemical parameters during oral exposure to arsenic in rats. Food Chem. Toxicol..

[207] Surgiewicz J. (2009). Chrom i jego związki-metoda oznaczania. PiMOŚP Podstawy i Metody Oceny Środowiska Pracy.

[208] Kobal A.B., Horvat M., Prezelj M., Briski A.S., Krsnik M., Dizdarevic T., Mazej D., Falnoga I., Stiblij V., Arneric N. (2004). The impact of long-term past exposure to elemental mercury on antioxidative capacity and lipoperoxidation in mercury miners. J. Trace Elem. Med. Biol..

[209] Zhou X., Jiao D., Dou M., Chen J., Li Z., Li Y., Liu J., Han X. (2019). Association of glutathione-S-transferase p1 gene promoter methylation and the incidence of prostate cancer: A systematic review and meta-analysis. J. Cancer Res. Clin. Oncol..

[210] Liu D., Che B., Chen P., He J., Mu Y., Chen K., Zhang W., Xu S., Tang K. (2022). GSTT1, an increased risk factor for prostate cancer in patients with metabolic syndrome. J. Clin. Lab. Anal..

[211] Seven D., Dalan A.B., Bayrak Ö.F. (2025). Targeting GSTM3 for therapeutic potential in advanced prostate cancer. BMC Cancer.

[212] Zhang Y., Meng X., Ma Z., Sun Z., Wang Z. (2023). Association of Androgen-Receptor Gene Mutations with the Copy Number of Androgen-Receptor Silk Protein A Complex and Glutathione-S-Transferases T1 and M1 in Prostate Cancer Patients. Genet. Res..

[213] Benabdelkrim M., Djeffal O., Berredjem H. (2018). *GSTM1* and *GSTT1* Polymorphisms and Susceptibility to Prostate Cancer: A Case-Control Study of the Algerian Population. Asian Pac. J. Cancer Prev..

[214] Charkiewicz A.E., Omeljaniuk W.J., Garley M., Nikliński J. (2025). Mercury Exposure and Health Effects: What Do We Really Know?. Int. J. Mol. Sci..

[215] Coradduzza D., Congiargiu A., Azara E., Mammani I.M.A., De Miglio M.R., Zinellu A., Carru C., Medici S. (2024). Heavy metals in biological samples of cancer patients: A systematic literature review. Biometals.

[216] Balali-Mood M., Eizadi-Mood N., Hassanian-Moghaddam H., Etemad L., Moshiri M., Vahabzadeh M., Sadeghi M. (2025). Recent advances in the clinical management of intoxication by five heavy metals: Mercury, lead, chromium, cadmium and arsenic. Heliyon.

[217] Sochacka M., Hoser G., Remiszewska M., Suchocki P., Sikora K., Giebułtowicz J. (2024). Effect of Selol on Tumor Morphology and Biochemical Parameters Associated with Oxidative Stress in a Prostate Tumor-Bearing Mice Model. Nutrients.

[218] Lin Y.C., Ku C.C., Wuputra K., Wu D.C., Yokoyama K.K. (2024). Vulnerability of Antioxidant Drug Therapies on Targeting the Nrf2-Trp53-Jdp2 Axis in Controlling Tumorigenesis. Cells.

[219] Liou G.Y., C’lay-Pettis R., Kavuri S. (2024). Invelvement of Reactive Oxygen Species in Prostate Cancer and Its Disparity in African Descendants. Int. J. Mol. Sci..

[220] Banas A., Kwiatek W.M., Banas K., Gajda M., Pawlicki B., Cichocki T. (2010). Correlation of concentrations of selected trace elements with Gleason grade of prostate tissues. J. Biol. Inorg. Chem..

[221] Rahmati S., Azami M., Delpisheh A., Hafezi Ahmadi M.R., Sayehmiri K. (2018). Total Calcium (Dietary and Supplementary) Intake and Prostate Cancer: A Systematic Review and Meta-Analysis. Asian Pac. J. Cancer Prev..

[222] Maly I.V., Hofmann W.A. (2018). Calcium and Nuclear Signaling in Prostate Cancer. Int. J. Mol. Sci..

[223] Kohli M., Tindall D.J. (2010). New Developments in the Medical Management of Prostate Cancer. Mayo Clin. Proc..

[224] Udensi U.K., Tchounwou P.B. (2016). Oxidative Stress in prostate hyperplasia and carcinogenesis. J. Exp. Clin. Cancer Res..

[225] Nelson W.G., Brawley O.W., Isaacs W.B., Platz E.A., Yegnasubramanian S., Sfanos K.S., Lotan T.L., De Marzo A.M. (2022). Health inequity drives disease biology to create disparities in prostate cancer outcomes. J. Clin. Investig..

[226] Leitão C., Neto V., Silva L., Estrela M., Fardilha M., Roque F., Herdeiro M.T. (2025). Perceptions, Knowledge, and Attitudes of General Population About Prostate Cancer-Associated Risk Factors: A Systematic Review of Qualitative Studies Focusing on Lifestyle. Curr. Oncol. Rep..

